# Polyolefin blends with co-continuous architectures enabled by dynamic covalent crosslinking

**DOI:** 10.1126/sciadv.aee2328

**Published:** 2026-05-15

**Authors:** Eliza K. Neidhart, Stephanie M. Ribet, Taehyun A. Lee, Logan Kearney, Karen C. Bustillo, Eric A. Dailing, Mutian Hua, Colin Ophus, Sophia N. Fricke, Ah-Young Song, Jeffrey A. Reimer, Erik J. Alexanian, Joanna M. Atkin, Brett A. Helms, Frank A. Leibfarth

**Affiliations:** ^1^ Department of Chemistry, The University of North Carolina at Chapel Hill, Chapel Hill, NC 27599, USA.; ^2^ National Center for Electron Microscopy, Molecular Foundry, Lawrence Berkeley National Laboratory, Berkeley, CA 94720, USA.; ^3^ Chemical Sciences Division, Oak Ridge National Laboratory, Oak Ridge, TN 37830, USA.; ^4^ The Molecular Foundry, Lawrence Berkeley National Laboratory, Berkeley, CA 94720, USA.; ^5^ School of Environmental and Forest Sciences, University of Washington, Seattle, WA 98195, USA.; ^6^ Department of Materials Science and Engineering, Stanford University, Stanford, CA 94305, USA.; ^7^ Department of Chemical and Biomolecular Engineering, University of California Berkeley, Berkeley, CA 94720, USA.; ^8^ College of Chemistry Pines Magnetic Resonance Center, University of California Berkeley, Berkeley, CA 94720, USA.; ^9^ Materials Sciences Division, Lawrence Berkeley National Laboratory, Berkeley, CA 94720, USA.

## Abstract

Blending polymers produces brittle materials due to macrophase separation and poor interfacial adhesion, which is exemplified by mixtures of polyolefins. This presents a formidable challenge for the mechanical recycling of mixed plastic waste. Here, we demonstrate that dynamic covalent crosslinking of immiscible polyolefin blends creates macrophase separated co-continuous architectures, yet they display excellent mechanical properties, which challenges the conventional wisdom regarding morphology-property relationships in polymer blend compatibilization. We find that the position and orientation of dynamic crosslinks and their influence on crystallinity are key to understanding the structure-morphology-property relationships. In particular, high-resolution microscopy imaging reveals alignment of crystallite planes with strong orientational preference, particularly at polymer-polymer interfaces, which contribute to material performance. We further demonstrate that changes in crosslinker density and valency allow the properties of binary and ternary polyolefin blends to be tuned in a modular fashion.

## Teaser

Reversible bonds create unexpected architectures between immiscible plastics that result in tough and reprocessable materials.

## INTRODUCTION

**Fig. 1.**
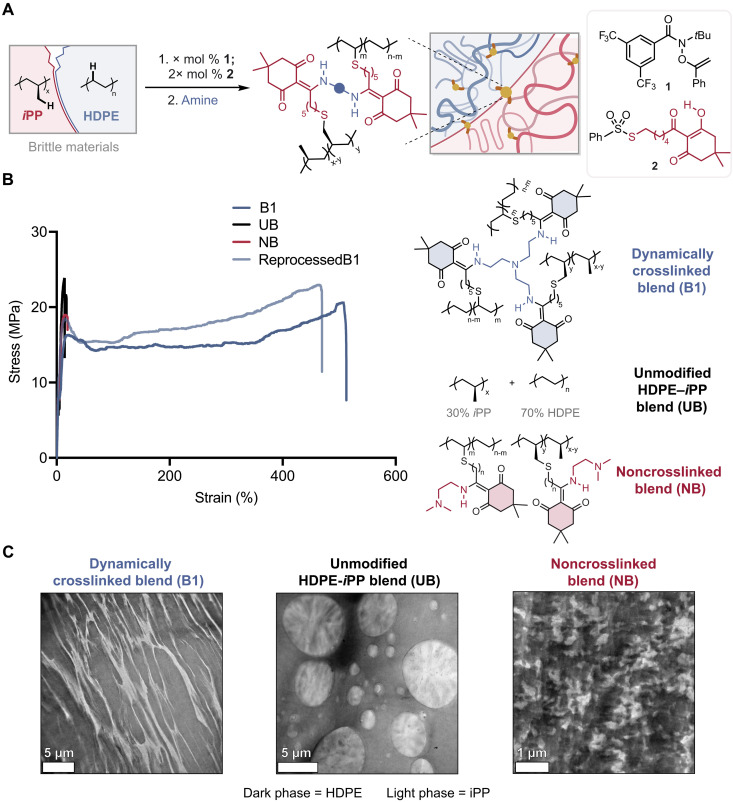
Performance advantages in dynamic covalent immiscible polymer blends with unusual co-continuous morphologies. (**A**) Concept, the preparation of diketoenamine crosslinked polyolefin blends from HDPE-*i*PP blends; (**B**) tensile properties of the dynamically crosslinked blend (**B1**), the unmodified HDPE-*i*PP blend (**UB**), the noncrosslinked blend (**NB**), and reprocessed **B1** (**C**) bright-field TEM of **B1**, **UB**, and **NB**.

The blending of two or more homopolymers promises a simple, cost-effective approach to tuning material properties. Yet in practice, most blends are brittle materials with poor mechanical properties in comparison to either blend constituent (*1*–*4*). The poor properties of polymer blends stem from thermodynamically disfavored mixing of polymers. Because the entropy of mixing scales inversely with the degree of polymerization (Δ*S*_mix_ ~ DP^−1^), its contribution to the overall free energy of mixing is small, resulting in macrophase separation and poor interfacial adhesion between phases, even for chemically similar polymers (Fig. 1A) (*1*, *5*, *6*). Blends of polyethylene and polypropylene are notable exemplars of this phenomenon (*5*–*8*). Together, these two polymers comprise more than 50% of global plastic production and are often comingled in waste streams, presenting a challenge for plastic recycling efforts (*9*–*17*). Improving the properties of polyolefin mixtures through compatibilization, defined as “a reduction of interfacial tension and domain size and an increase in interfacial adhesion that leads to an improvement in polymer blend mechanical performance,” would tackle fundamental, practical, and environmental challenges (*18*).

Here, we show that dynamic covalent crosslinking of immiscible polyolefins with diketoenamine bonds kinetically arrests striated, co-continuous blend architectures (Fig. 1). These striated morphologies do not conform to the morphologies typically associated with compatibilized blends, yet this approach offers noteworthy improvements to the mechanical properties and recyclability of immiscible polymer blends, which have been difficult to achieve (*19*, *20*). These advantages highlight the role of an extensive, dynamic network of stress-carrying diketoenamine covalent bonds at polymer-polymer interfaces and within each of the bulk phases, which we elucidate in blends of high-density polyethylene (HDPE) and isotactic polypropylene (*i*PP) with atomic force microscopy–based infrared (AFM-IR) spectroscopy and solid-state nuclear magnetic resonance (ssNMR) spectroscopy (*21*, *22*). Furthermore, by using four-dimensional scanning transmission electron microscopy (4D-STEM) imaging, we gain remarkable insight into the position and orientation of crystallites, revealing alignment of crystallites at the interface exclusive to the dynamically crosslinked blends (*23*). We identify a preference for the HDPE (010) and *i*PP (040) sets of planes to be aligned parallel to the boundary interface. We hypothesize that, during shear, when HDPE-*i*PP blends are thermally processed into molded parts, dynamic covalent bonds placed throughout not only alter phase morphology but also enable *i*PP crystallites to template HDPE crystallization, leading to the preferential orientation of crystallites in response to polymer flow. On the basis of these insights, we further introduce areal density of crosslinking and crosslinker valency as design features that can be used to tailor the properties of binary and ternary blends of polyolefins, e.g., increasing the toughness of HDPE-*i*PP and HDPE-LLDPE-*i*PP blends by 40 times and 445 times, respectively. Owing to the diversity of crosslinkers available, our findings portend a general strategy to tailor and enhance the mechanical performance of polymer blends. Together, this blending approach results in materials with excellent mechanical properties and an unconventional morphology that challenges conventional wisdom regarding structure-morphology-property relationships in compatibilized polymer blends, thus broadening opportunities for property enhancements through polymer blending.

Our work establishes structure-property-performance relationships for the development of performance-advantaged materials from widely available commodity polymers using dynamic covalent compatibilization. This represents a complementary approach to customized interface-segregating block copolymers, provides opportunities to tune properties based on functionalization density and crosslinker valency (*11*, *18*, *24*–*30*), and circumvents the degradation of polymer chains that is often encountered during reactive compatibilization of polyolefins (*31*, *32*). Prior work demonstrated increased miscibility of polymer phases using dynamic covalent bonds (*19*, *20*, *33*–*35*), but the resulting materials show minimal improvements in mechanical properties, especially for recalcitrant blends of polyolefins. There remains a need for selective functionalization strategies to decouple the impacts of dynamic crosslinking from chain scission. Further, there remains a lack of context for these findings, due to insufficient techniques to discern the position of important features, such as crystallinity and dynamic crosslinks with respect to the heterogeneous phase structure. In particular, while conventional transmission electron microscopy (TEM) reveals a change in morphology, it cannot easily elucidate crystallite position and orientation with respect to the phase interfaces or provide a rationale for the poor mechanical properties upon blending. In this work, we gain insight into the mechanism of compatibilization using techniques that provide positional insight and demonstrate how to improve the properties of polyolefin blends with pervasive dynamic covalent crosslinks. We also tie those properties to an in-depth understanding of the localization of bonds and polymer chains using AFM-IR spectromicroscopy and ssNMR and the impacts of the crosslinks and co-continuous blend architecture on crystallization and crystal alignment using differential scanning calorimetry (DSC) and 4D-STEM analysis, respectively. This allows us to quantify how pervasively and effectively dynamic covalent bonding exerts its influence on the resulting microstructure. This influence includes kinetic control over crystallization and orientation preferences at phase boundaries and a response to the direction of shear to lock in previously inaccessible but highly useful morphologies.

## RESULTS

Diketoenamines are a versatile class of dynamic covalent bonding motifs, whose chemistry can be tailored to access a range of properties, while being mechanically reprocessable and chemically recyclable (*36*–*41*). Here, our design strategy was to install triketones onto various polyolefins as reactive sites for spontaneous condensation with multifunctional small-molecule amines to form diketoenamine linkages (crosslinks) between polymer chains. To install triketones on HDPE and *i*PP, we performed amidyl radical-mediated C–H functionalization using an *O*-alkenyl hydroxamate (**1**) and a thiosulfonate triketone radical trap (**2**) (Fig. 1A and fig. S1). This approach limited polymer chain scission and chain coupling events, owing to the selectivity of (**1**) (figs. S2 and S3) (*39*, *42*–*46*). Notably, we have shown that triketone functionalization of polyolefins can be performed in an industrially relevant twin-screw extruder, which circumvents the use of organic solvents (*39*, *42*–*46*). We systematically tuned the percent functionalization between 0.1 and 0.7 mol % for HDPE and 0.1 to 0.3 mol % for *i*PP by adjusting reagent stoichiometry. Percent functionalization (mol %) was determined with respect to the polymer repeat unit by high-temperature ^1^H NMR spectroscopy. By tuning the percent functionalization across this range, we gained access to semicrystalline, triketone-functionalized polyolefins with systematic tuning of the spacing between diketoenamine crosslinking sites.

We next blended triketone-functionalized HDPE (0.3 mol % triketone) and *i*PP (0.3 mol % triketone) in a 70:30 weight percent ratio, which is common in mixed polyolefin waste streams (*11*). We then synthesized dynamically crosslinked blend **B1** at 130°C by using *tris*(2-aminoethyl)amine (TREN) (Fig. 1A). As controls, we prepared an unmodified HDPE-*i*PP blend (**UB**) in the same weight percent ratio, as well as a noncrosslinked blend (**NB**) using *N,N*-dimethylaminoethyl amine, which introduces diketoenamines on both HDPE and *i*PP but does not form a covalent network. The insoluble (gel) fraction for **B1** was 92% when synthesizing the blend in solution and 74% when synthesizing the blend in a ball mill without the use of solvent, indicating a high degree of network connectivity and an abundance of stress transferring strands, especially given the low number of crosslinking sites (one to three per polymer chain on average). In contrast, the **UB** had a gel fraction of 0% (fig. S4). Informed by stress relaxation experiments, melt reprocessing demonstrated that **B1** could be thermoformed to useful parts, at 200°C for ~2 hours (fig. S5). The reprocessed **B1** exhibited tensile properties that were similar to **B1**, indicating that dynamic bond exchange facilitated effective healing behavior under thermal conditions.

To prepare polymers for mechanical testing, we compression molded films of **B1** at 1 ton and 200°C for 2 hours. We subjected the dynamically crosslinked blend **B1** to uniaxial tensile testing at a strain rate of 0.005 s^−1^, whereupon we found it to be a ductile and tough material with a yield strength of ~16 MPa and failure at >500% strain. Under the same conditions, the unmodified (**UB**) and noncrosslinked (**NB**) blend controls fractured at only ~19% strain—i.e., prohibitively brittle for traditional polyolefin applications. Thus, dynamic covalent crosslinking conferred to the blend ~40-times higher tensile toughness compared to the unmodified and noncrosslinked blend controls (Fig. 1B and fig. S6).

To elucidate the role of phase behavior on the observed mechanical properties, we evaluated the phase morphologies of the blends by TEM. We acquired TEM micrographs after cryosectioning compression-molded films in a direction perpendicular to the film surface and using a heavy-element (RuO_4_) stain for enhanced contrast (Fig. 1C). The dynamically crosslinked blend **B1** exhibited a distinguishing striated co-continuous phase morphology that resisted alteration, even after annealing at 200°C for 24 hours (Fig. 1C and figs. S7 to S9). To probe the directional alignment of the striated morphologies, we also evaluated cryosections of **B1** by slicing parallel to the film surface. The TEM images supported the initial assessment of anisotropic striations throughout the material (fig. S10). This behavior sharply contrasted with that of unmodified HDPE and *i*PP blends (**UB**), wherein TEM showed droplets of *i*PP (light contrast) dispersed within the HDPE majority phase (dark contrast) (Fig. 1C). The diameters of *i*PP droplets in **UB** were 2.5 *±* 2.5 μm, similar to previous studies (*11*, *25*). The noncrosslinked blend **NB**, which featured pendant diketoenamines that did not link polymer chains, also demonstrated a droplet-like morphology, albeit with substantially smaller and more irregular domain sizes compared to **UB**. This observation suggested that polar diketoenamine bonds lowered the interfacial tension between HDPE and *i*PP phases in the **NB** but notably did not improve the mechanical properties of the blends.

The persistence of co-continuous blend architectures from immiscible polymers is unusual; elsewhere, co-continuous polyolefin blend architectures are transient nonequilibrium morphologies generated during melt processing and are preserved only through rapid melt quenching (*47*, *48*). The formation and stability of co-continuous morphologies in immiscible polymer blends can be described by the expression for the capillary number (*C*_a_), *C*_a_ ∝ η_m_ $\dot{\gamma }$ σ^−1^, where η_m_ denotes the matrix viscosity, σ denotes the interfacial tension, and $\dot{\gamma }$ denotes the shear rate. *C*_a_ must be greater than one for co-continuous architectures to be stable (*47*). On the basis of this expression, even dilute quantities of dynamic crosslinks in the matrix phase (i.e., HDPE) could result in a sufficiently high viscosity to kinetically trap co-continuous architectures, with additional but less substantial contributions from the lower interfacial tension, as evidenced by comparing the striated morphologies in **B1** and the droplet-like morphologies in **NB**.

**Fig. 2.**
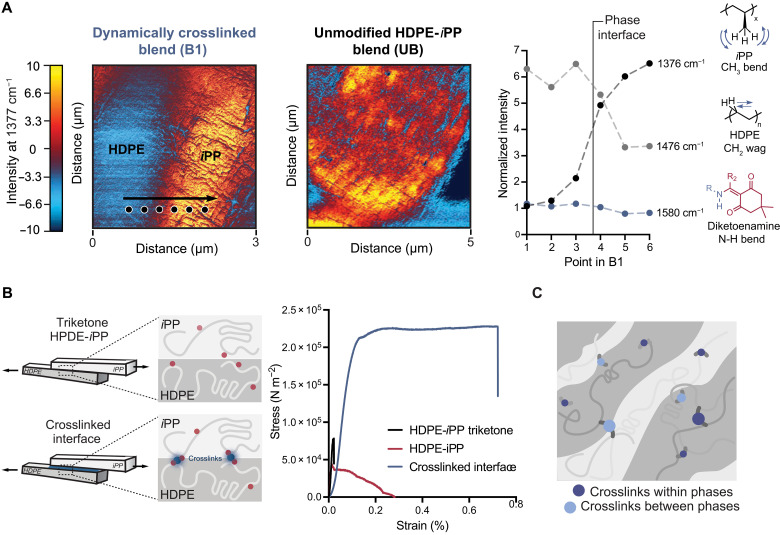
Dynamic covalent crosslinking in bulk and at interfaces produces co-continuous blend architectures and resultant properties. (**A**) Mapping of the vibrational modes from diketoenamine crosslinks, methyls (*i*PP), and methylenes (HDPE) with respect to phase structure by AFM-IR. (**B**) Lap shear tests of the HDPE-*i*PP interface for films crosslinked at the interface, (noncrosslinked) triketone HDPE-*i*PP, and HDPE-*i*PP. (**C**) Schematic representation of the role of dynamic crosslinks both in the bulk phases and at the phase interface, strengthening the interface and tuning bulk properties.

There are further implications of the prevalence of dynamic covalent crosslinks to the properties of the blends beyond morphology. Because immiscible polymer blends are known to fracture along polymer-polymer interfaces, crosslinks at the interface, if present, can contribute substantially to enhanced toughness by eliminating this common failure mechanism (*1*, *5*, *49*). Moreover, when dispersed throughout the phases and at the phase interface, we hypothesized that crosslinks would have the potential to contribute to enhanced stress transfer throughout the material, akin to an interpenetrating network. To characterize the location of diketoenamine crosslinks with respect to the phase structure, we carried out AFM-IR spectroscopy to map characteristic vibrational modes for HDPE, *i*PP, and diketoenamines by the detection of thermal expansion from the absorption of infrared (IR) radiation with a resolution of ~20 nm (*21*). Two distinct phases were identified on comparable length scales to those identified by TEM in both the dynamically crosslinked **B1** and **UB**. A frequency map was created for key wave numbers that were characteristic of polymer phase identity. Wave numbers corresponding to the methyl bending modes (1377 and 1457 cm^−1^) were observed at greater intensity in the *i*PP phase in comparison to the HDPE phase for both **UB** and **B1** (Fig. 2A); the wave number corresponding to the methylene wag vibrational mode (1476 cm^−1^) was identified at greater intensity in the HDPE phase for both **UB** and **B1** (figs. S11 and S12). The collection of IR point spectra, beginning in the HDPE phase and moving into the *i*PP phase for **B1**, showed decreasing intensity of methylene peaks and growing intensity of methyl peaks (Fig. 2A). To investigate the distribution of diketoenamine crosslinks throughout and between the phases, we used the characteristic N–H bend of the diketoenamine crosslinks at 1580 cm^−1^ (*36*). Vibrational modes corresponding to diketoenamine crosslinks were observed both throughout the bulk phases and at the interface at similar intensities in **B1**, whereas no such signal was observed for **UB** (Fig. 2A and figs. S11 and S12), indicating that diketoenamine crosslinks are located both in the HDPE and *i*PP phases and near the phase interface. Therefore, we find that the diketoenamine crosslinks contribute to a continuous covalent network of bonding both between and within phases. Because the AFM-IR for **B1** identifies diketoenamine crosslinks both within and between HDPE and *i*PP phases, the specific contribution of the interfacial crosslinks to the enhanced strength at phase interfaces could not be isolated by AFM-IR.

To assess the role of interfacial diketoenamine crosslinks on the interfacial adhesion between HDPE and *i*PP phases, we conducted lap shear tests of individually molded triketone-functionalized HDPE and *i*PP films that were crosslinked at their interface through the application of TREN as the amine crosslinker (Fig. 2B). Notably, substrates that were crosslinked fractured both at greater stress and in the bulk phase rather than at the interface. In comparison, the noncrosslinked films of the individually molded triketone HDPE–triketone *i*PP films and HDPE-*i*PP films fractured both at lower stress and at the HDPE-*i*PP interface. These data supported the contributions of dynamic crosslinks to the adhesion at otherwise weak HDPE-*i*PP interfaces. This insight is foundational, suggesting that the enhanced adhesion at the interface contributes to the enhanced mechanical properties of the dynamically crosslinked blends. Combining the insights from AFM-IR and lap shear data, a more holistic picture of the role of dynamic crosslinks began to emerge, playing a key role both at the phase interface and distributed throughout the bulk phases for effective stress transfer between chains (Fig. 2C). Yet, these structure-property insights benefit from additional context: specifically, how crosslinks across the interface and within the bulk alter polymer microstructure vis-à-vis crystallinity.

**Fig. 3.**
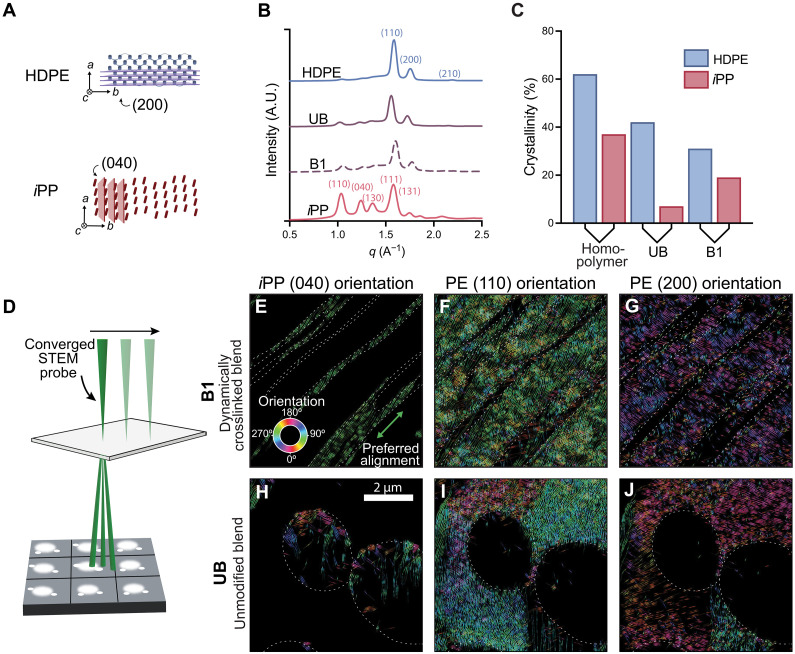
Dynamic covalent immiscible polymer blends orient crystallites parallel to phase interfaces. (**A**) Schematic of HDPE and *i*PP diffraction planes. (**B**) WAXS diffraction peaks corresponding to crystallites for HDPE, *i*PP, the unmodified blend (**UB**), and the dynamically crosslinked blend **B1** indicated that both polyethylene and polypropylene crystallites comprised the blends; a.u., arbitrary units. (**C**) Comparison of crystallinity fraction for the homopolymers, **UB**, and **B1** measured by DSC. (**D**) Schematic of 4D-STEM technique. (**E** to **G**) *i*PP and HDPE reflections map orientation and position of crystallites for **B1**. (**H** to **J**) Morphology and crystalline orientation of **UB** for comparison.

Crystalline lamellae serve as physical crosslinks in semicrystalline polymers and, therefore, play a key role in their mechanical properties. For polymer blends, the role of crystallinity is more complex. Crystallites contribute to connectivity within each individual phase, but cocrystallization of two different materials rarely occurs. To evaluate the impact of diketoenamine crosslinks and morphological changes on the crystallinity of each of the polyolefin phases, we evaluated blends for lattice plane spacings that correspond to the regular spacing between polymer backbones within polymer crystallites using wide-angle x-ray scattering (WAXS). Characteristic Bragg peaks for the *i*PP monoclinic phase and for the HDPE orthorhombic phase were observed in the dynamically crosslinked blend **B1**, indicating that both phases were semicrystalline with the characteristic packing of polymer chains for both HDPE and *i*PP (Fig. 3, A and B) (*50*). DSC provided further support by analysis of the melting transitions of the crystallites (Fig. 3C). A decrease in the melting enthalpy for HDPE indicated that the crystallinity of the HDPE phase decreased considerably from 62% for HDPE to 42% for the blended, unmodified polymers (**UB**), indicating that the blending alone disrupted crystallization. The percent crystallinity decreased to 35% for **B1**, where morphology and dynamic crosslinking play a combined role. In comparison to the HDPE phase, the *i*PP phase has a higher percentage crystallinity (37%) in crosslinked **B1** compared to the unmodified blend (7%), which we hypothesize is a result of the formation of a continuous *i*PP phase in **B1** in comparison to the droplet morphologies in **UB**.

To gain insight into the impact of covalent bonds and crystallinity on the dynamics of the blend, we leveraged the capabilities of ssNMR by measuring the spin-lattice relaxation in the rotating frame (*T*_1ρ_) to differentiate the characteristics of the crystalline, amorphous, and interfacial regions. Proton *T*_1ρ_ for amorphous domains is typically ~1 ms and longer for crystalline domains (*51*–*53*). With **UB**, we found the expected *T*_1ρ_ with a short time constant (1.5 ms), representing here amorphous regions in both HDPE and *i*PP phases. Concurrently, we observed an intermediate *T*_1ρ_ value (7.3 ms), likely representing interface regions often described as the rigid amorphous phase (fig. S13) (*53*–*55*), and a longer *T*_1ρ_ value (29 ms), likely representing crystalline regions. The **NB** exhibited similar T_1ρ_ values (fig. S14): 1.5, 6.7, and 28 ms, respectively. We compared these data with those for dynamically crosslinked **B1** (fig. S15), noting a decrease in each of the time constants: 1.2, 5.3, and 24 ms, respectively. We also noted a general broadening of all three peaks for **B1** as compared to **NB**. This suggests greater heterogeneity or blending across motional environments, potentially resulting from the percolated network of diketoenamine crosslinks that couple chain dynamics across phases.

To investigate the spatially resolved crystalline structure, in particular at the phase interfaces, we leveraged recent advances in 4D-STEM, hypothesizing that it could be used to map crystalline phase and orientation relative to the phase structure (Fig. 3D). With this approach, we acquired diffraction patterns with 16-nm resolution over an 8 μm–by–8 μm area, generating a 4D dataset with two spatial dimensions and two reciprocal space dimensions. The diffraction data reflected the expected *i*PP and HDPE lattice plane spacings (fig. S16) (*56*). The *i*PP phase was identified by its characteristic (040) set of planes with a corresponding d-spacing of 5.2 Å (spacing between *i*PP backbones), which were also observed in the WAXS data (Fig. 3, A and B, and table S1). Diffraction patterns in the HDPE phase showed the characteristic (110) set of planes (4.1-Å spacing) and the (200) set of planes (3.7-Å spacing) (spacing between HDPE backbones) (Fig. 3, A and B, and table S1). We used the vast diffraction data collected in these 4D-STEM scans to map the crystal structure of these specimens with high spatial resolution. Virtual bright-field images, formed by the integration of the central beam in postprocessing, showed the striated morphology of the dynamically crosslinked **B1** and the droplet morphology of **UB**, supporting the findings of the stained TEM images (fig. S16). We mapped the crystalline structure and types using the reflections identified above with a diffraction peak data analysis and flowline orientation mapping approach (Fig. 3, E to J) (*57*). Crystallite orientation was mapped by analyzing the rotation of diffraction reflections, with crystalline planes oriented perpendicular to Bragg spots. The color and direction of the lines indicate the orientation of crystalline planes (Fig. 3, E to J). The overlaid white dashed lines indicate the phase boundaries determined by principal component analysis (fig. S17), which removes spurious artifacts from bright-field images. From these data, it was apparent that **B1** exhibited strong preferred orientation parallel to the phase interface, especially for the *i*PP domains (Fig. 3E). The size of aligned crystalline domains was also smaller for **B1** compared to **UB** (fig. S18). These data indicated that diketoenamine crosslinks not only arrest co-continuous morphologies but also affect the orientation and organization of crystallites, further demonstrating the complex interplay between chemical structure, crosslinking, phase separation, and crystallization that combine to determine properties.

**Fig. 4.**
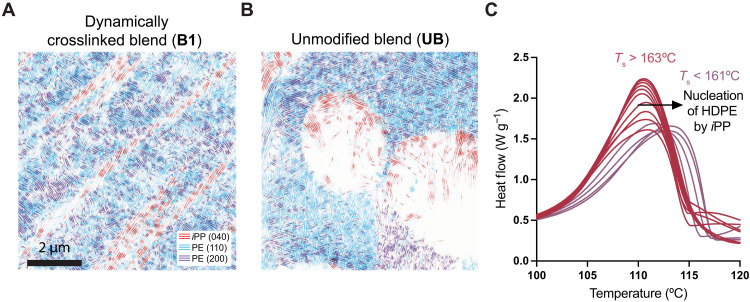
Dynamic covalent crosslinks template crystalline order in immiscible polymer blends. (**A**) Mapping of crystallite phase structure in the dynamically crosslinked blend (**B1**) shows a preferred directional alignment of crystallites especially at interfaces compared to (**B**) the unmodified blend (**UB**) with limited preferred alignment; (**C**) the shift to higher temperatures of HDPE crystallite nucleation in **B1** suggests the nucleation of HDPE crystallization by *i*PP, measured by DSC.

To investigate the orientation relationships between HDPE and *i*PP phases, we combined the maps of crystallinity for HDPE and *i*PP. We hypothesized that orientation relationships could lend insight into interfacial interactions between HDPE and *i*PP crystallites, which have been shown to play a role in material performance. The (040) reflections of the *i*PP phase were represented in red, and the (110) and (200) reflections of the HDPE were represented in blue and purple, respectively (Fig. 4, A and B). Notably, the (040) planes of the *i*PP phase were consistently aligned with the direction of the *i*PP striated phase morphology and consequently were parallel to the HDPE-*i*PP interface in **B1**. In contrast, the HDPE phase had a clear preferential orientation of the (110) reflections at a shallow angle relative to the interface and the (200) reflections with a perpendicular orientation with respect to the interface, although they were less consistently aligned in comparison to the *i*PP phase. The relative orientation relationship is especially noticeable near the interface; the preferred orientation persists throughout the field of view, suggesting that the diketoenamine crosslinks had templated the alignment of crystallites that presumably formed under shear conditions in the melt. In comparison, the unmodified blend, **UB**, exhibited some alignment of crystalline domains in the bulk HDPE phase but with diminished preferred orientation both in the bulk and at the interface. Like the HDPE phase for the **UB**, the *i*PP phase exhibited limited preferred orientation of crystallites. The *i*PP phase also displayed limited intensity of the crystalline peaks (040), especially in the center of the droplets, which we hypothesized may have resulted from more misorientation with respect to the electron beam (limiting diffraction), beam damage, lower percent crystallinity, or sample thickness (figs. S19 and S20).

We hypothesized that the preferential orientation of crystallite direction resulted from communication between crystalline phases during the nucleation process. To evaluate the role of dynamic crosslinks and multiple crystalline phases on crystallite nucleation, we conducted isothermal DSC experiments by annealing at self-nucleation temperatures (*T*_s_) to determine the temperature ranges of crystallite nucleation and growth (*58*). For the dynamically crosslinked polyolefin blend (**B1**), the onset of self-nucleation of the *i*PP crystalline phase at 161°C concomitantly induced nucleation of the HDPE phase, which is much higher than the nucleation temperature of the HDPE homopolymer (150°C) (Fig. 4C). Nucleation of HDPE was therefore initiated by the *i*PP crystallites in **B1**, suggesting that crystallization in the two phases are intimately connected despite macrophase separation, thus providing a mechanistic rationale for the orientation relationships that were observed in 4D-STEM. In **UB**, the onset of nucleation of the HDPE phase also increased compared to the HDPE homopolymer (166° versus 150°C), as described in previous work (*59*). We hypothesize that while annealing of the HDPE phase by the *i*PP phase is present in both **UB** and **B1**, shear-induced alignment of the *i*PP phase coupled with annealing behavior in **B1** collectively results in the orientation relationships that are observed.

Combining the aforementioned 4D-STEM analysis, we developed a model for the interfacial alignment of HDPE and *i*PP crystallites (fig. S21). The strong alignment of the (040) crystalline planes in *i*PP phase in **B1** determined that the *b* axis of the *i*PP crystalline domains were perpendicular to the interface. Consequently, the *i*PP polymer backbone is oriented parallel to the interface, although it is not possible to determine the alignment of the *a* and *c* axes as a result of the identification of only the (040) reflection at sufficient intensity in the *i*PP phase (table S1 and fig. S16) (*50*). In the HDPE domains, both the (110) and (200) reflections were mapped. We plotted the relative orientations between the (110) and (200) reflections for each diffraction pattern as a histogram to evaluate whether the reflections were from the same domain or from different domains through the thickness of the sample (fig. S22). When the angle between the (110) and (200) reflections is close to the theoretical 56°, the reflections were identified to be from the same grain. We uniquely determined the orientation of HDPE crystallites for **B1** by colocating these two reflections (Fig. 4C and fig. S23). The *b* axis was oriented perpendicular to the interface, and the *a* axis was aligned parallel to the interface, making the HDPE polymer backbone parallel to the interface (*60*). We hypothesized that the preferential alignment between crystalline phases in **B1** resulted from dynamic crosslinking enforcing more order or from the communication or templating of crystalline order between the HDPE and *i*PP phases (*61*, *62*).

**Fig. 5.**
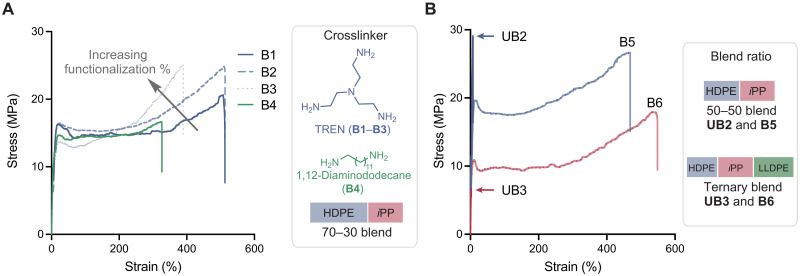
Understanding and controlling dynamic covalent immiscible polymer blend properties. (**A**) Stress-strain curves for HDPE-*i*PP blends **B1** to **B3** varying the percentage triketone functionalization or alternatively using 1,12-diaminododecane as a divalent crosslinker in HDPE-*i*PP blend **B4**. (**B**) Stress-strain curves for a 50:50 weight percent blend of HDPE:*i*PP (**B5**) and a ternary blend comprising 28:36:36 weight percent of HDPE, *i*PP, and LLDPE (**B6**) along with the corresponding unmodified blends (**UB2** and **UB3**, respectively).

Having established the relationship between bulk and interfacial properties of dynamically crosslinked HDPE-*i*PP blend **B1**, we further considered how the degree of functionalization and manner of crosslinking with different amines affected blend performance. To this end, we prepared an alternative dynamically crosslinked blend (**B2**) with more equivalents of amine crosslinker by reacting triketone-functionalized HDPE (0.3 mol % triketone) and *i*PP (0.3 mol % triketone) in a 70:30 weight percent ratio with a 1:1 ratio of TREN to triketone as well as blend **B3** by mixing triketone-functionalized HDPE (0.6 mol % triketone) and *i*PP (0.3 mol % triketone) in a 70:30 weight percent ratio with a 0.3:1 ratio of TREN to triketone. We also prepared dynamically crosslinked blend **B4** with a difunctional amine crosslinker (versus trifunctional TREN) by mixing triketone-functionalized HDPE (0.3 mol % triketone) and *i*PP (0.3 mol % triketone) in a 70:30 weight percent ratio and crosslinked the materials with 1,12-diaminododecane. We found a clear benefit to increasing the ratio of TREN to triketone from 0.3:1 in **B1** to 1:1 in **B2**, where blend **B2** showed a more consistent strain at break 517 ± 52 versus 422 ± 202 (Fig. 5A). We attribute more reproducible tensile strain and toughness data for **B2** versus **B1** to greater gel fraction in **B2** compared to **B1**, 92% versus 72%, which provides an interpenetrating network with more reproducible and effective stress transfer between and within phases. On the other hand, increasing the density of functionalization sites provided **B3** with a lower Young’s modulus compared to **B1** (304 ± 7 versus 256 ± 25%, *P* = 0.03) as a result of the lower percent crystallinity in **B3** compared to **B1**. With regard to the amine crosslinker, there were also clear benefits to blend mechanical properties by implementing trivalent crosslinkers, such as TREN in **B1**, over the divalent crosslinker 1,12-diaminododecane in **B4**; **B4** had the lowest strain at break for the series (289 ± 103%) as well as the lowest fracture stress (16 ± 2 MPa) and lowest overall tensile toughness (42 ± 15 MPa) (Fig. 5A). We then extended the approach to a 50:50 blend of HDPE and *i*PP that was prepared with 0.3 mol % functionalized HDPE and *i*PP using TREN at 130°C for 15 min. The dynamically crosslinked 50:50 blend **B5** exhibited ductile tensile behavior and reached 450% strain on average, whereas the unmodified 50:50 blend (**UB2**) was brittle, breaking at just 9% strain under uniaxial tensile testing (Fig. 5B). Notably, tuning the percent incorporation of *i*PP in the blend afforded tuning of the yield stress relative to corresponding 70:30 blend (20 ± 0.7 versus 15 ± 0.4 MPa, *P* = 0.003), thereby recovering one of the key benefits of polymer blending—the ability to tune thermomechanical properties by simply adjusting ratio of polymers in the blend. Last, we hypothesized that our method could be extended to complex ternary blends of polyolefins, which have limited prior approaches for compatibilization. HDPE, *i*PP, and, LLDPE were selected in a 28:36:36 percent ratio, reflecting the relative production volumes of these polymers. Ternary blend (**B6**) was prepared by crosslinking 0.3 mol % functionalized HDPE, 0.3 mol % functionalized *i*PP, and 0.1 mol % functionalized LLDPE using TREN at 130°C for 15 min. **B6** exhibited ductile tensile behavior and reached 550% strain on average, whereas the unmodified ternary blend (**UB3**) was brittle, breaking at just 3% strain under uniaxial tensile testing (Fig. 5B). Together, these mechanical data suggested that a dynamic reactive crosslinking strategy was effective at markedly improving the mechanical properties of polyolefin blends across a range of compositions and architectures. Moreover, the high degree of covalent connectivity (i.e., high gel fractions) of the blends aids in promoting efficient stress transfer, particularly at weak phase boundaries and interfaces, contributing to the improved mechanical properties.

## DISCUSSION

Overall, our results demonstrate a modular strategy to enhance the thermomechanical properties of commodity polyolefin blends, including ternary blends, that are challenging using other state-of-the-art block copolymer approaches. We demonstrate that commodity polyolefins can be used as starting materials for a dynamic crosslinking approach, wherein the high melt viscosity and enhanced interfacial adhesion yield unexpected, striated phase morphologies that fall outside of the bounds of expected morphologies for compatibilized blends. AFM-IR demonstrates that crosslinks are localized both at the bulk and at the interface, supporting their role both strengthening weak interfaces and modifying bulk properties. 4D-STEM analysis shows that crystallites in the polymer blend are aligned along the direction of shear, which points to a templating effect that dynamic covalent crosslinks exert during crystallization. Such high-resolution structural characterization across length scales reveals key factors that contribute to property outcomes, which we anticipate will provide structure-property insights that will inspire the development of compatibilization strategies to access valuable materials from immiscible polymer blends.

## MATERIALS AND METHODS

### Materials

Unless otherwise noted, solvents were dried and degassed using a Pure Process Technology solvent purification system. Other reagents whose syntheses are not described were purchased from commercial sources [Alfa Aesar (Ward Hill, MA), MilliporeSigma (St. Louis, MO), Oakwood Products (West Columbia, SC), Acros Organics (Geel, Belgium), and TCI America (Portland, OR)] and used without further purification. Chlorobenzene was distilled over CaH_2_ and was freeze pump thawed three times and stored in a glove box. All syntheses were performed under an inert N_2_ atmosphere using flame-dried or oven-dried glassware unless specified otherwise or in a N_2_-filled glove box. Thin-layer chromatography was performed on SiliaPlate 250-μm thick silica gel plates provided by Silicycle. Visualization was accomplished with short wave ultraviolet light (254 nm). Flash chromatography was performed using SiliaFlash P60 silica gel (40 to 63 μm) purchased from Silicycle. Compounds were isolated either by manual column or by Biotage Isolera flash chromatography.

### Methods

#### Sample preparation

Polyolefin was placed in a vial with distilled and degassed chlorobenzene under inert atmosphere in a glove box. The mixture was heated at 130°C for 5 min to solubilize the polymer. In a glove box, amide reagent and triketone trap were dissolved in chlorobenzene and added to the polymer-solvent mixture. The vial was sealed with electrical tape, removed from the glove box, and placed on a pie block to heat at 130°C and stir for 30 min. Functionalized polymer was precipitated into acetone and collected via vacuum filtration.

A 70:30 ratio of triketone-functionalized HDPE and triketone-functionalized *i*PP was placed in a vial with distilled and degassed chlorobenzene under inert atmosphere in a glove box. The mixture was heated at 130°C for 5 min to solubilize the polymer. In a glove box, multiamine and additional chlorobenzene were added to the polymer-solvent mixture. The vial was sealed with electrical tape, removed from the glove box, and stirred at room temperature for 2 min. The reaction was stirred and heated on a pie block at 130°C to react. The stir bar stopped stirring after 5 to 20 min as a result of gel formation. After the reaction was complete, the gel was removed from the vial and placed into a scintillation vial ¾-full of stirring acetone. The remaining chlorobenzene solution was added to the vial of stirring acetone. Crosslinked polymer was collected via vacuum filtration and dried overnight under vacuum before characterization.

#### Melt pressing

Polymer films of 150- to 250-μm thickness were prepared by melt pressing using a PHI Manual Compression Press. On a steel plate was placed a Kapton film (Kapton KN .01ʺ) (pretreated with Frekote 770-NC). A brass shim stencil of 200-μm thickness was placed to control film thickness before polymer was added. Another Kapton film was placed on top followed by a second steel plate. Crosslinked samples were pressed for 2 hours at 200°C at 1 ton. Linear samples were pressed for 5 min at 150°C at 1 ton. Films were removed from the melt press and cooled to room temperature by rapid heat transfer to an aluminum surface.

#### High-temperature gel permeation chromatography

High-temperature gel permeation chromatography (HT GPC) spectra were obtained using a Tosoh EcoSEC-HT GPC using TSKgel GMHHR-M columns. A solution of 1,2,4-trichlorobenzene (TCB) with 200 parts per million (ppm) of dibutylhydroxytoluene (BHT) was used as the mobile phase at a flow rate of 1 ml/min. The instrument was calibrated using polystyrene standards in the range of 580 to 5,480,000 Da. A calibration curve was created using refractive index detection against polystyrene standards (2 mg/ml) in TCB with 200 ppm of BHT at 140°C. A tandem multiangle light scattering detector could also be used on the HT GPC via a Wyatt DAWN 8 heated flow cell instrument.

#### Tensile testing

For tensile testing, melt pressed samples were cut into dog bones using an International Organization for Standardization 527 type 5B cutting die to standard dimensions (12-mm bridge length and 2-mm bridge width). Sample thickness at the bridge was measured using calipers. Test specimens were affixed to the Instron 5566 Universal Testing Machine with at 500 N load cell with a starting gap of 18 mm. Samples were pulled at 0.09 mm s^−1^ at room temperature in triplicate. Average values and SDs are reported.

#### Thermogravimetric analysis

Decomposition onset temperatures (*T*_d_) of precipitated and dried polymer samples were measured by thermal gravimetric analysis on a TA Instruments Q5000 Thermogravimetric Analyzer. Polymer samples were heated from ambient temperatures to 600°C at a heating rate of 10°C/min. Values of *T*_d_ (temperature at 5% weight loss) were obtained from weight % versus temperature (degrees Celsius) plots.

#### Differential scanning calorimetry

Melting transition temperature (*T*_m_) and glass transition temperature (*T*_g_) of precipitated and dried polymer samples were measured using DSC on a TA Instruments Discovery DSC. Unless specifically noted otherwise, values for *T*_m_ and *T*_g_ were obtained from a second heating scan after the thermal history was removed. Samples of 1 to 10 mg were heated 10°C/min between 25° and 160°C.

#### IR spectroscopy

IR spectra were obtained using a PerkinElmer Frontier Fourier transform infrared spectrometer under attenuated total reflection; 32 scans were conducted per sample.

#### Nuclear magnetic resonance

NMR spectra were recorded using a Bruker Neo 400 MHz or Bruker AVANCE III 600 MHz CryoProbe spectrometer. Chemical shifts δ (parts per million) are referenced to tetramethylsilane using the residual solvent as an internal standard (^1^H and ^13^C). For ^1^H NMR: CDCl_3_ = 7.26 ppm, C_2_D_2_Cl_4_ = 6.00. For ^13^C NMR: C_2_D_2_Cl_4_ = 73.78 ppm.

High-temperature NMR for polyolefin characterization was recorded on a Bruker 500 MHz spectrometer set to 110°C with ethylene glycol standard to quantify temperature of the NMR probe—roughly 116°C in all cases. Solvent resonance served as the internal standard (^1^H NMR: C_2_D_2_Cl_4_ at 6.00 ppm; ^13^C NMR: C_2_D_2_Cl_4_ at 73.78 ppm). Delay time was set to 5 s (d1 = 5). Thirty scans were required for functionalization levels of 0.5 mol % or lower.

Coupling constants (*J*) are expressed in hertz (Hz). ^1^H NMR data are reported as follows: chemical shift, multiplicity (s = singlet, d = doublet, t = triplet, q = quartet, m = multiplet, dd = doublet of doublets, dt = doublet of triplets, bs = broad singlet), coupling constants (in hertz), and integration.

#### Rheology

Melt pressed samples were cut into discs (diameter = 8 mm). Sample thickness was measured using calipers. Samples were measured in an TA Instruments HR30 rheometer equipped with a temperature probe and tested under ambient atmospheric conditions.

Preliminary frequency sweeps and stain sweeps were completed to identify the linear viscoelastic regime. Frequency sweeps were conducted at 160°C, which was above the melt temperature. Oscillatory strain of 2% was applied, and frequency was varied between 0.1 and 100 Hz.

Strain sweeps were conducted at 160°C. Frequency was held constant at 1 Hz, and strain was varied between 0.1 and 100% strain.

Stress relaxation was measured at 200°C, significantly above the melt temperature to target study of activation energy due to bond exchange rather than also probing the impacts of crystallinity. On the basis of identification of the linear viscoelastic regime in preliminary strain and frequency sweeps, a step strain of 2% was applied to the material which then relaxed stress over 8 hours, depending on temperature. A constant axial force was maintained throughout testing.

#### Cryoultramicrotomography

Melt pressed films were cryosectioned using a Leica Em FC7 cryoultramicrotome at −60°C. A small piece (~1 mm by 1 mm by 0.2 mm) of the film was affixed to a sample holder using cyanoacrylate super glue. Samples were prepared at 100-, 200-, and 330-nm thickness by cutting at 20 mm s^−1^. A diamond blade was used with an attached pool of 60:40 wt % dimethyl sulfoxide:H_2_O, a eutectic solution to facilitate efficient sample collection. Samples floated on the pool surface, collected using a hoop, placed onto a copper grid for TEM and 4D-STEM or a silicon wafer for AFM-IR, and dried under ambient conditions overnight.

#### Transmission electron microscopy

Cryosectioned samples were stained with a RuO_4_ stain for TEM. The contrast stain was prepared immediately before use by mixing 15 mg of RuCl_3_ with 1 ml of NaOCl solution (10 to 15% available chlorine). Samples were exposed to a chamber with the stain for 2 hours. Samples were left in the fume hood for 2 hours. Samples were loaded into the Thermo Fisher Scientific Talos F200X Transmission Electron Microscope and were studied at room temperature under high vacuum at an accelerating voltage of 200 kV. Images were analyzed using ImageJ software.

#### Atomic force microscopy

Samples were prepared by the cryosectioning of melt pressed films using a cryomicrotome. AFM-IR measurements were performed using a Bruker Anasys nanoIR3 and measured in tapping mode. The system uses a pulsed, tunable IR laser provided from a quantum cascade laser (QCL) and focuses it onto the AFM tip. Each AFM scan provides a topography and phase map of the specified area, and by varying the wave number of the incident IR laser, an IR intensity map will also be collected concurrently. By repeating scans at different wave numbers, a set of IR maps was obtained of the same area. Alternatively, by keeping the tip stationary and sweeping through the available spectral range of the QCL, individual IR spectra are obtained at different locations.

#### Lap shear testing

The 10 mm–by–20 mm strips for lap shear testing were prepared of either polyolefin or triketone-functionalized polyolefin by melt pressing films of ~200-μm thickness and cutting them into 10 mm–by–20 mm strips. For the preparation of the crosslinked sample, the 0.1 mol % triketone-functionalized films were soaked in 1 wt % TREN in PhCl solution at 75°C for 10 min. PhCl was allowed to evaporate for 30 min. For both HDPE-*i*PP and crosslinked HDPE-*i*PP, melt pressing the films together was conducted at 120°C at 2 tons for force for 5 min using a stencil brass shim of 400-μm thickness. Test specimens were affixed to an Instron 5566 Universal Testing Machine. Samples were pulled at a constant rate 0.16 mm/s for lap shear experiments. Force was normalized by interfacial area.

#### Wide angle x-ray scattering

WAXS measurements were collected on a Xeuss 3.0 (Xenocs, France) equipped with a D2+ MetalJet X-ray source (Ga Kα, 9.2 keV, λ = 1.3414 Å). Melt pressed polymer films were adhered to the solid sample holder, aligned perpendicular to the direction of the incident beam (transmission mode), measured for 10 min at a sample-to-detector distance of 47 following calibration with a AgBeh standard. 2D images of the scattering patterns were collected on a Eiger 2R 4M hybrid photon counting detector with a pixel dimension of 75 μm by 75 μm (Dectris, Switzerland). Azimuthal averages were reduced from the 2D WAXS images and plotted in the form of intensity versus scattering vector (*q*), where *q* = (4π sinθ)/λ, following background and sample thickness corrections applied in the XSACT software package (Xenocs, France). 1D data were analyzed to extract the percent crystallinity (χ_c_) using Eq. 1, where *A*_c_ is the area of the crystalline peaks and *A*_a_ is the area of the amorphous halo using fityk software to fit the reflections with Gaussian fits to determine the area

$$\chi_{c}=\frac{A_{c}}{A_{a}+A_{c}}$$ (1)

The Scherrer equation was used to estimate the broadening for the 110 peaks due to crystallite thickness variation according to Eq. 2

$$L_{110}=\frac{K\lambda }{\text{βcosθ}}$$ (2)

Here, *K* is taken as a shape factor taken to be 0.9, λ is the wavelength of the incident beam, β is the full width at half maximum, and θ is the Bragg angle. Instrumental broadening was accounted for and subtracted from crystalline standards.

#### Soxhlet extraction

The gel content was determined through Soxhlet extraction. A vitrimer sample of 50 to 75 mg was placed in a teabag and subsequently placed in the Soxhlet extraction apparatus in 20 ml of chlorobenzene. Soxhlet extraction was carried out at 160°C for 6 hours after finding that additional 6-hour periods did not significantly alter the gel fraction. The teabag was then removed from the apparatus, rinsed with acetone, and dried on vacuum overnight. Teabags were opened, and the remaining mass of network was massed. Gel content was calculated as the ratio of final mass to initial mass. Control experiments were conducted on 50 to 75 mg of the 70:30 wt % blend of HDPE and *i*PP (**UB**). The Soxhlet extractions resulted in gel fractions of 0.

#### ssNMR spectroscopy

^1^H echo 1D NMR spectra were acquired on a 16.4 T magnet (corresponding to a ^1^H Larmor frequency of 700 MHz), using a 3.2-mm Bruker triple channel ^1^H/^13^C/^15^N probe at a magic angle spinning (MAS) rate of 23 kHz. The *T*_1ρ_ relaxation time was measured with a standard spin-lock experiment (90° pulse immediately followed by a phase shifted variable-length spin-locking pulse) under two conditions. For the overall spectrum, *T*_1ρ_ was measured on a 16.4 T magnet with a radio frequency spin-lock field of 48 kHz. For a site-specific *T*_1ρ_ measurements at a selected resonance, experiments were conducted on a 9.4 T magnet (Bruker, NEO400), using a Bruker BioSpin spectrometer equipped with an Avance IV Neo console with a 3.2-mm double resonance HX MAS probe, which allows for longer spin-lock duration at higher spin-lock field strength. The measurements used RF spin-lock fields of 60 kHz with samples spun at a MAS rate of 20 kHz. All experiments were conducted at 25°C at the Pines Magnetic Resonance Center.

#### Four-dimensional scanning transmission electron microscopy

4D-STEM was acquired on unstained, ultramicrotomed samples. The data were collected on the TEAM I microscope at the Molecular Foundry, a modified FEI Titan double aberration-corrected microscope. The TEAM I was operated at 300 kV with a probe defined by a 0.25-mrad convergence angle, created with a custom 2-μm aperture (*63*). Data were acquired at room temperature with ~240 e^−^ A^−2^ on the Dectris Arina camera. Scans (1024 × 1024) were acquired to cover the 8 μm–by–8 μm field of view, but the 1024 × 1024 × 192 × 192 data were binned to 512 × 512 × 96 × 96 immediately upon loading to improve the signal-to-noise ratio. 4D-STEM data were analyzed using the open-source package py4SDSTEM (*64*).
