# Supplementary material for: Polyolefin blends with co-continuous architectures enabled by dynamic covalent crosslinking
Source: Sci Adv. 2026 May 15;12(20):eaee2328. doi: 10.1126/sciadv.aee2328 (PMC13178546; doi:10.1126/sciadv.aee2328)
Supplement: Supplementary file 1 — Supplementary Text Figs. S1 to S59 Tables S1 to S3 [file sciadv.aee2328_sm.pdf]

Science Advances
AAAS

Supplementary Materials for

Polyolefin blends with co-continuous architectures enabled by dynamic covalent crosslinking

Eliza K. Neidhart *et al.*

Corresponding author: Brett A. Helms, bahelms@lbl.gov; Frank A. Leibfarth, frankl@email.unc.edu

Sci. Adv. **12**, eaee2328 (2026)
DOI: 10.1126/sciadv.aee2328

This PDF file includes:

Supplementary Text
Figs. S1 to S59
Tables S1 to S3

I. Materials cont.

Polyolefin substrates used in this study (molar mass determination by comparing to polystyrene standards):

- LLDPE (DOW™ DNDA 1081NT) M_n = 21 kg/mol; $Đ$ = 3.8
- HDPE from (Exxon™ HD6719) M_n = 30 kg/mol, $Đ$ = 4.4
- *i*PP (Basell Profax 6301 12 MFR) M_n = 62 kg/mol, $Đ$ = 5.1
- Post-consumer polyethylene was obtained from Highcube® recycling M_n = 23 kg/mol, $Đ$ = 7.6

II. Figures

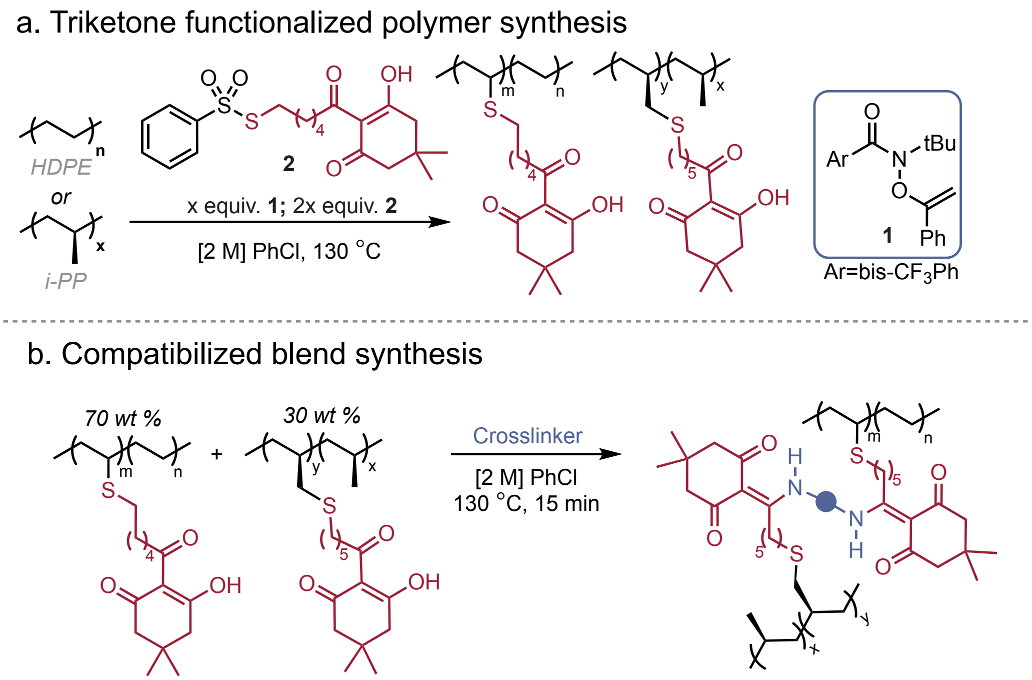

Figure S1: Preparation of dynamically crosslinked polyolefin blends.

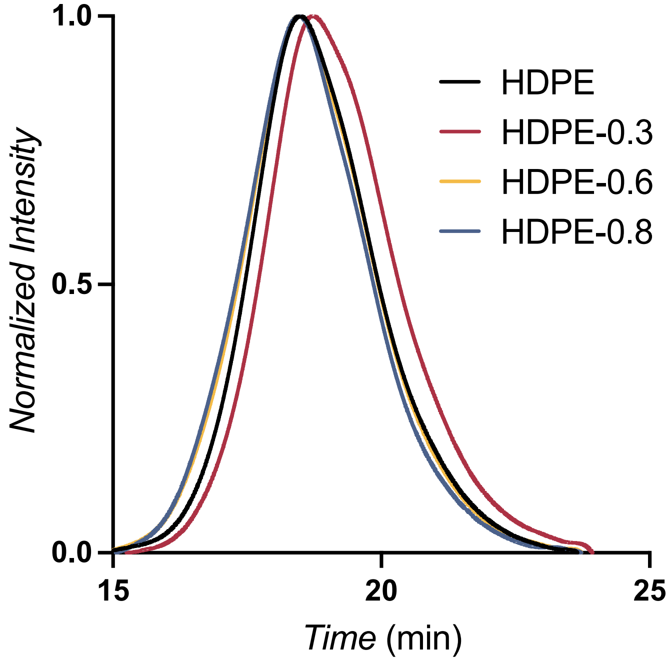

Figure S2: **Gel permeation chromatography of triketone functionalized HDPE**. (0.3 mol%, 0.6 mol% and 0.8 mol%) in comparison to HDPE. Triketone functionalized polymers had very similar retention times to the parent HDPE. Changes to *Đ* appear to be small indicating limited chain coupling or scission events.

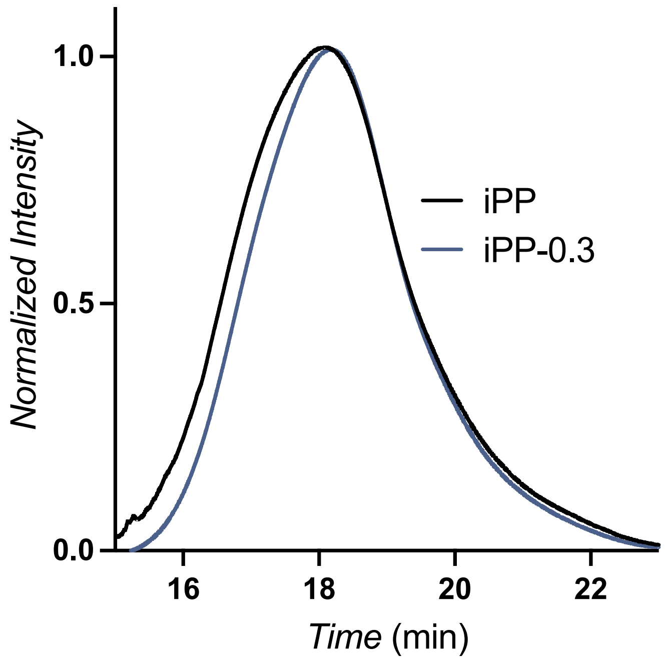

Figure S3: Gel permeation chromatography of triketone functionalized *i*PP. 0.3 mol% triketone functionalized *i*PP in comparison to *i*PP. Triketone functionalized polymers had very

similar retention times to the parent *i*PP. Changes to Đ appear to be small indicating limited chain coupling or scission events.

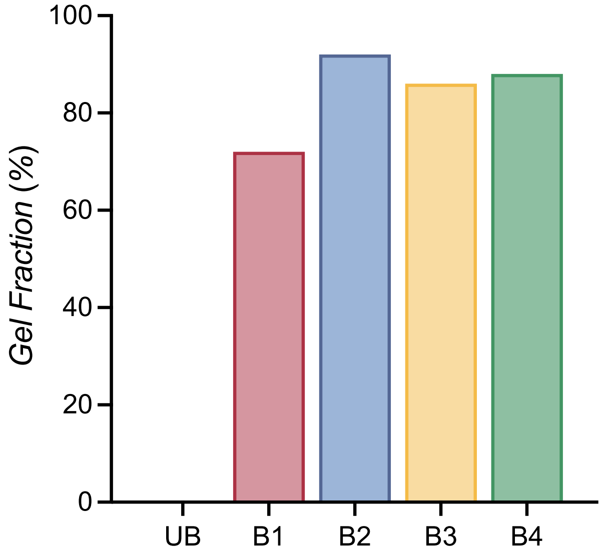

Figure S4: Gel fractions of polymer blends. Gel fraction indicates the insoluble fraction that could not be extracted from the covalently linked network in Soxhlet extraction experiments.

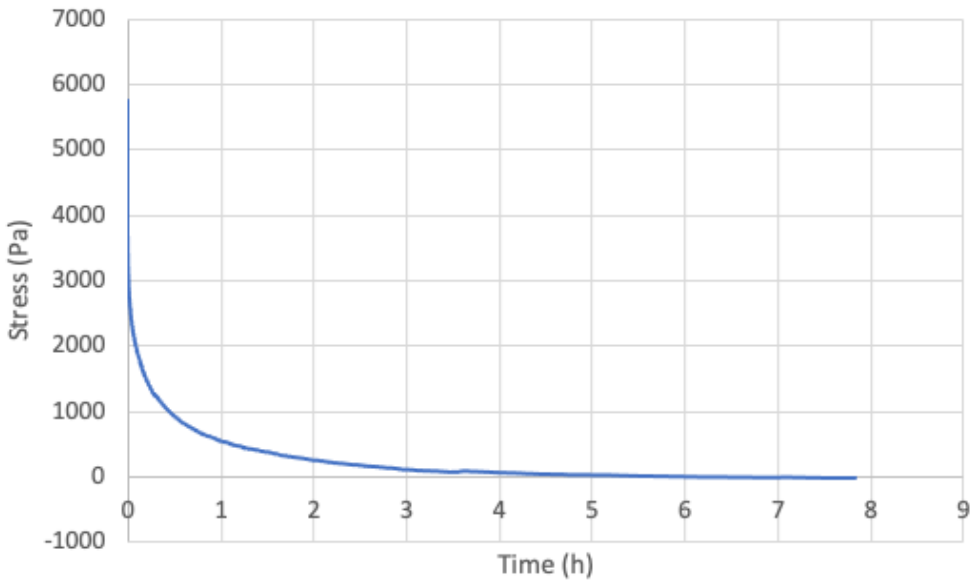

Figure S5: Stress relaxation experiments for B1. Conducted at 200 ºC, initial stress is dissipated over several hours, supporting dynamic bond exchange. An initial step strain of 2% was applied.

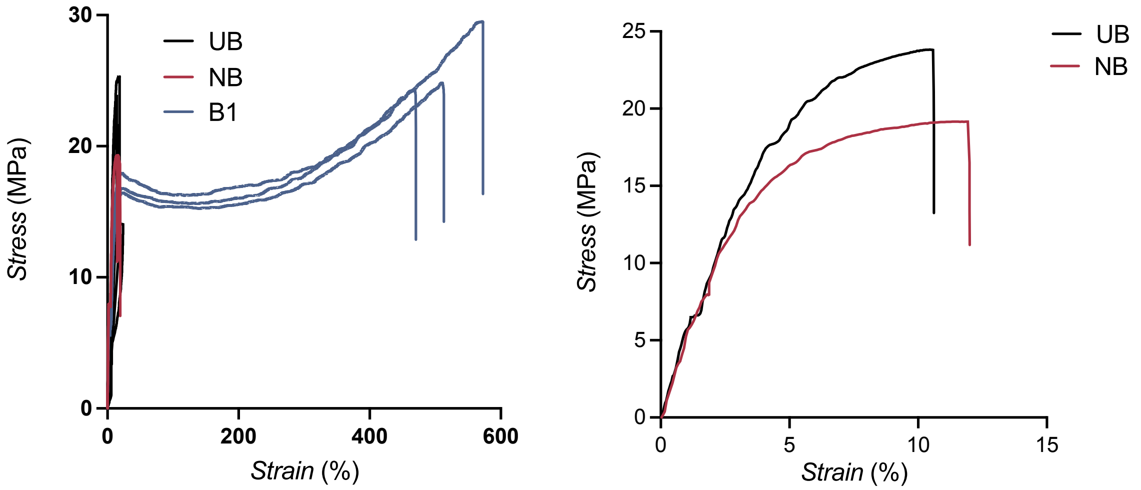

Figure S6: Tensile testing comparing UB, NB, and B1. Conducted at 0.09 mm s^{-1}. (right) inset of low strain regime.

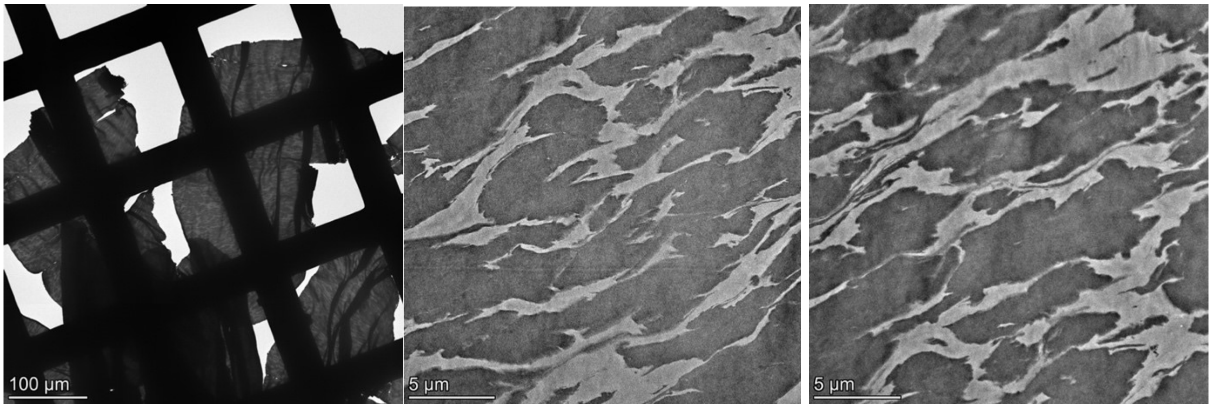

Figure S7: TEM of B1 after annealing. Annealing was conducted for 24 h in a glovebox under inert atmosphere, RuO_4 stain for enhanced contrast. Films were prepared perpendicular to the film surface.

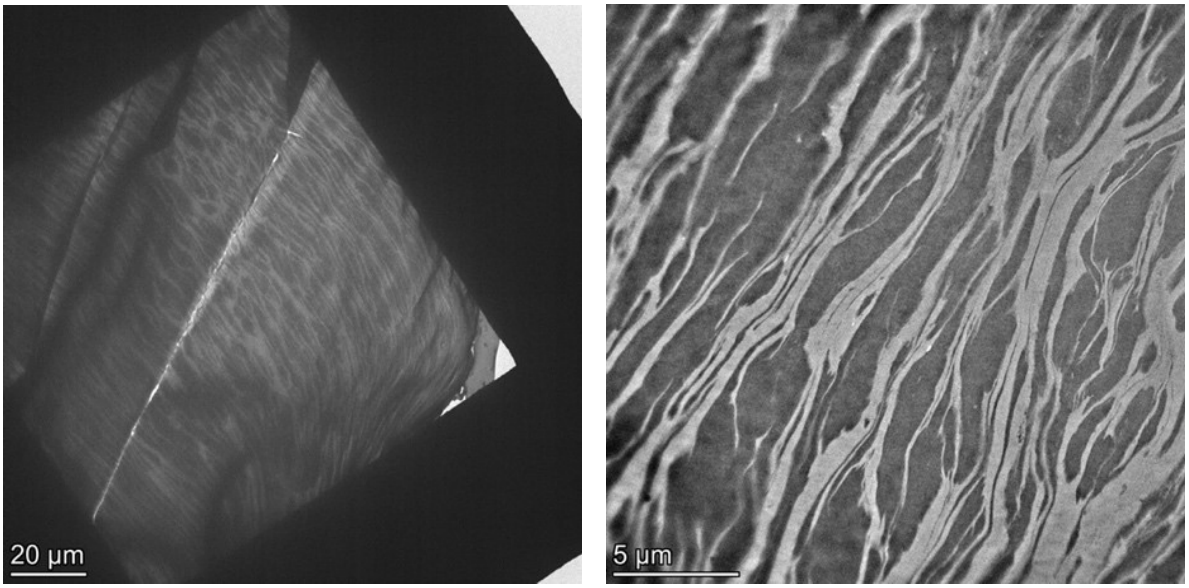

Figure S8: TEM of B1 after melt reprocessing. Films were cut into sections and melt pressing for 2 h, RuO_4 stain for enhanced contrast. Films were prepared perpendicular to the film surface.

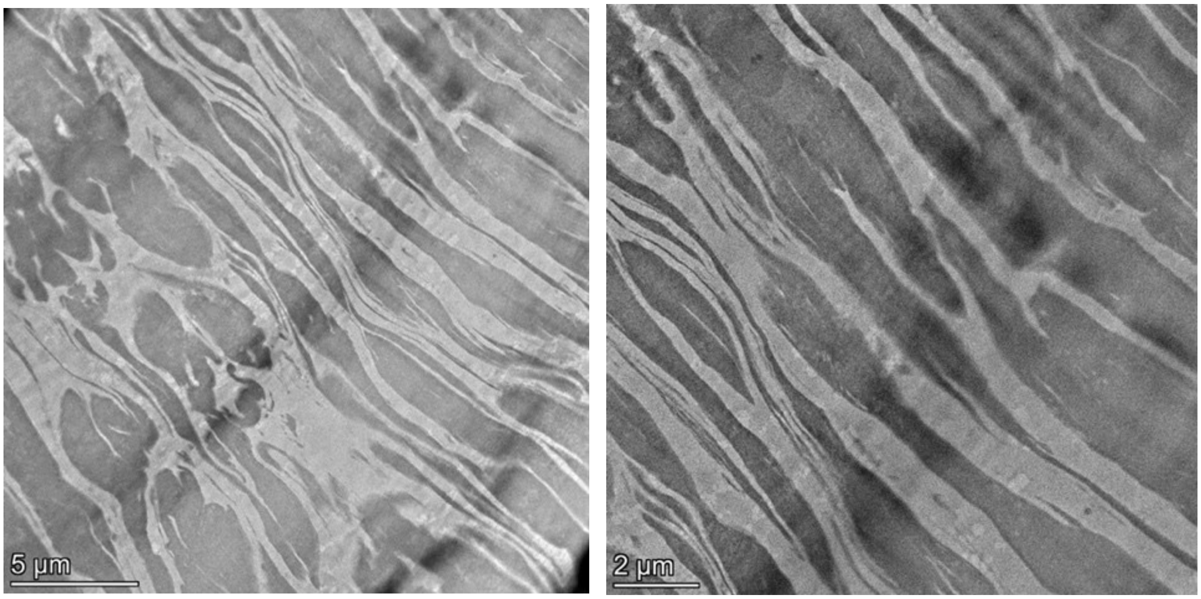

Figure S9: TEM of B1 after melt reprocessing. Films were cut into pieces and melt pressing for 3 hours, repeated 2 times. RuO_4 stain for enhanced contrast. Films were prepared perpendicular to the film surface.

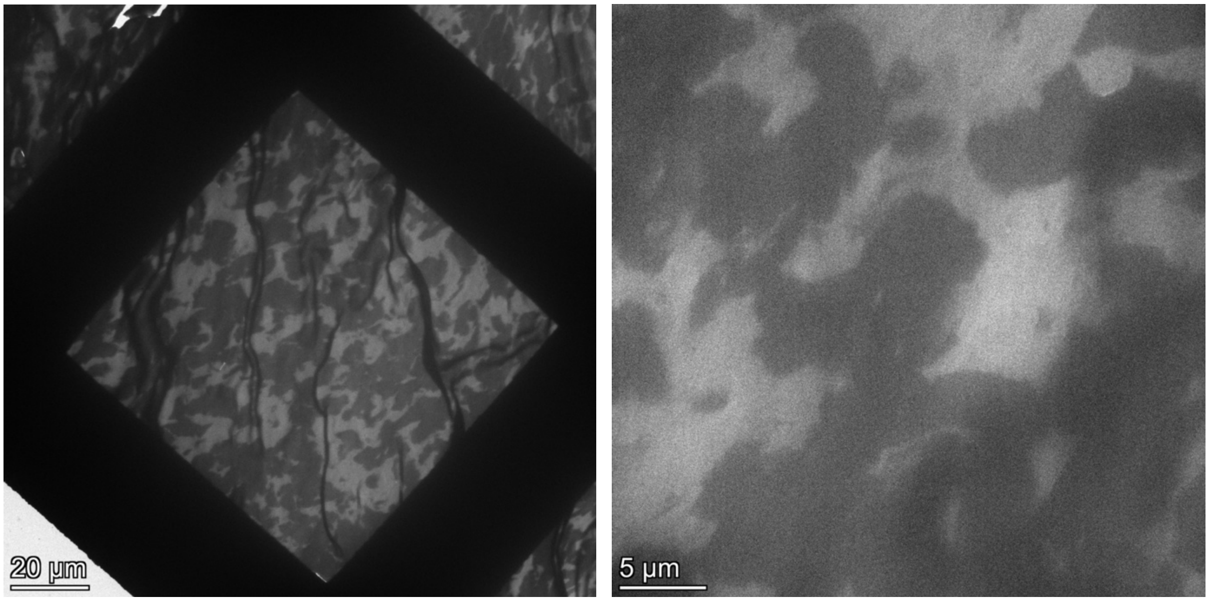

Figure S10: TEM of B1 in the direction *parallel* to the film surface. RuO_4 stain for enhanced contrast. All other samples are prepared perpendicular to the film surface.

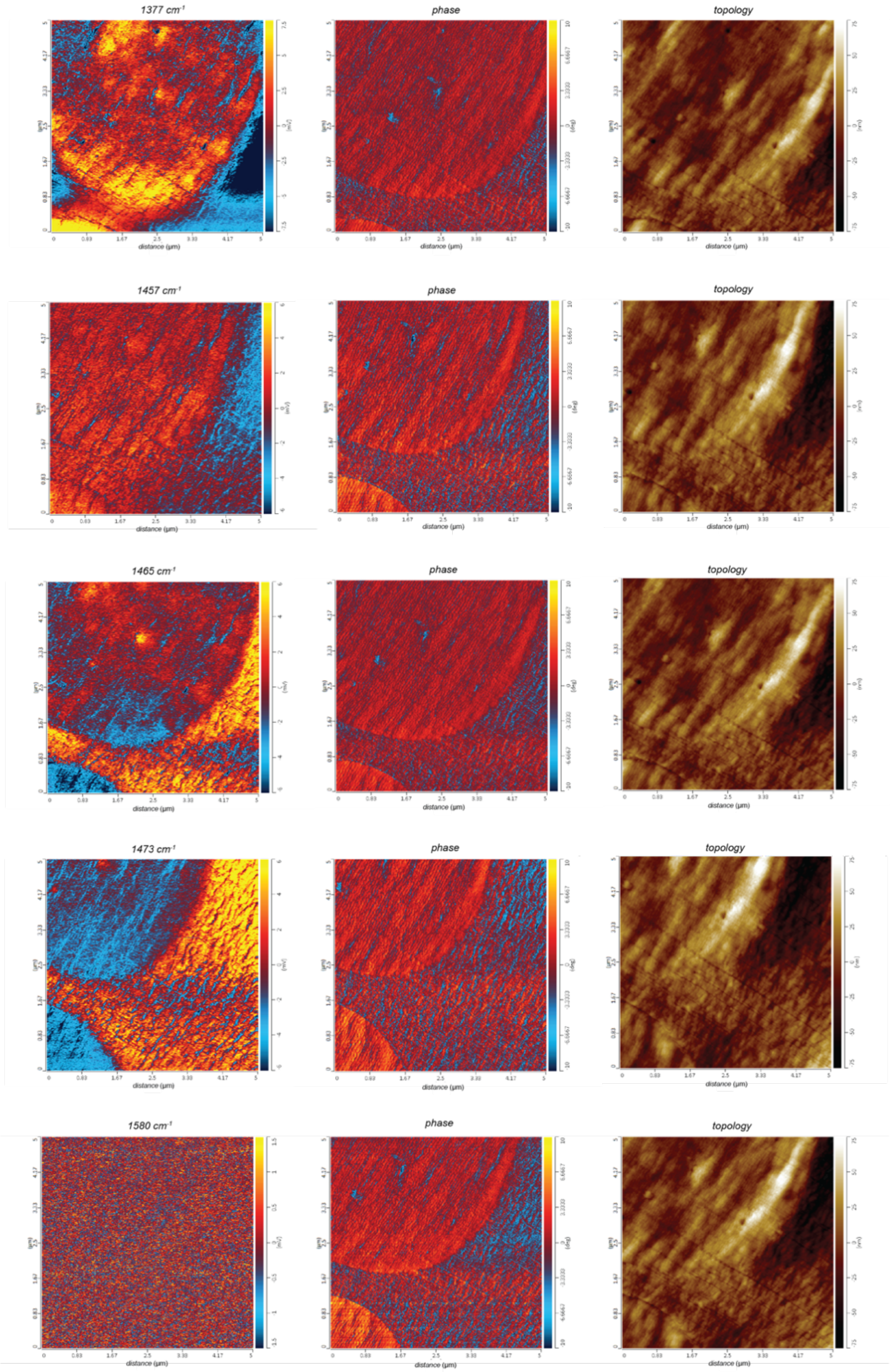

Figure S11: The topography, phase, and IR intensity maps for UB. IR maps at wavenumbers corresponding to the vibrational modes of polyolefins and diketoenamine crosslinks. Signal and contrast at 1580 cm^{-1} is negligible, indicating that diketoenamines are not present in **UB**. AFM-IR scans were taken at a 5 x 5 µm scan size and a 512 x 512 resolution.

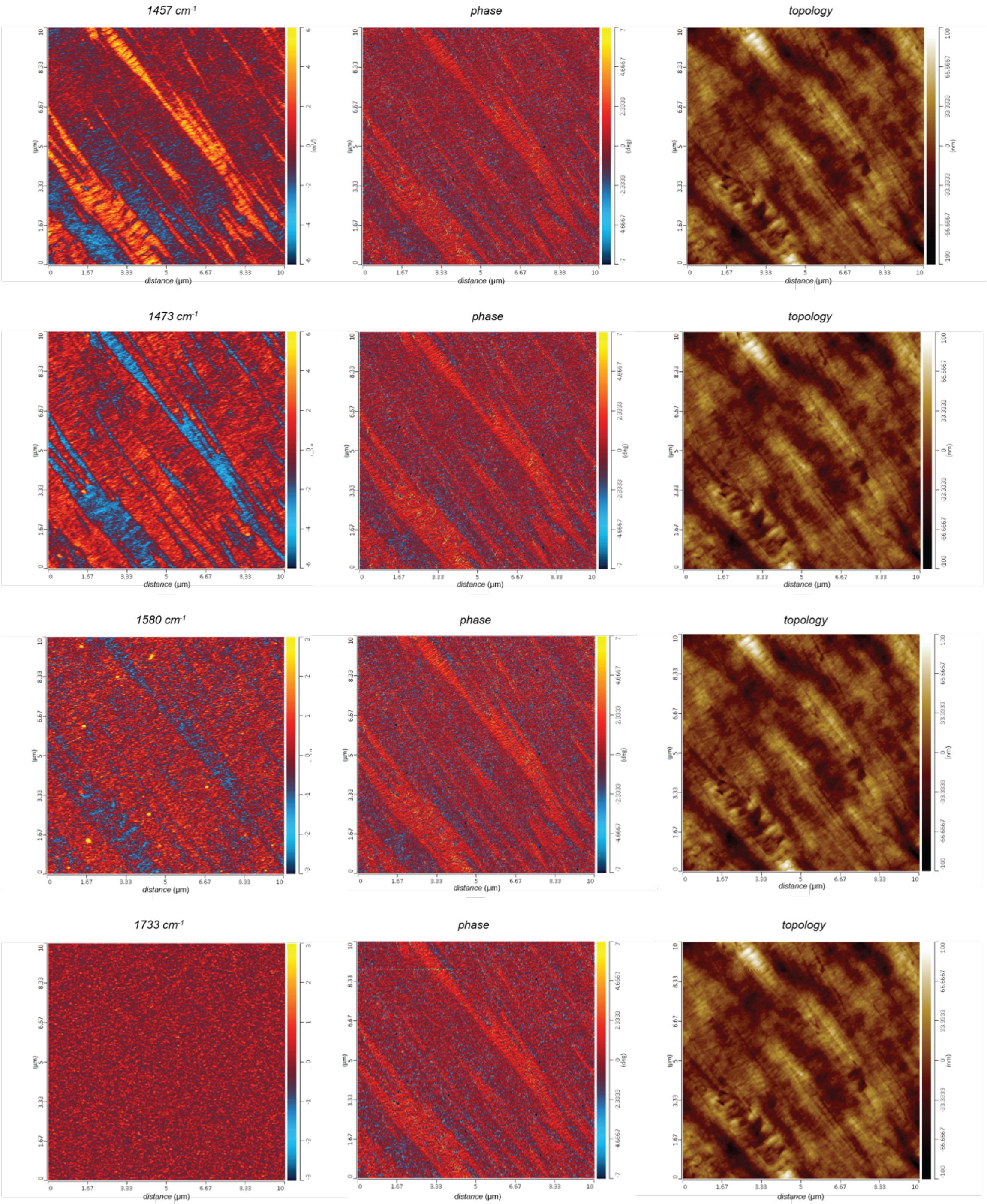

Figure S12: The topography, phase, and IR intensity maps of B1. IR maps at wavenumbers corresponding to the vibrational modes. Notably, the IR intensity map at 1580 cm^{-1} (corresponding to the diketoenamine N–H bend) exhibits a slightly greater intensity in the HDPE phase compared to the *i*PP phase. However, the difference between the two is small and the more important feature is that diketoenamines are present in both phases and near the phase interface. The peak at 1473 cm^{-1} originates from the methylene vibrational mode (greater intensity in the HDPE phase) while the peak at 1377 cm^{-1} and 1457 cm^{-1} originates form the methyl group (greater intensity in the *i*PP

phase). AFM-IR scans were taken at a 10 x 10 μm scan size and a 512 x 512 resolution. An IR intensity map at 1733 cm^{-1} was also taken as a control as no signal was expected.

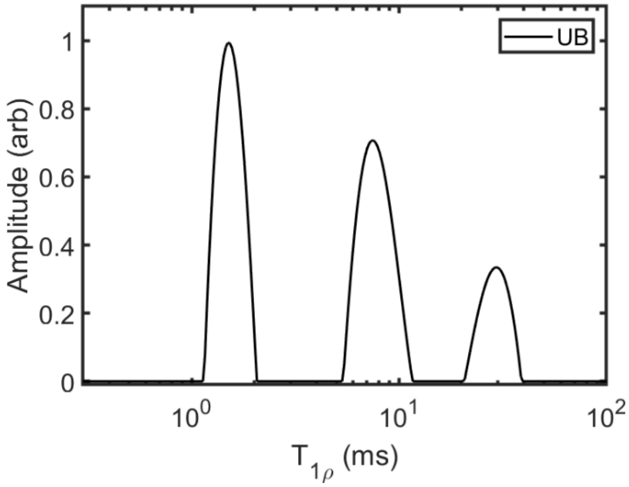

Figure S13: Solid state NMR T1ρ distribution of the unmodified blend (UB). Calculated via Laplace inversion. T1ρ refers to spin–lattice relaxation in the rotating frame.

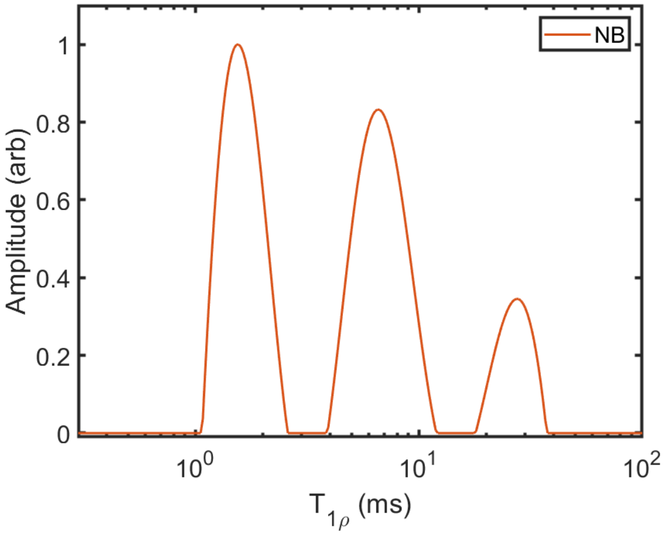

Figure S14: Solid state NMR T1ρ distribution of non crosslinked blend (NB). Calculated via Laplace inversion, exhibiting three distinct T1ρ values, as shown here (red) in a plot obtained via Laplace inversion. Here we see three distinct T1ρ values, with the peak ~2 ms representing the amorphous domains in both *i*PP and HDPE phases, an interfacial region(~7 ms), and a crystalline phase (~30 ms).

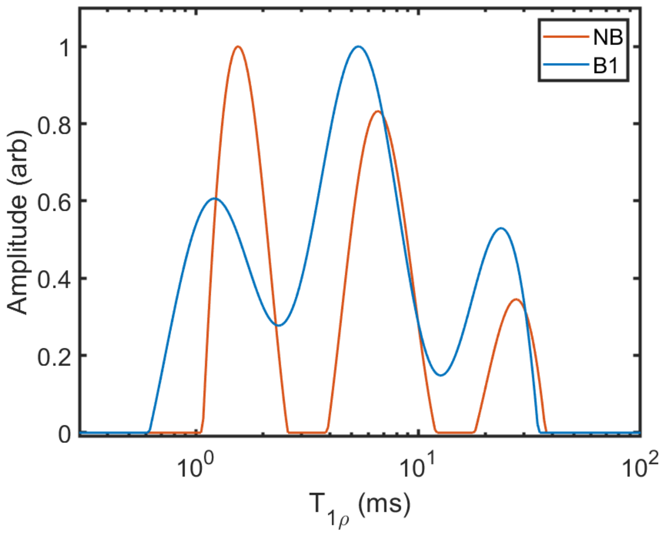

Figure S15: Solid state NMR T1ρ distributions of the of the entire ^{1}H spectra. B1 (blue, dynamically crosslinked) and **NB** (red, non-crosslinked blend) samples calculated via Laplace inversion.

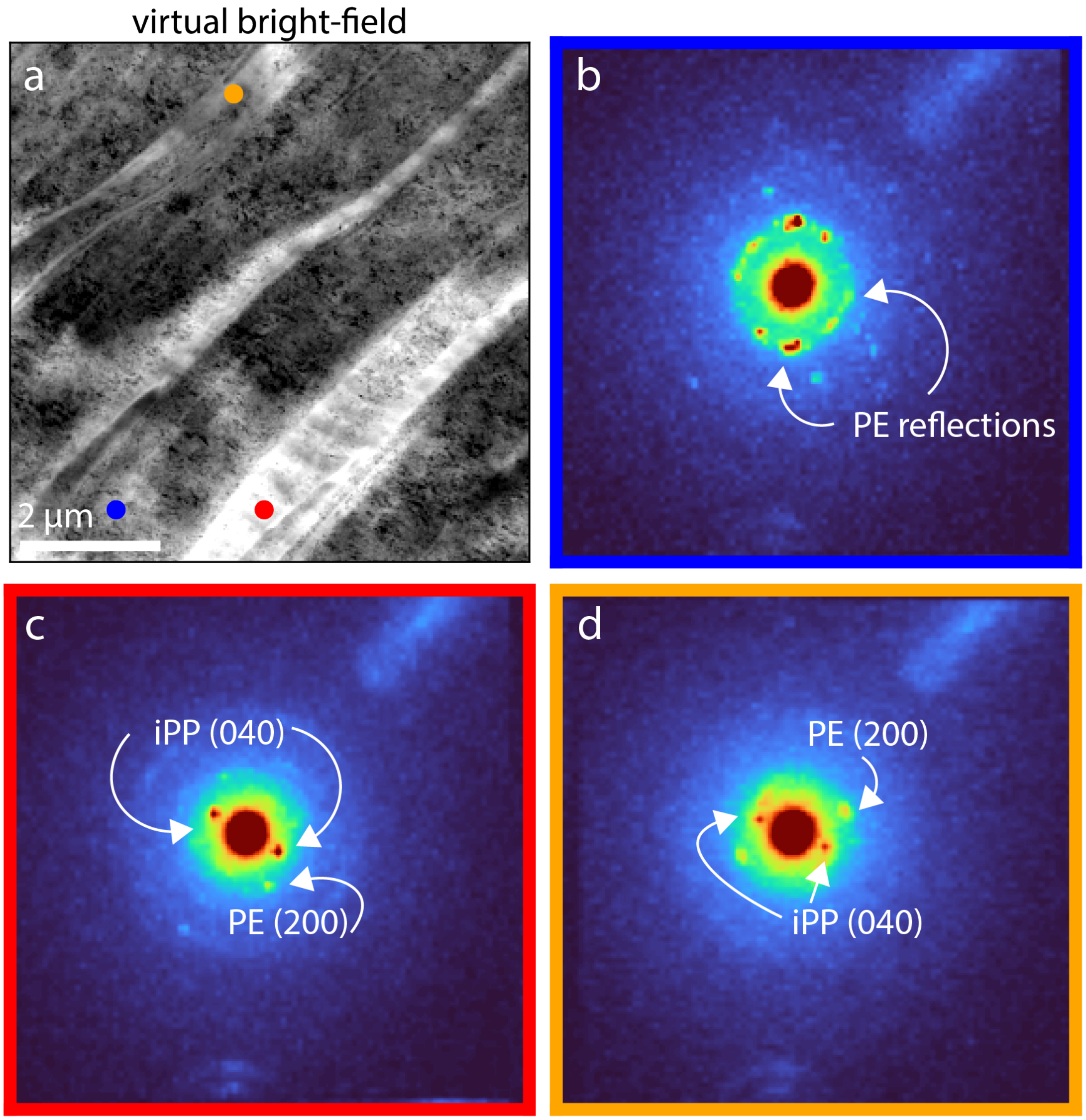

Figure S16: Example 4D-STEM diffraction patterns. Taken by taking the maximum across a 5 x 5 diffraction pattern region to more clearly highlight reflections from different phases.

*i*PP			HDPE		
Reflection	Spacing (Å)	Intensity (%)	Reflection	Spacing (Å)	Intensity (%)
(040)	5.2	100	(110)	4.1	100
(130)	4.8	64	(200)	3.7	100
(110)	6.2	64	(011)	2.3	52
(131)	4.1	60	(201)	2.1	26

Table S1: Intensities for electron diffraction for *i*PP and HDPE from 4D-STEM.

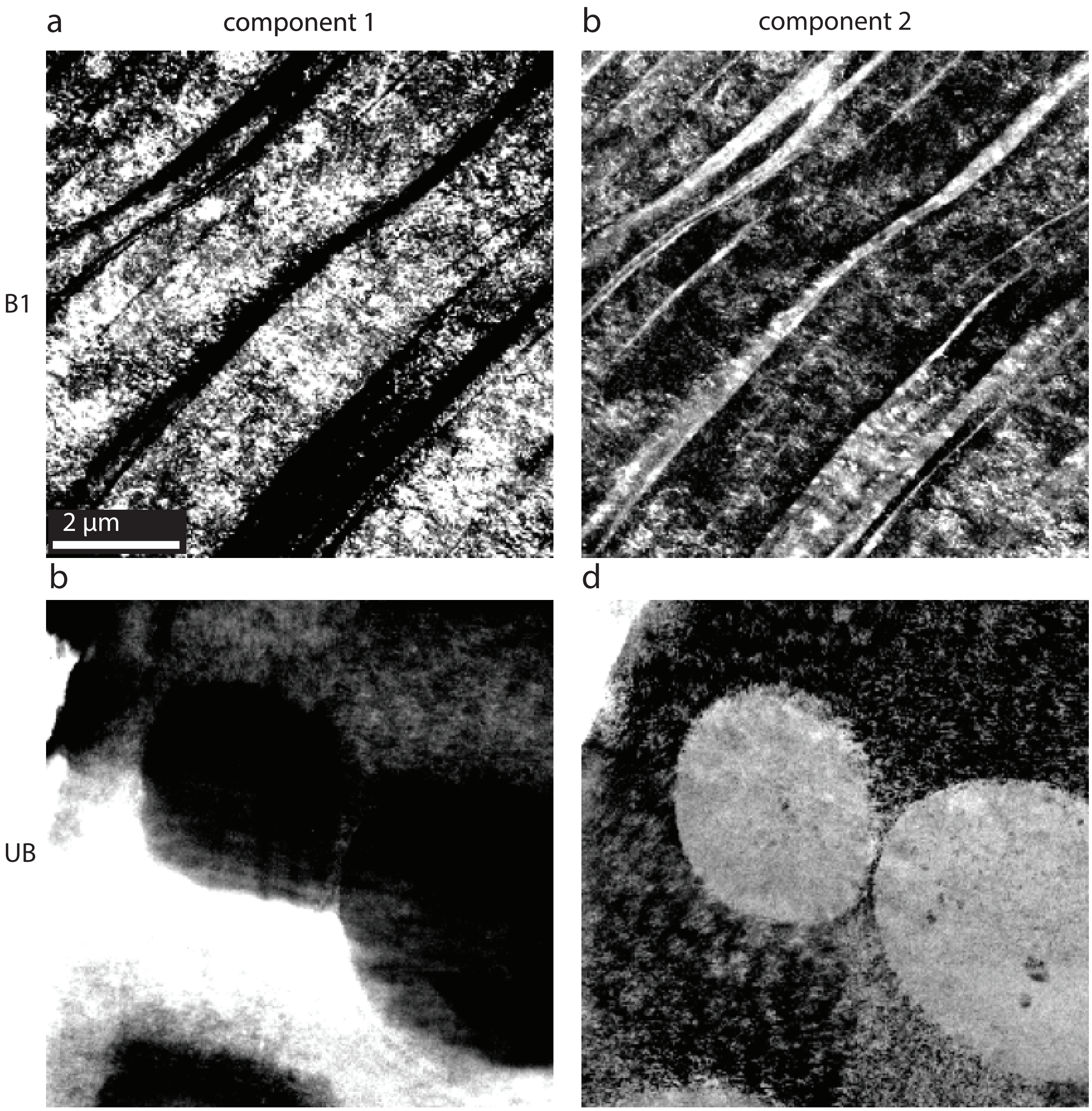

Figure S17: Principal component analysis of 4D-STEM data.

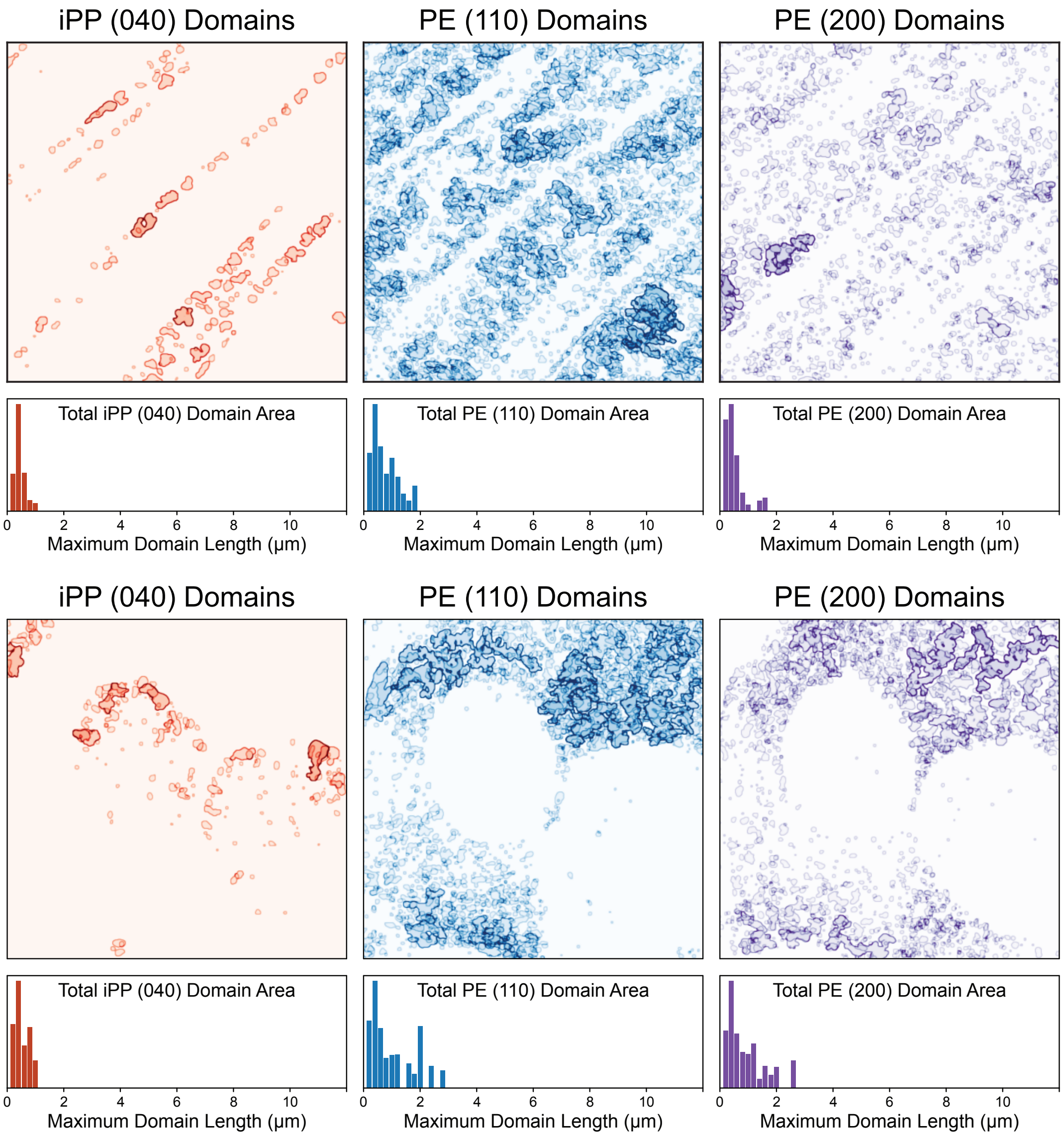

Figure S18: Domain size in B1 and UB. 4D-STEM phase maps. **B1** has smaller crystalline domains.

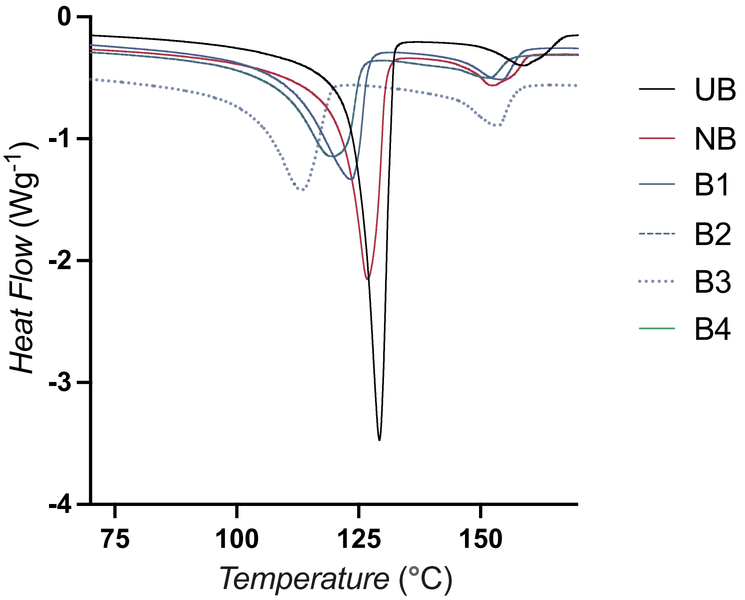

Figure S19: DSC data for UB, NB and the dynamically crosslinked blends (B1–B4). Reveals melting temperatures and enthalpies of melting of HDPE crystallites and *i*PP crystallites. Dynamic crosslinking results in a decrease in the melt temperature and enthalpy of melting. Data obtained from the 2nd heating cycle at a heating rate of 10 °C/min.

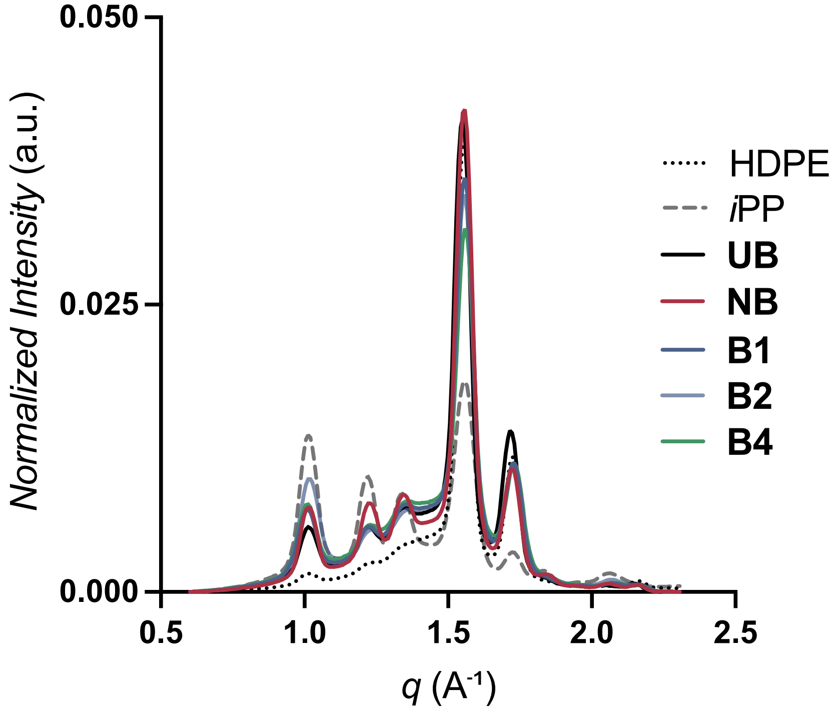

Figure S20: Wide angle X-ray scattering data for polyolefins and blends. *i*PP, HDPE, the unmodified blend (**UB**) and the dynamically crosslinked blends (**B1**, **B2**, and **B4**), non-crosslinked blend (**NB**).

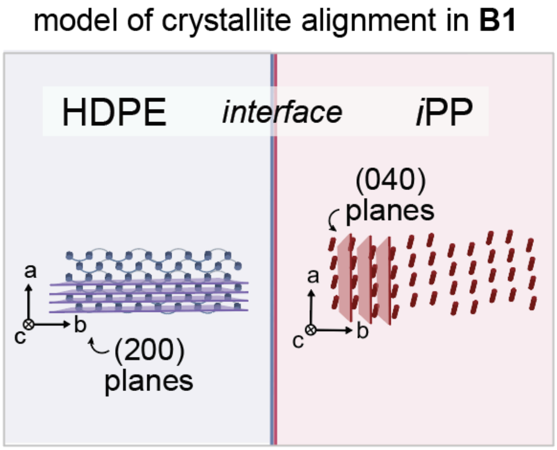

Figure S21: Model of the interfacial alignment of HDPE and *i*PP crystallites.

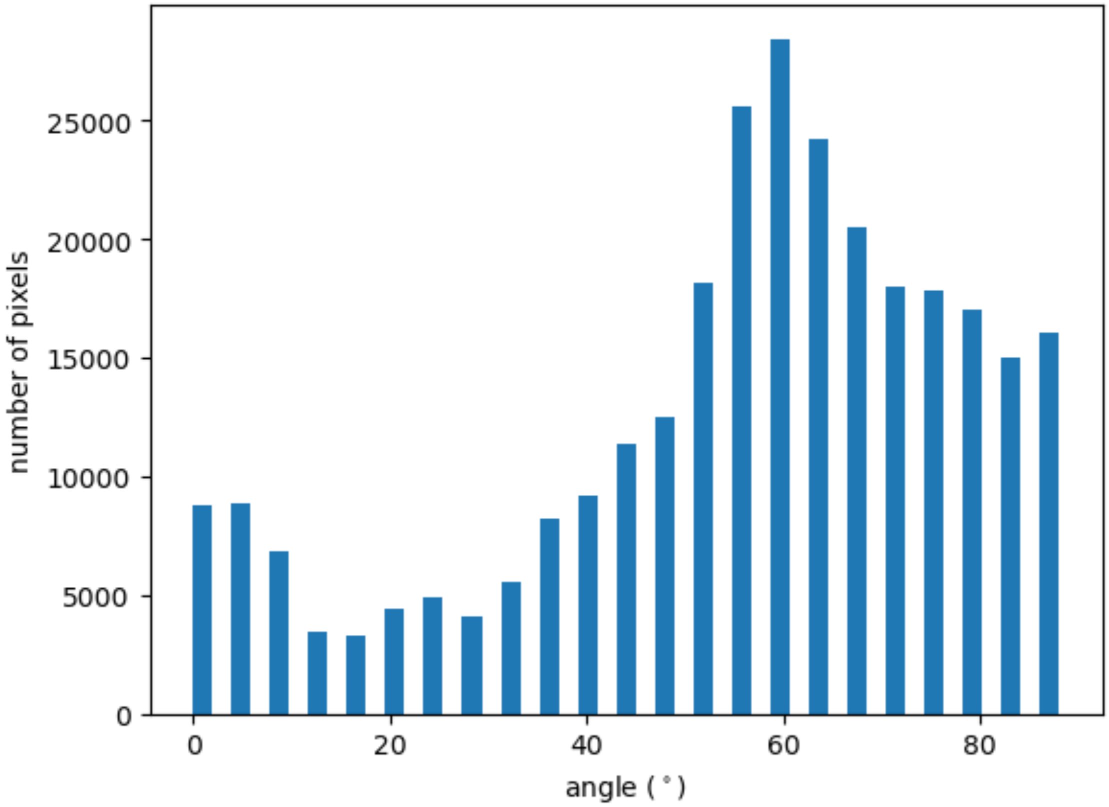

Figure S22: Relative orientation between (200) and (110) reflections in HDPE. 4D–STEM data.

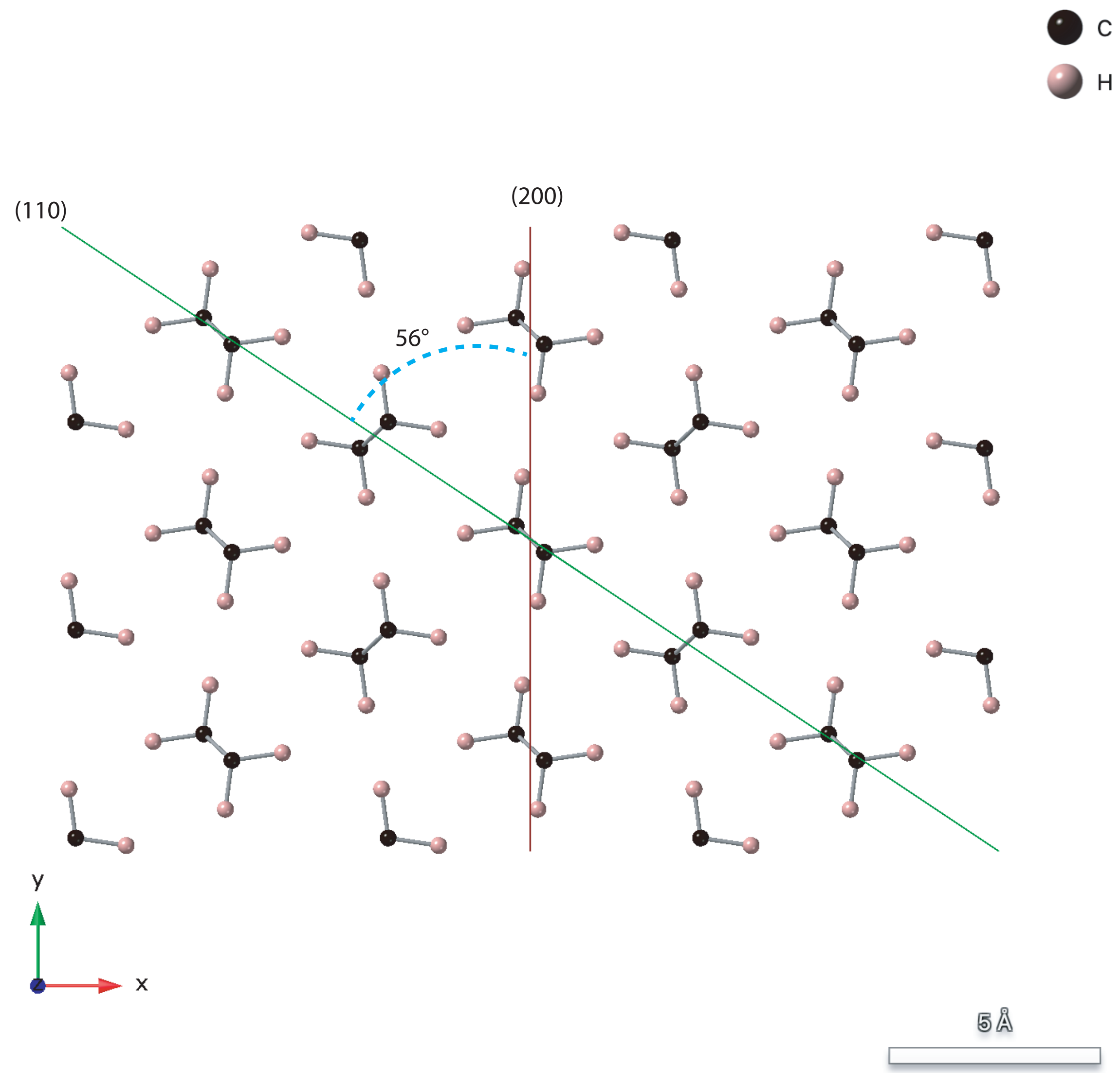

Figure S23: Rendering of diffraction planes with respect to HDPE chains. Orientation of (110) and (200) diffraction planes.

III. Small molecule synthesis

a. *O*-alkenylhydroxamate (amide reagent) synthesis

The amide reagent was prepared as described in reference [47].

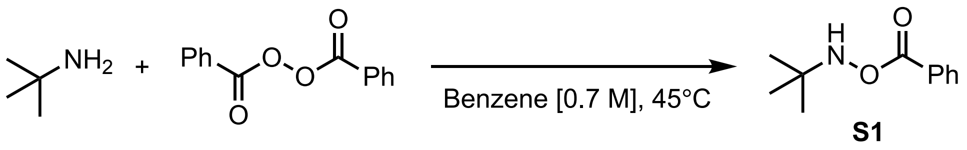

***N*-(*tert*-butyl)-*O*-benzoylhydroxylamine (S1):** To a flame dried round bottom flask with a stir bar was added benzoyl peroxide (20.0 g, 82.6 mmol, 1 equiv.), which was then dissolved in benzene (115 mL, 0.7 M). The reaction was sealed and placed under N_2. A portion of *tert*-butylamine (21 mL, 330 mmol, 4 equiv.) was added and the reaction heated at 45 °C for 1 hour, and changed color from cloudy white color to cloudy Carolina blue. After 1 hour, a second portion of *tert*-butylamine (13.7 mL (overall 34.7 mL, 330 mmol, 4 equiv.)) was added through the septum and the reaction was let to stir overnight. Then, the reaction cooled to room temperature before it was diluted with diethyl ether, and solid ammonium salt was filtered off. To the filtrate was added acidic aqueous $FeSO_4$ in ~1M H_2SO_4 (50 mL); the mixture was stirred for 10 minutes. The mixture was transferred to a separatory funnel, and the layers were separated. The organic layer was washed with saturated sodium bicarbonate solution (3 X 50 mL), water (1 X 50 mL), dried over $MgSO_4$, filtered and concentrated to afford product **S1** as orange/yellow oil (13.8 g, 87% yield).

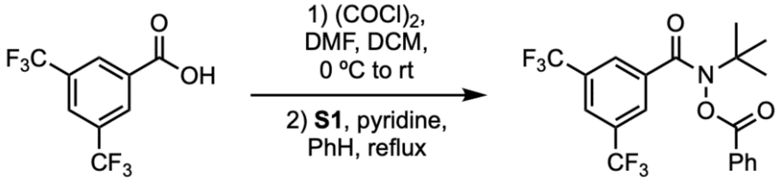

S2

***N*-(*tert*-butyl)-*O*-benzoyl-(3,5-bis-trifluoromethyl)-hydryoxyamide (S2):** To a flame dried round bottom flask with stir bar was added (3,5-bis-trifluoromethyl)-benzoic acid (16.8 g, 65.1 mmol, 1 equiv.), dissolved in dichloromethane (100 mL, [0.6 M]). Catalytic dimethylformamide was added (26 µL, 97 umol, 0.005 equiv.), and the reaction was sealed, and brought to 0°C. Oxalyl chloride was added dropwise via syringe (11.0 mL, 130 mmol, 2 equiv.), and the reaction stirred at 0°C for 15 minutes, then was let come to room temperature. The reaction was left to react until the cloudy white solution completely dissolved, forming a clear yellow solution, and continued to stir until bubbling subsided (~3 hours). Then, the reaction was carefully concentrated *in vacuo* to remove all excess oxalyl chloride, HCl, and CO; the resulting yellow oil was taken up in benzene (100 mL), and benzoylhydroxylamine (**S1**) was added (13.8 g, 71.6 mmol, 1.1 equiv.) in minimal amount of benzene (1-2 mL). Pyridine was added (11.1 mL, 137 mmol, 2.1 equiv.), the flask was equipped with a condenser, and brought to reflux overnight. At end of the reaction, the mixture allowed to cool to room temperature, diluted with diethyl ether, and pyridinium salt filtered off. Filtrate was transferred to separatory funnel, washed with 1 M hydrochloric acid (2 X 100 mL), water, dried over $MgSO_4$, filtered and concentrated to afford product **S2** as amber-colored solid (28.21 g, 99% yield). The compound was used directly in the next step without purification.

^{1}H NMR (600 MHz, $CDCl_3$) δ 8.03 (s, 1H), 7.82 (d, 2H), 7.76 (s, 1H), 7.59 (t, 1H), 7.41 (t, 2H), 1.62 (s, 9H),

^{13}C NMR (151 MHz, $CDCl_3$) δ 167.5, 165.2, 137.5, 134.6, 131.3 (q), 129.6, 128.8, 127.89, 125.7, 123.6, 121.9, 63.8, 27.5

^{19}F NMR (376 MHz, $CDCl_3$) δ –63.0

HRMS (HESI) Exact mass calcd for $C_{20}H_{17}F_6NO_3H$ $[M+H]^+$, 434.1191. Found 434.1180.

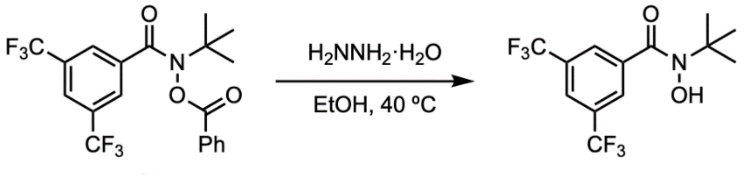

***N*-(*tert*-butyl)-(3,5-bis-trifluoromethyl)-hydryoxyamide (S3):** To a large round bottom flask with magnetic stir bar was added O-benzoylhydroxyamide **S2** (28.21 g, 65.10 mmol, 1 equiv.), dissolved in ethanol (180 mL, [0.3 M]). The reaction was capped and equipped with N_2 line. Hydrazine monohydrate (23.7 mL, 488 mmol, 7.5 equiv.) was added dropwise, and the reaction was heated to 40°C for 2 hours. The mixture was cooled to room temperature, then brought to 0°C. The mixture was then poured into ice water (~1.5 X volume of ethanol used), inducing precipitation of a white solid from yellow solution. The reaction was kept at 0°C for 5 minutes after which the solid was collected by filtration, washed with cold water, washed with pentanes, and dried thoroughly on hi-vac overnight to afford hydroxyamide product **S3** (18.1, 85% yield). The compound was used directly in the next step without further purification.

^{1}H NMR (600 MHz, $CDCl_3$) δ 8.00 (s, 2H), 7.96 (s, 1H), 6.68 (br s, 1H), 1.49 (s, 9H)

^{13}C NMR (151 MHz, $CDCl_3$) δ 167.6, 138.5, 131.6, 131.3, 128.1, 123.8, 123.6, 122.0, 62.2, 27.8

^{19}F NMR (376 MHz, $CDCl_3$) δ –62.9

HRMS (HESI) Exact mass calcd for $C_{13}H_{13}F_6NO_2H$ $[M+H]^+$, 330.0929. Found 330.0918.

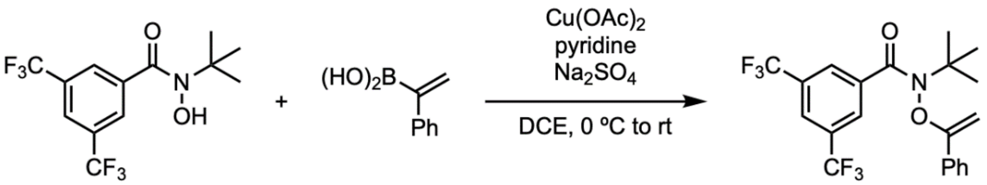

***N*-(*tert*-butyl)-*O*-(1-phenylvinyl)-(3,5-bis-trifluoromethyl)-hydryoxyamide (1):** To a large, dry round bottom flask with stir bar was added copper acetate (10.02 g, 55.2 mmol, 1 equiv.), sodium sulfate (31.3 g, 221 mmol, 4 equiv.), and hydroxamic acid **S3** (18.16 g, 55.2 mmol, 1 equiv.). Mixture left open to ambient atmosphere; 1,2-dichloroethane added (600 mL, [0.076 M]) to make a slurry, mixture cooled to 0°C. The flask was covered with aluminum foil, and hood lights turned off. Pyridine added (13.4 mL, 166 mmol, 3 equiv.), mixture let come to room temperature overnight. After 24 hours, 1,1-phenylvinylboronic acid (16.32 g, 110 mmol, 2 equiv.) was added, and the reaction was let stir at room temperature under ambient atmosphere, monitored by TLC until no more product being formed (~4 days). At end of reaction, the mixture was filtered through a pad of silica gel with dichloromethane to remove solid sodium sulfate and copper acetate. The filtrate was concentrated *in vacuo*, and further purified by silica gel column chromatography with 2% diethyl ether/hexanes (R_f ~ 0.5 in 5% Et_2O/hexanes) to give product **1** as yellow solid (15.05 g, 63% yield).

The product was stored in freezer in the dark, but was weighed out in the light on the benchtop for use in reactions.

^{1}H NMR (600 MHz, $CDCl_3$) δ 8.06 (s, 2H), 7.82 (s, 1H), 7.33 (m, 1H), 7.27 (m, 4H), 4.83 (d, 1H), 4.76 (d, 1H), 1.69 (s, 9H)

^{13}C NMR (151 MHz, $CDCl_3$) 169.4, 160.6, 138.2, 132.0, 131.3-130.6 (q, CF_3), 129.4, 128.3, 127.3 (d), 125.6-120.2 (q), 125.1, 123.3 (p), 87.3, 64.2, 27.5

^{19}F NMR (376 MHz, $CDCl_3$) δ –63.0

HRMS (HESI) Exact mass calcd for $C_{21}H_{19}F_6NO_2H$ $[M+H]^+$, 432.1398. Found 432.1387.

b. Triketone trap synthesis

Triketone was prepared as described in reference [40].

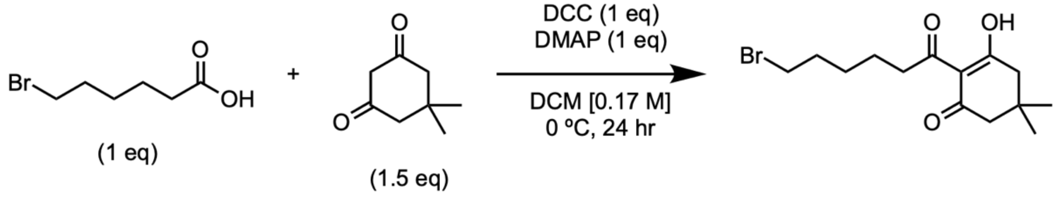

2-(6-bromohexanoyl)-5,5-dimethylcyclohexane-1,3-dione (S4): Bromohexanoic acid (1 eq, 200 mg, 1.03 mmol), dimedone (1.5 eq, 216 mg, 1.54 mmol), and DMAP (1 eq, 125 mg, 1.03 mmol) were added to a flame dried flask. Anhydrous dichloromethane (0.35 M in bromohexanoic acid, half of total reaction volume) was added via syringe to the round bottom under inert atmosphere. The reaction mixture was cooled to 0 ºC. A solution of DCC (1 eq, 212 mg, 1.03 mmol) was prepared in DCM (0.35 M, half of total reaction volume). The DCC solution was added dropwise to the cooled reaction mixture over 45 minutes. The reaction was warmed to room temperature overnight. After the reaction was complete, precipitate was filtered off and washed with DCM. The filtrate was collected and combined with 2 M aqueous HCl in a separatory funnel. The aqueous phase was extracted 2x into DCM. Combined organic layers were washed with H_2O. Finally, the organic layer was dried with $MgSO_4$ and concentrated via rotoevaporation. The product was isolated via flash column chromatography in EtOAc and hexanes from 0 to 10% EtOAc over 12 column volumes. Product was isolated in 72% yield. The reaction was scaled to 5 g of hexanoic acid with comparable yields.

^{1}H NMR (400 MHz, $CDCl_3$) δ 3.44 (t, 2H), 3.07 (m, 2H), 2.56 (s, 2H), 2.38 (s, 2H), 1.92 (dt, 2H), 1.67 (m, 2H), 1.55 (m, 2H), 1.10 (s, 6H)

^{13}C NMR (151 MHz, $CDCl_3$) δ 205.2, 197.7, 195.2, 112.0, 52.6, 46.8, 40.1, 33.6, 32.5, 30.7, 28.2, 27,8. 23.7

HRMS Exact mass calcd for $C_{14}H_{21}BrO_3$ $[M+Na]^+$, 339.06. Found 339.06.

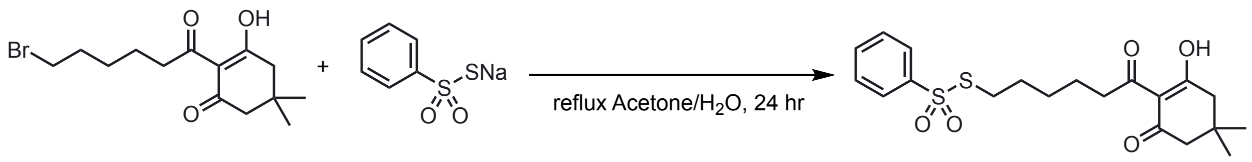

***S*-(6-(2-hydroxy-4,4-dimethyl-6-oxocyclohex-1-en-1-yl)-6-oxohexyl) benzenesulfonothioate (2):** To a solution of sodium thiosulfonate (1 eq, 1.5 g, 0.79 mmol) in Acetone:H_2O 97:3 (0.3 M) is added 2-(6-bromohexanoyl)-5,5-dimethylcyclohexane-1,3-dione (1 eq, 2.5 g, 0.79 mmol). Water was included to improve the solubility of the sodium thiosulfonate salt. The reaction mixture is refluxed overnight. After the reaction was complete, the mixture was diluted with H_2O and extracted 2x into EtOAc. The organic phase is dried with $MgSO_4$ and concentrated via rotoevaporation. The product was isolated via flash column chromatography in EtOAc and hexanes from 0 to 20% EtOAc over 12 column volumes. Product was isolated in 69% yield. The reaction was scaled to 6 g with comparable yields.

^{1}H NMR (400 MHz, $CDCl_3$) δ 7.95 (m, 2H),7.60 (m, 3H), 3.02 (t, 2H), 3.00 (t, 2H), 2.55 (s, 2H), 2.36 (s, 2H), 1.54 (m, 6H), 1.09 (m, 6H).

^{13}C NMR (151 MHz, $CDCl_3$) δ 205.1, 197.7, 195.2, 144.9, 133.6, 129.3, 127.0, 11.9, 52.6, 46.8, 40.0, 35.9, 30.7, 28.4, 28.2, 28.2, 23.8

FT-IR (neat, ATR, cm^{-1}) 2955, 2867, 1654, 1553, 1448, 1433, 1404, 1315, 1295, 1285, 1285, 1235, 1195, 1175, 1137, 1078, 1078, 1020, 997, 948, 922, 885, 835, 819, 763, 748, 735, 718, 696, 684, 599, 548, 460

HRMS Exact mass calcd for $C_{20}H_{23}O_5S_2$ $[M+Na]^+$, 434.11. Found 433.11.

III. Polymer Functionalization and Synthesis

a. Polymer functionalization

Functionalization of Polyolefins with Triketone

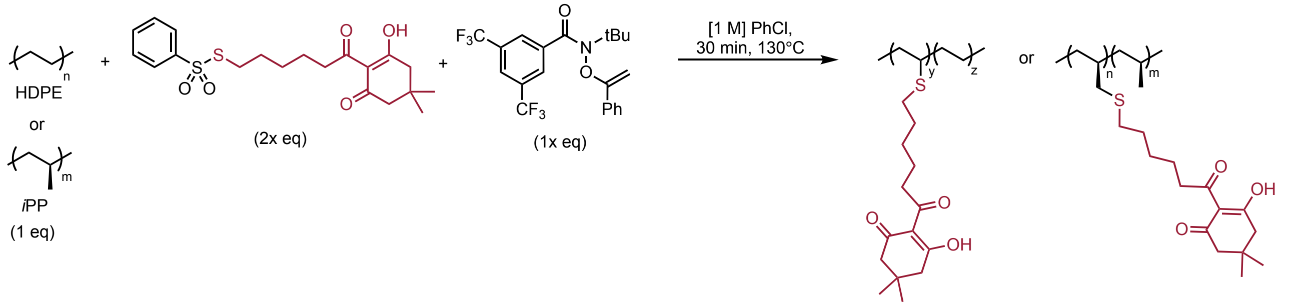

Triketone Functionalization of Polyolefins General Protocol: Polyolefin was placed in a vial with distilled and degassed chlorobenzene under inert atmosphere in a glove box. The mixture was heated at 130 °C for 5 minutes to solubilize the polymer. In a glovebox, amide reagent and triketone trap were dissolved in chlorobenzene and added to the polymer-solvent mixture. The vial was sealed with electrical tape, removed from the glovebox, and placed on a pie block to heat at 130 °C and stir. After the reaction was complete (30 min), the reaction mixture was taken up into a glass pipette and quickly precipitated into a 20 mL scintillation vial ¾ full of stirring acetone. Excellent purity was achieved when acetone was stirring rapidly enough to create a vortex. Functionalized polymer was collected via vacuum filtration and dried overnight under vacuum before characterization. Percent functionalization was calculated by integrating aliphatic polymer peaks to 400 for (HD)PE and 600 for *i*PP, followed by integration of functionalization peaks / #H to determine the percent functionalization.

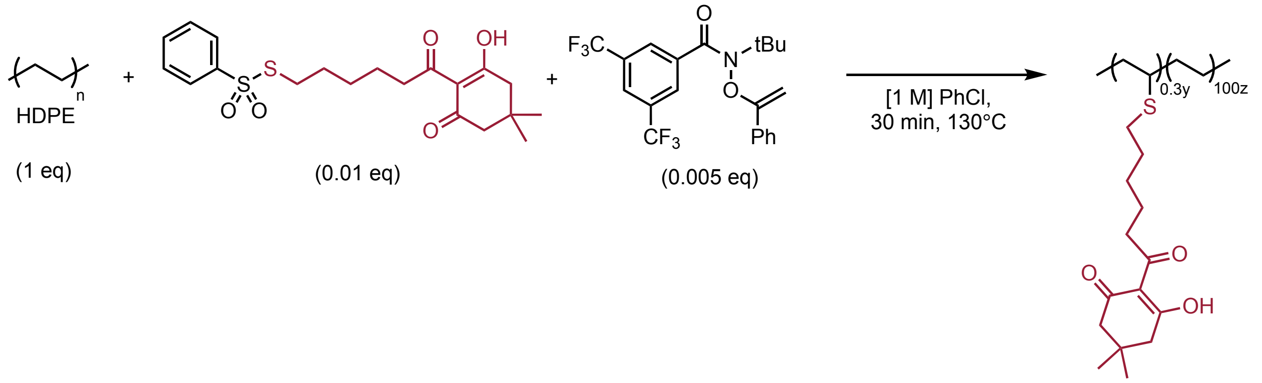

Triketone functionalized HDPE (HDPE-0.3): HDPE (40 mg, 1.4 mmol, 1 eq) was solubilized in PhCl (0.4 mL) at 130 °C before cooling to room temperature. Amide reagent (3.1 mg, 0.005 eq, 7.1 umol) and triketone trap (5.9 mg, 0.01 eq, 14umol) were dissolved in chlorobenzene (1.0 mL PhCl, [1 M] overall) and added to the mixture of polymer-solvent mixture before heating at 130 °C and stirring for 15 minutes. After the reaction was complete, the reaction mixture was

precipitated into a scintillation vial of stirring acetone. Functionalized polymer was collected via vacuum filtration and dried overnight under vacuum before characterization.

^{1}H NMR (500 MHz, $C_2D_2Cl_4$, 110 °C) 17.89 (1H, bs), 3.15 (2H, bm), 2.63 (3H, bm), 2.45 (2H, bm), 1.41-1.02 (1,333H bm) **IR (neat, ATR, cm^{-1})** 2914.22, 28.47.08, 1671.34, 1570.83, 1471.83, 1462.29, 1369.13, 1260.80, 1092.48, 805.27, 718.58, 729.83. **GPC (TCB, 140 °C):** HDPE M_n=30 kg/mol, $Đ$ = 4.3, product M_n = 25 kg/mol, $Đ$ = 3.6. **TGA (°C)** HDPE T_d = 433, product T_d = 445 °C**. DSC (°C):** HDPE T_m = 129 with 62% crystallinity (DH = 183 J/g), product T_m = 126 with 62% crystallinity (DH = 157 J/g).

Percent functionalization was determined through ^{1}H NMR. Peaks corresponding to polyolefin, from 0.8 – 1.8 ppm, were integrated to a total of 400 protons. The *alpha* protons to the thioether that appear between 3.1–3.2 ppm are used to determine mol % functionalization relative to repeat unit. Functionalization was determined to be 0.3 mol%.

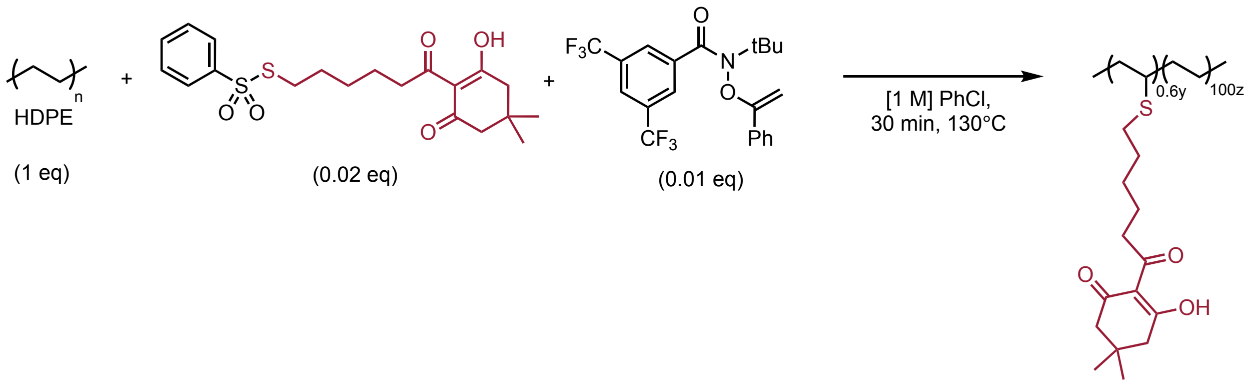

Triketone functionalized HDPE (HDPE-0.6): HDPE (40 mg, 1.4 mmol, 1 eq) was solubilized in PhCl (0.4 mL) at 130 °C before cooling to room temperature. Amide reagent (6.2 mg, 0.01 eq, 14 umol) and triketone trap (12 mg, 0.02 eq, 29 umol) were dissolved in chlorobenzene (1.0 mL PhCl, [1 M] overall) and added to the mixture of polymer-solvent mixture before heating at 130 °C and stirring for 15 minutes. After the reaction was complete, the reaction mixture was precipitated into a scintillation vial of stirring acetone. Functionalized polymer was collected via vacuum filtration and dried overnight under vacuum before characterization.

^{1}H NMR (500 MHz, $C_2D_2Cl_4$, 110 °C) 17.84 (bs, 1H), 3.14 (bs, 2H), 2.60 (bm, 3H), 2.44 (bs, 2H), 1.65-1.01 (bm, 800H). **IR (neat, ATR, cm^{-1})** 2913.97, 2847.25, 1670.42, 1560.44, 1471.45, 1462.74, 1407.04, 1388.14, 1369.44, 1307.99, 1278.15, 1260.29, 1143.91, 1040.95, 729.55, 717.77. **GPC (TCB, 140 °C):** HDPE M_n=30 kg/mol, $Đ$ = 4.4, product M_n = 33 kg/mol, $Đ$ =4.5. **TGA (°C)** HDPE T_d = 433, product T_d = 411 °C. **DSC (°C):** HDPE T_m = 129 with 62% crystallinity (DH = 183 J/g), product T_m = 123 with 42% crystallinity (DH = 124 J/g).

Percent functionalization was determined through ^{1}H NMR. Peaks corresponding to polyolefin, from 0.8 – 1.8 ppm, were integrated to a total of 400 protons. The *alpha* protons to the thioether that appear between 3.1–3.2 ppm are used to determine mol % functionalization relative to repeat unit. Functionalization was determined to be 0.6 mol%.

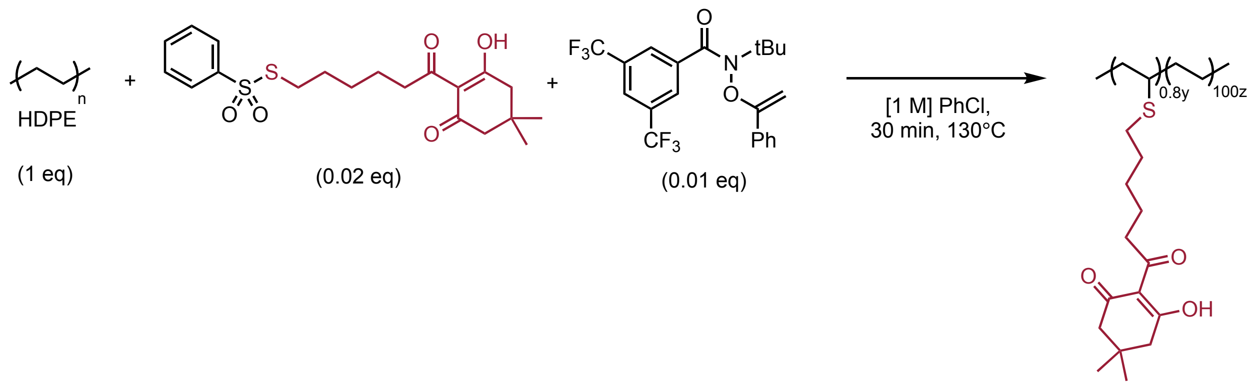

Triketone functionalized HDPE (HDPE-0.8): HDPE (40 mg, 1.4 mmol, 1 eq) was solubilized in PhCl (0.4 mL) at 130 °C before cooling to room temperature. Amide reagent (12mg, 0.02 eq, 29 umol) and triketone trap (23 mg, 0.04 eq, 57 umol) were dissolved in chlorobenzene (1.0 mL PhCl, [1 M] overall) and added to the mixture of polymer-solvent mixture before heating at 130 °C and stirring for 15 minutes. After the reaction was complete, the reaction mixture was precipitated into a scintillation vial of stirring acetone. Functionalized polymer was collected via vacuum filtration and dried overnight under vacuum before characterization.

^{1}H NMR (500 MHz, $C_2D_2Cl_4$, 110 °C) 17.88 (bs, 1H), 3.13 (bm, 2H), 2.59 (bm, 3H), 2.43 (bs, 2H), 1.76-1.01 (bm, 500H). **IR (neat, ATR, cm^{-1})** 2914.32, 2847.19, 1669.79, 1560.47, 1471.60, 1462.34, 1406.91, 1369.38, 1388.06, 1307.93, 1278.17, 1143.39, 1058.79, 729.53, 718.49. **GPC (TCB, 140 °C):** HDPE M_n=30 kg/mol, $Đ$ = 4.4, product M_n = 43 kg/mol, $Đ$ = 3.4. **TGA (°C)** HDPE T_d = 433 °C, product T_d = 353 °C. **DSC (°C):** HDPE T_m = 129 with 62% crystallinity (DH = 183 J/g), product T_m = 121with 37% crystallinity (DH = 110 J/g).

Percent functionalization was determined through ^{1}H NMR. Peaks corresponding to polyolefin, from 0.8 – 1.8 ppm, were integrated to a total of 400 protons. The *alpha* protons to the thioether that appear between 3.1–3.2 ppm are used to determine mol % functionalization relative to repeat unit. Functionalization was determined to be 0.8 mol%.

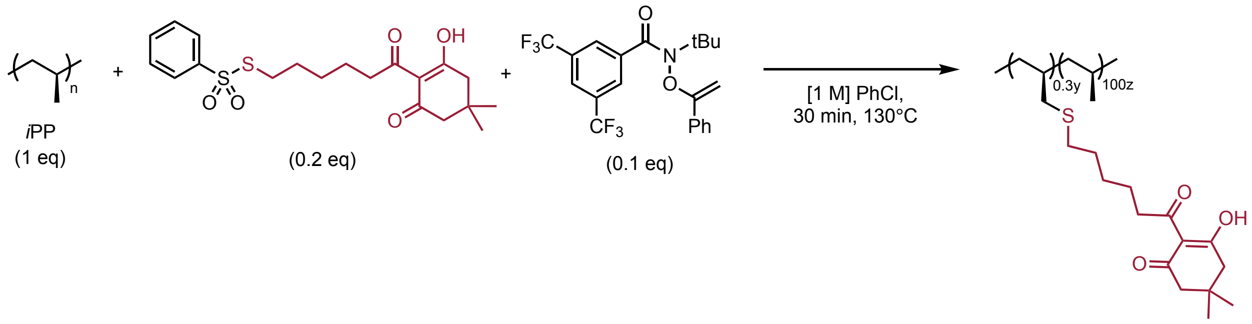

Triketone functionalized *i*PP (*i*PP-0.3): *i*PP (40 mg, 0.95 mmol, 1 eq) was solubilized in PhCl (0.2 mL) at 130 °C before cooling to room temperature. Amide reagent (41 mg, 0.1 eq) and triketone trap (73 mg, 0.2 eq) were dissolved in chlorobenzene (0.5 mL, [1 M] PhCl) and added to the mixture of polymer-solvent mixture before heating at 130 °C and stirring for 30 minutes. After the reaction was complete, the reaction mixture was precipitated into a scintillation vial of stirring

acetone. Functionalized polymer was collected via vacuum filtration and dried overnight under vacuum before characterization.

^{1}H NMR (500 MHz, $C_2D_2Cl_4$, 110 °C) δ 3.15 (bs, 2H), δ 2.65 (bs, 3H), δ 2.45 (bs, 2H), (bs) 1.93 – 0.73 (m) ppm. **IR (neat, ATR, cm^{-1})** 2949, 2917, 2867, 2838, 1672, 1454, 1376, 1359, 1168, 998, 973, 899, 841, 808. **GPC (TCB, 140 °C):** *i*PP M_n=62 kg/mol, $Đ$ = 5.1, product M_n = 61 kg/mol, $Đ$ = 6.5. **TGA (°C)** *i*PP T_d = 241, product T_d = 338. **DSC (°C)** parent T_m = 143 (DH = 77 J/g), product T_m = 153 (DH = 124 J/g).

Percent functionalization was determined through ^{1}H NMR. Peaks corresponding to polyolefin, from 0.8 – 1.8 ppm, were integrated to a total of 600 protons. The *alpha* protons to the thioether that appear between 3.1–3.2 ppm are used to determine mol % functionalization relative to repeat unit. Functionalization was determined to be 0.3 mol%.

Triketone functionalized LLDPE (LLDPE-0.1): LLDPE (40 mg, 1.4 mmol, 1 eq) was solubilized in PhCl (0.2 mL) at 130 °C before cooling to room temperature. Amide reagent (0.0025 eq) and triketone trap (0.005 eq) were dissolved in chlorobenzene (0.5 mL, [2 M] PhCl) and added to the mixture of polymer-solvent mixture before heating at 130 °C and stirring for 30 min. After the reaction was complete, the reaction mixture was precipitated into a scintillation vial of stirring acetone. Functionalized polymer was collected via vacuum filtration and dried overnight under vacuum before characterization.

^{1}H NMR (500 MHz, EtD2Cl4) δ 3.15 (t, 2H), 2.62 (m, 3H), 2.44 (s, 2H), 1.79 (m), 1.76 (m), 1.61 (m), 1.51(bs), 1.39 (bs), 1.18 (s, 6H), 1.01 (s) ppm. **IR (neat, ATR, cm^{-1})** 2915, 2847, 1670, 1560, 1472, 1462, 730, 719. **GPC (TCB, 140 °C)**: LLDPE M_n=21 kg/mol, $Đ$ = 3.6, product M_n 22= kg/mol, $Đ$ =3.4. **TGA (°C)**: LLDPE T_d =428, product T_d =282. **DSC (°C)**: 12 parent T_m = 125 with 41% crystallinity (DH = 122 J/g), product T_m = 123 with 37% crystallinity (DH = 110 J/g).

Percent functionalization was determined through ^{1}H NMR. Peaks corresponding to polyolefin, from 0.8–1.8 ppm, were integrated to a total of 400 protons. The alpha protons to the thioether that appear between 3.1–3.2 ppm are used to determine mol % functionalization relative to repeat unit. As an average of 3 trials, functionalization was determined to be 0.1 mol%.

b. Unmodified blend preparation

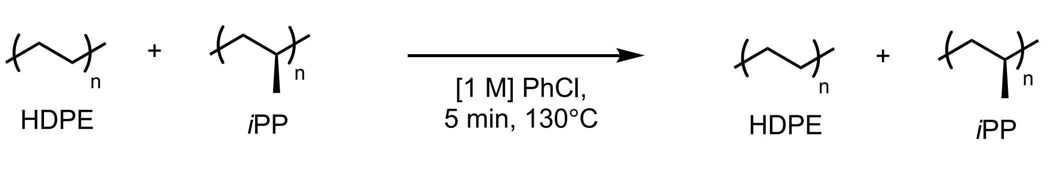

Unmodified HDPE-*i*PP blend (UB): HDPE and *i*PP were combined in a 70:30 wt% ratio in a vial with distilled and degassed chlorobenzene [2 M] under inert atmosphere in a glove box. The mixture was heated at 130 °C for 5 minutes to solubilize and mix the polymers before precipitation in stirring acetone and collection via vacuum filtration.

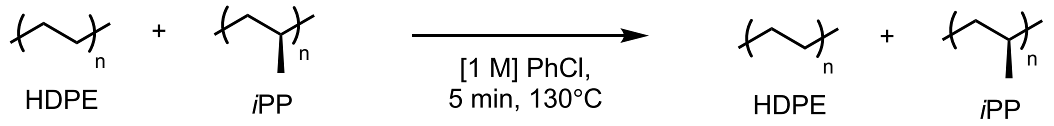

Unmodified 50–50 HDPE-*i*PP blend (UB2): HDPE and *i*PP were combined in a 50:50 wt% ratio in a vial with distilled and degassed chlorobenzene [2 M] under inert atmosphere in a glove box. The mixture was heated at 130 °C for 5 minutes to solubilize and mix the polymers before precipitation in stirring acetone and collection via vacuum filtration.

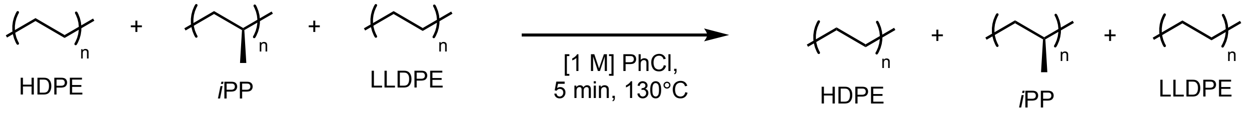

Unmodified HDPE-*i*PP-LLDPE blend (UB3): HDPE and *i*PP and LLDPE were combined in a 28:36:36 wt% ratio in a vial with distilled and degassed chlorobenzene [2 M] under inert atmosphere in a glove box. The mixture was heated at 130 °C for 5 minutes to solubilize and mix the polymers before precipitation in stirring acetone and collection via vacuum filtration.

c. Diketoenamine crosslinked blend synthesis

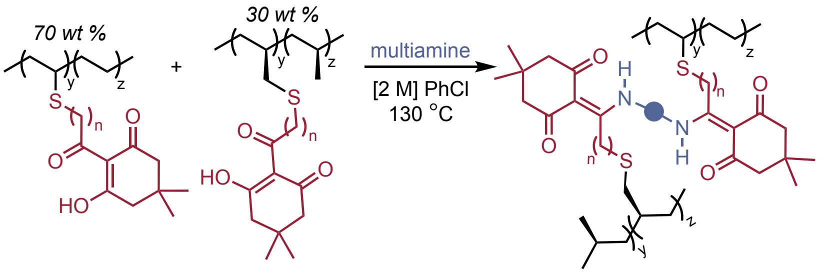

MultiAmine Post-Functionalization Crosslinking of Polyolefins General Protocol: A 70:30 ratio of triketone functionalized HDPE and triketone functionalized *i*PP was placed in a vial with distilled and degassed chlorobenzene under inert atmosphere in a glove box. The mixture was heated at 130 °C for 5 minutes to solubilize the polymer. In a glovebox, multiamine and additional chlorobenzene were added to the polymer-solvent mixture. The vial was sealed with electrical tape, removed from the glovebox, and stirred at room temperature for 2 minutes to achieve good mixing of amine and functionalized polyolefin. Next, the reaction was stirred and heated on a pie block at 130 °C to react. The stir bar stopped stirring after 5–20 minutes as a result of gel formation and was dependent upon amine identity. After the reaction was complete, the gel was removed from the vial and placed into a scintillation vial ¾ full of stirring acetone. The remaining chlorobenzene solution was added to the vial of stirring acetone. Crosslinked polymer was collected via vacuum filtration and was dried overnight under vacuum before characterization.

Non-crosslinked Blend (NB): A 70:30 wt% ratio of 0.3 mol% triketone functionalized HDPE and 0.3 mol% triketone functionalized *i*PP (350 mg triketone HDPE) (150 mg triketone *i*PP) was solubilized in PhCl (6 mL) at 130 °C before cooling to room temperature. *N*,*N*-diethyl ethylene

diamine was added (100 eq relative to triketone, 149 uL) followed by the addition of PhCl (2.8 mL for a total of 6.8 mL [2 M]). The reaction was stirred for 2 minutes at room temperature before stirring and heating at 130 °C for 12 minutes under inert atmosphere. After the reaction was complete, the gel was removed from the vial and placed in a vial of stirring acetone before collection via vacuum filtration and was dried overnight under vacuum before characterization.

The product could not be characterized by ^{1}H NMR as crosslinks result in an insoluble material. **IR (neat, ATR, cm^{-1})** 2959, 2949, 2915, 2848, 1577, 1560, 1473, 1462, 1375, 1332, 1358, 1303, 1262, 1168, 1102, 998, 972, 841, 809, 730, 719. **TGA (°C)** parent T_d =425 °C, product T_d = 399 °C. **DSC (°C):** T_m HDPE=127 °C, T_m *i*PP= 152 °C , DH HDPE = 96 J/g, DH *i*PP = 17 J/g.

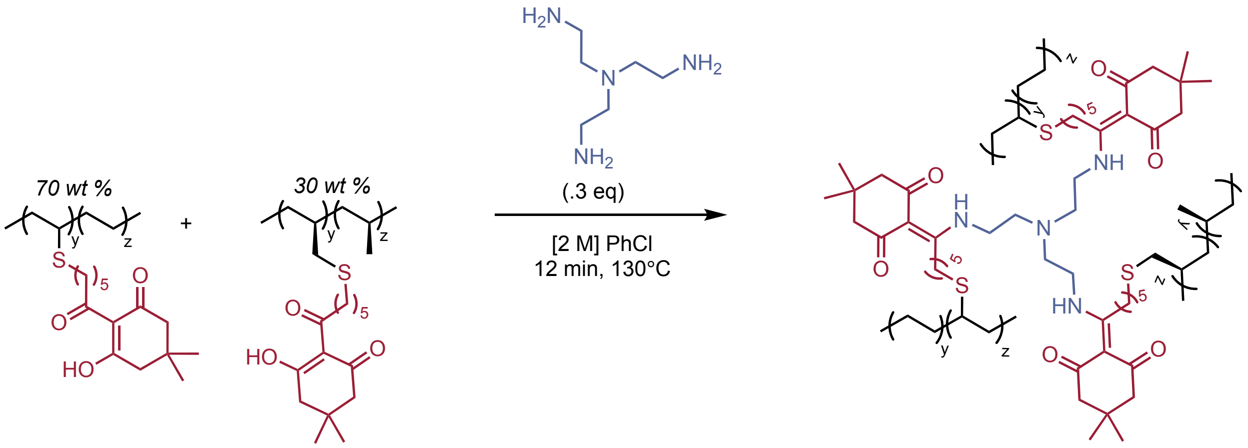

Diketoenamine Crosslinked Blend (B1): A 70:30 wt% ratio of 0.6 mol% triketone functionalized HDPE and 0.3 mol% triketone functionalized *i*PP (350 mg triketone HDPE) (150 mg triketone *i*PP) was solubilized in PhCl (6 mL) at 130 °C before cooling to room temperature. TREN was added from a stock solution (1 eq relative to triketone, 6.7 uL) followed by the addition of PhCl (2.8 mL for a total of 6.8 mL [2 M]). The reaction was stirred for 2 minutes at room temperature before stirring and heating at 130 °C for 12 minutes under inert atmosphere. After the reaction was complete, the gel was removed from the vial and placed in a vial of stirring acetone before collection via vacuum filtration and drying overnight under vacuum before characterization.

The product could not be characterized by ^{1}H NMR as crosslinks result in an insoluble material. **IR (neat, ATR, cm^{-1})** 2949, 2914, 2848, 1647, 1576, 1471, 1463, 1376, 1261, 1166, 1100, 998, 973, 841, 807, 730, 717. **TGA (°C)** parent T_d = 425 °C, product T_d = 404 °C. **DSC (°C):** T_m HDPE= 120 °C, T_m *i*PP= 152 °C , DH HDPE = 64 J/g, DH *i*PP = 16 J/g.

Diketoenamine Crosslinked Blend (B2): A 70:30 wt% ratio of 0.3 mol% triketone functionalized HDPE and 0.3 mol% triketone functionalized *i*PP (350 mg triketone HDPE) (150 mg triketone *i*PP) was solubilized in PhCl (6 mL) at 130 °C before cooling to room temperature. TREN was added from a stock solution (0.33 eq TREN relative to triketone, 2.2 uL TREN) followed by the addition of PhCl (2.8 mL for a total of 6.8 mL [2 M]). The reaction was stirred for 2 minutes at room temperature before stirring and heating at 130 °C for 12 minutes under inert atmosphere. After the reaction was complete, the gel was removed from the vial and placed in a vial of stirring

acetone before collection via vacuum filtration and drying overnight under vacuum before characterization.

The product could not be characterized by ^{1}H NMR as crosslinks result in an insoluble material. **IR (neat, ATR, cm^{-1})** 2915, 2847, 1670, 1577, 1560, 1555, 1534, 1473, 1462, 1376, 1304, 1262, 1097, 1023, 841, 840, 730, 719. **TGA (°C)** parent T_d = 425 °C, product T_d = 281 °C. **DSC (°C):** T_m HDPE= 123 °C, T_m *i*PP= 154 °C, DH HDPE = 78 J/g, DH *i*PP = 18 J/g.

Diketoenamine Crosslinked Blend (B3): A 70:30 wt% ratio of 0.8 mol% triketone functionalized HDPE and 0.3 mol% triketone functionalized iPP (350 mg triketone HDPE) (150 mg triketone iPP) was solubilized in PhCl (6 mL) at 130 °C before cooling to room temperature. TREN was added from a stock solution (0.3 eq relative to triketone, 5.6 uL) followed by the addition of PhCl (2.8 mL for a total of 6.8 mL [2 M]). The reaction was stirred for 2 minutes at room temperature before stirring and heating at 130 °C for 12 minutes under inert atmosphere. After the reaction was complete, the gel was removed from the vial and placed in a vial of stirring acetone before collection via vacuum filtration and drying overnight under vacuum before characterization.

The product could not be characterized by ^{1}H NMR as crosslinks result in an insoluble material. **IR (neat, ATR, cm^{-1})** 2915, 2848, 1670, 1577, 1560, 1555, 1534, 1473, 1462, 1376, 1304, 1262, 1097, 1023, 841, 804, 730, 719, 477, 436. **TGA (°C)** parent T_d = 425 °C, product T_d = 290 °C. **DSC (°C):** T_m HDPE=114 °C, T_m *i*PP= 154 °C , DH HDPE = 63 J/g, DH *i*PP = 23J/g.

Diketoenamine Crosslinked Blend (B4): A 70:30 wt% ratio of 0.3 mol% triketone functionalized HDPE and 0.3 mol% triketone functionalized *i*PP (350 mg triketone HDPE) (150 mg triketone *i*PP) was solubilized in PhCl (2 mL) at 130 °C before cooling to room temperature. **Dodecyldiamine** was added from a stock solution (1 eq relative to triketone, 6.7 uL) followed by the addition of PhCl (2.8 mL for a total of 6.8 mL [2 M]). The reaction was stirred for 2 minutes at room temperature before stirring and heating at 130 °C for 20 minutes under inert atmosphere (required longer reaction times compared to reactions with TREN). After the reaction was complete, the gel was removed from the vial and placed in a vial of stirring acetone before collection via vacuum filtration and drying overnight under vacuum before characterization.

The product could not be characterized by ^{1}H NMR as crosslinks result in an insoluble material. **IR (neat, ATR, cm^{-1})** 2950, 2915, 2848, 1670, 1643, 1574, 1471, 1462, 1376, 1304, 1261, 1167, 1143, 1101, 1037, 998, 973, 841, 807, 730, 719. **TGA (°C)** parent T_d = 425 °C, product T_d = 310 °C. **DSC (°C):** T_m HDPE=116 °C, T_m *i*PP= 152 °C , DH HDPE = 69 J/g, DH *i*PP = 13 J/g.

Diketoenamine Crosslinked 50–50 Blend (B5): HDPE that was 0.3 mol% triketone functionalized with triketone (500 mg triketone HDPE) was solubilized in PhCl (2 mL) at 130 °C before cooling to room temperature. TREN was added from a stock solution (1 eq relative to triketone, 6.7 uL) followed by the addition of PhCl (2.8 mL for a total of 6.8 mL [2 M]). The reaction was stirred for 2 minutes at room temperature before stirring and heating at 130 °C for 12 min under inert atmosphere. After the reaction was complete, the gel was removed from the vial and placed in a vial of stirring acetone before collection via vacuum filtration and drying overnight under vacuum before characterization.

Diketoenamine Crosslinked Ternary Blend (B6): A 70:30 wt% ratio of 0.3 mol% triketone functionalized HDPE and 0.3 mol% triketone functionalized *i*PP (350 mg triketone HDPE) (150 mg triketone *i*PP) was solubilized in PhCl (2 mL) at 130 °C before cooling to room temperature. TREN was added from a stock solution (1 eq relative to triketone, 6.7 uL) followed by the addition of PhCl (2.8 mL for a total of 6.8 mL [2 M]). The reaction was stirred for 2 minutes at room temperature before stirring and heating at 130 °C for 12 minutes under inert atmosphere. After the reaction was complete, the gel was removed from the vial and placed in a vial of stirring acetone before collection via vacuum filtration and drying overnight under vacuum before characterization.

Diketoenamine Crosslinked HDPE: 0.3 mol% triketone functionalized HDPE, 0.3 mol% (500 mg triketone HDPE) was solubilized in PhCl (2 mL) at 130 °C before cooling to room temperature. TREN was added from a stock solution (1 eq relative to triketone, 6.7 uL) followed by the addition of PhCl (2.8 mL for a total of 6.8 mL [2 M]). The reaction was stirred for 2 minutes at room temperature before stirring and heating at 130 °C for 12 min under inert atmosphere. After the reaction was complete, the gel was removed from the vial and placed in a vial of stirring acetone before collection via vacuum filtration and drying overnight under vacuum before characterization.

IV. Additional Blend Characterization

a. Differential Scanning Calorimetry Thermograms

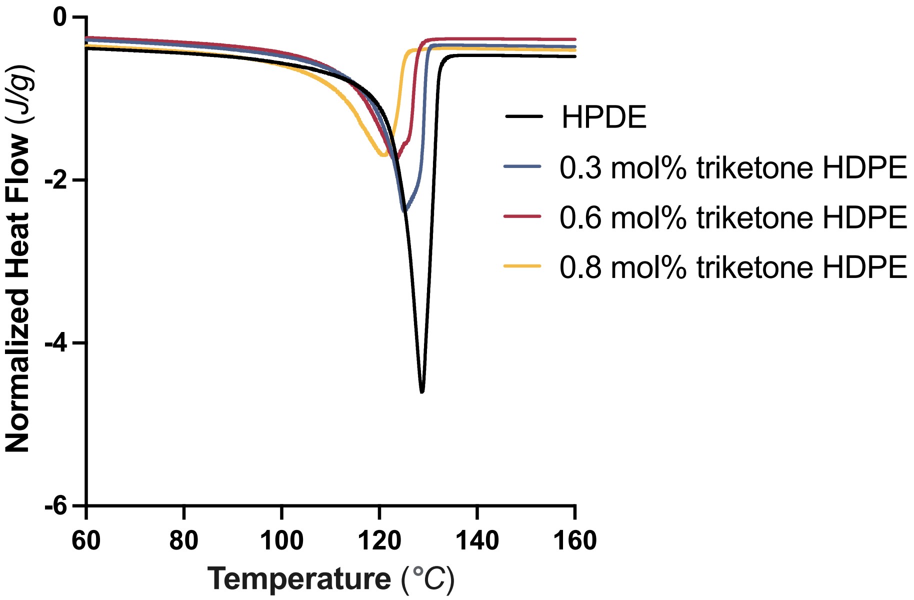

Figure S24: DSC of triketone functionalized HDPEs in comparison to HDPE reveals melting temperatures and enthalpies of melting of HDPE crystallites. Functionalization results in a

decrease in the melt temperature and enthalpy of melting. Data obtained from the 2nd heating cycle at a heating rate of 10 °C/min.

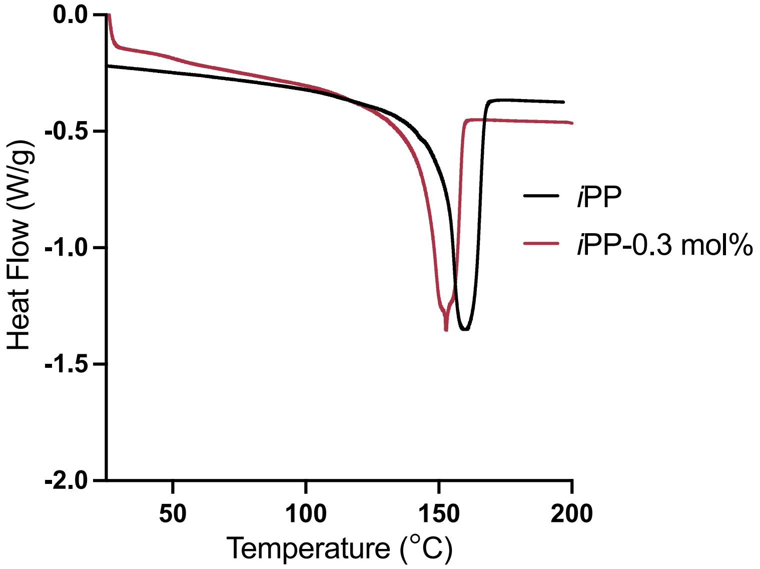

Figure S25: DSC of triketone functionalized *i*PPs in comparison *i*PP to reveals melting temperatures and enthalpies of melting of *i*PP crystallites. Functionalization results in a small decrease in the melt temperature and enthalpy of melting. Data obtained from the 2nd heating cycle at a heating rate of 10 °C/min.

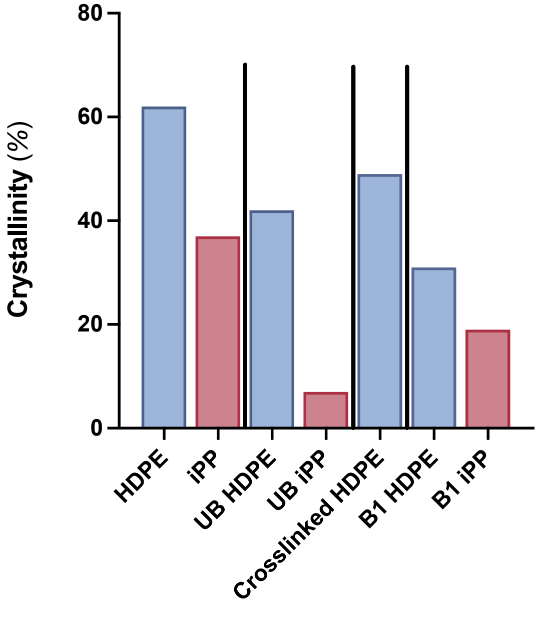

Figure S26: Comparison of the percent crystallinity of the HDPE and *i*PP phases in the homopolymers, unmodified blend (**UB**), and the dynamically crosslinked blend (**B1**) determined by Data obtained from the 2nd heating cycle at a heating rate of 10 °C/min.

b. Cryo-ultramicrotome

To prepare thin samples for TEM, AFM-IR and 4D-STEM, melt pressed films were cryo-sectioned using a Leica Em FC7 cryo-ultramicrotome at -60 ºC to limit structural deformation of low T_g polyolefins. A small piece (ca. 1 mm x 1 mm x 0.2 mm) of the film was affixed to a sample holder with super glue. Samples were cut perpendicular to the film surface unless otherwise noted. Samples were prepared at 100, 200, and 330 nm thickness by cutting at 20 mm/s. 200 nm thickness proved to be a desirable middle ground with limited film fracture and adequate transmission. A diamond blade was used with an attached pool for sample collection. Samples were sliced onto a pool of 60:40 wt% DMSO:H_2O, a eutectic solution to facilitate efficient sample collection. Samples floated on the pool surface and were collected using a hoop and were placed onto a copper TEM grids with carbon mesh backing.

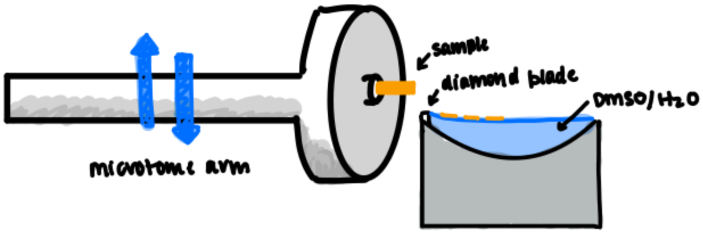

Figure S27: Diagram of the preparation of polymer blend cryosections.

Samples were stained for TEM imaging with a RuO_4 stain (and were not stained for AFM-IR nor 4D-STEM). Caution: RuO_4 is highly toxic and should only be handled in a fume hood with adequate PPE. The contrast stain was prepared immediately before use by mixing 15 mg of $RuCl_3$ with 1 mL of NaOCl solution (10–15% available chlorine). The solution was placed in a glass Petrie dish the lid was placed on. Then samples on the TEM grids and dish containing the RuO_4 solution were placed into a larger Petrie dish (Petrie dish 1). The large lid was placed on Petrie dish 1 and the samples were exposed to the stain for 2 h. Samples were left in the fume hood for 2 h before removing.

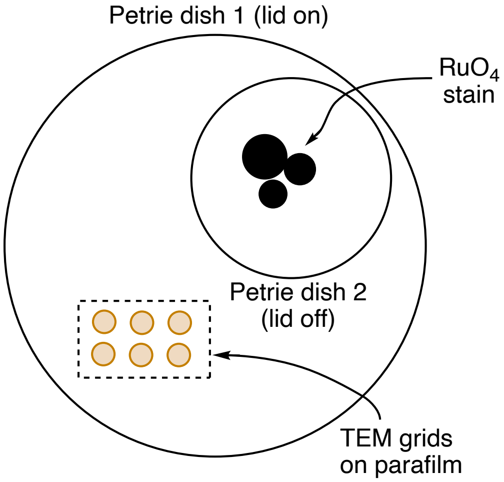

Figure S28: Diagram of the staining of samples for TEM.

c. Transmission electron microscopy (TEM)

Stained samples on copper grids were loaded into the TEM and were studied at room temperature under high vacuum at an accelerating voltage of 200 kV. Images were analyzed using image J software.

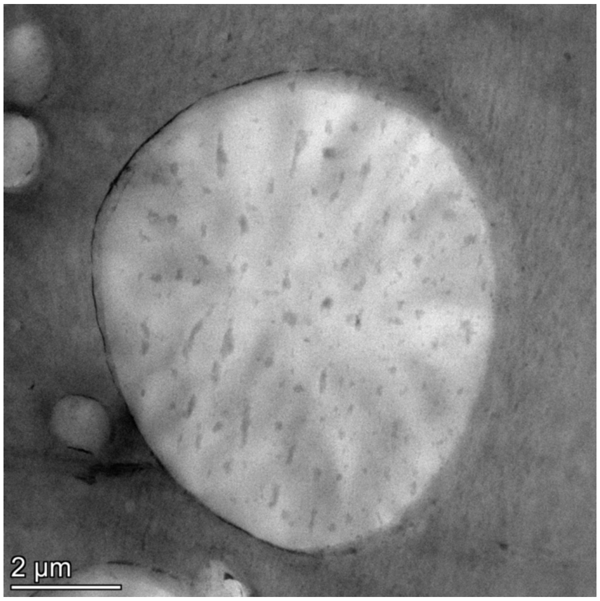

Figure S29: TEM of **UB** shows an *i*PP droplet morphology, RuO_4 stain for enhanced contrast.

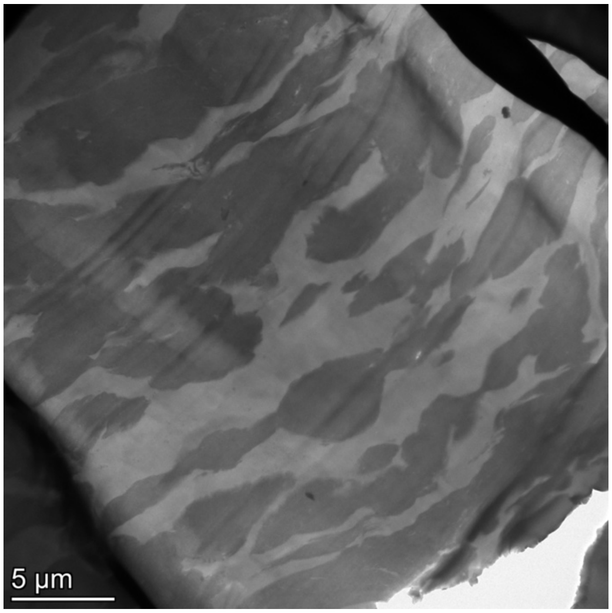

Figure S30: TEM of **B1** in the direction perpendicular to the film surface, RuO_4 stain for enhanced contrast.

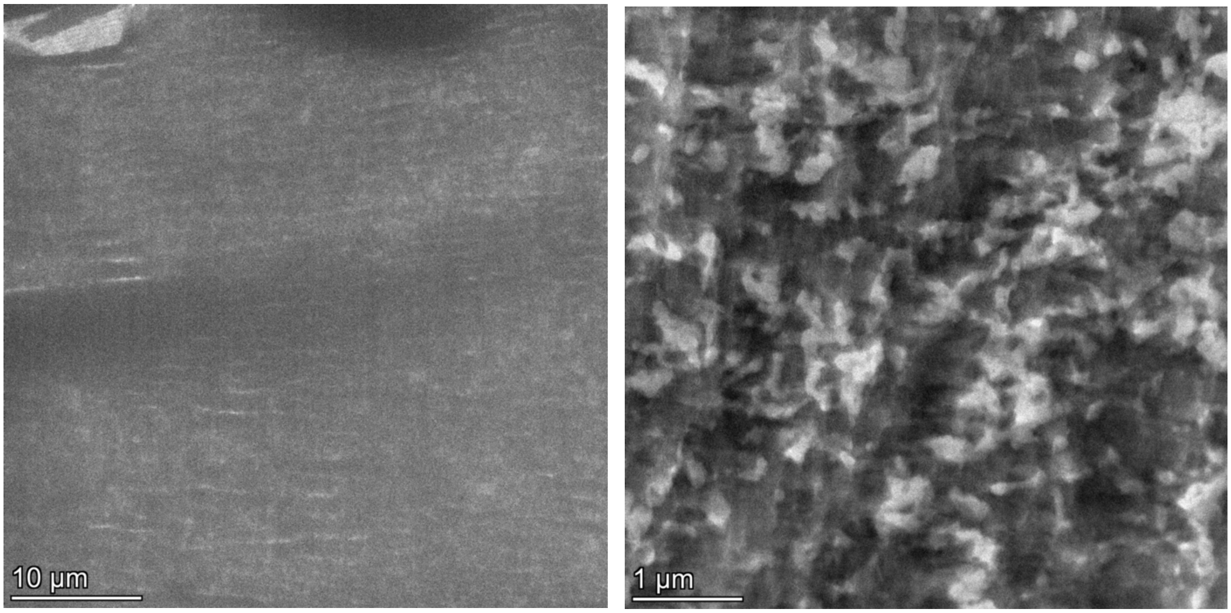

Figure S31: TEM of **NB**, RuO_4 stain for enhanced contrast.

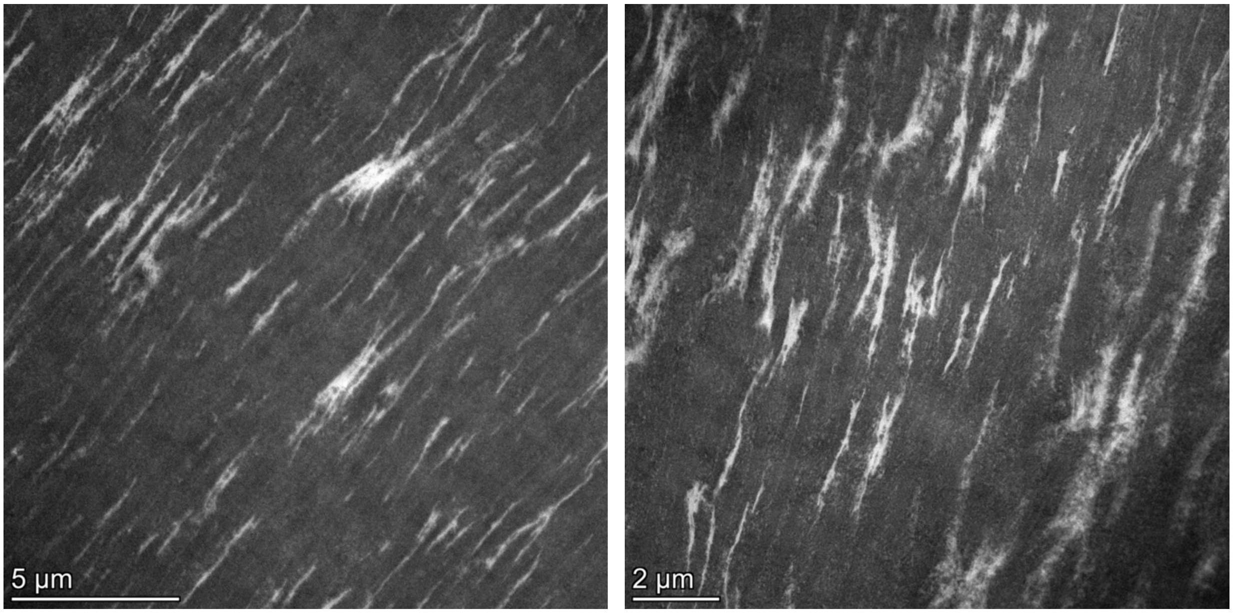

Figure S32: TEM of **B4**, RuO_4 stain for enhanced contrast.

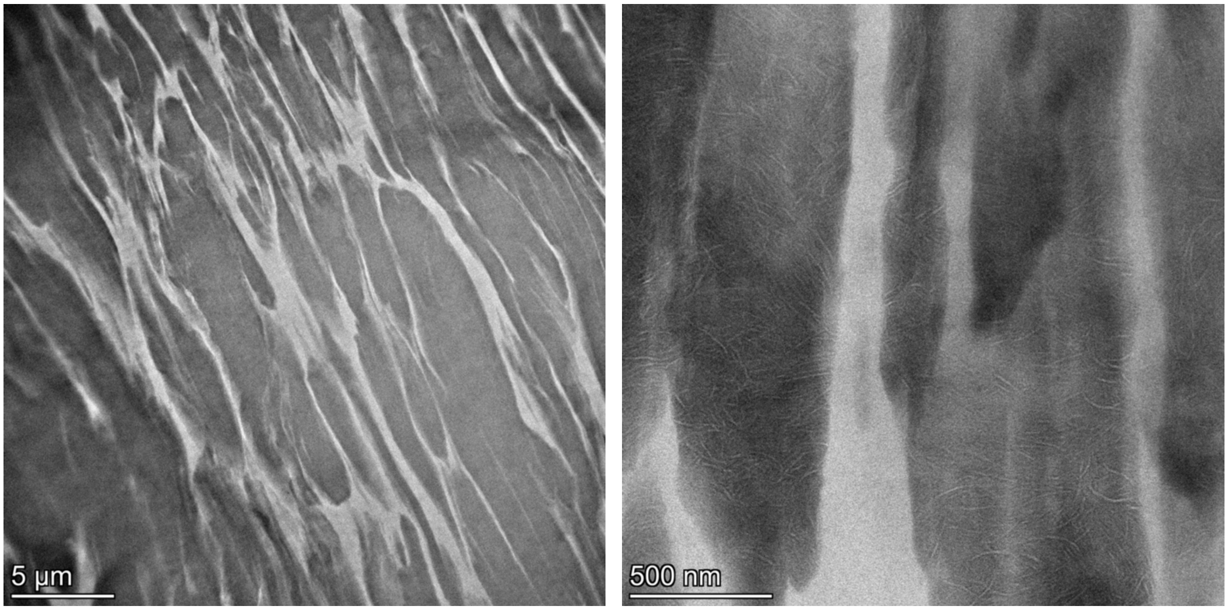

Figure S33: TEM of **B2**, RuO_4 stain for enhanced contrast. (left) striated morphology (right) lamellae are visible in the TEM image as thin lines of lighter stain.

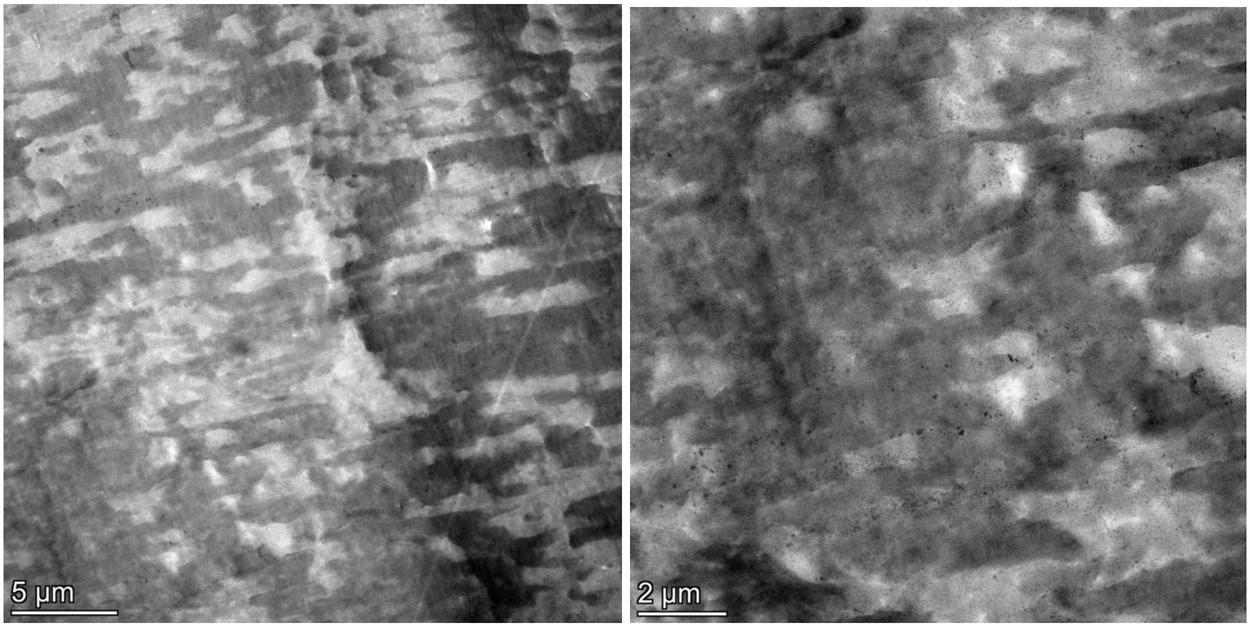

Figure S34: TEM of the unmodified 50–50 *i*PP–HDPE polymer blend, RuO_4 stain for enhanced contrast.

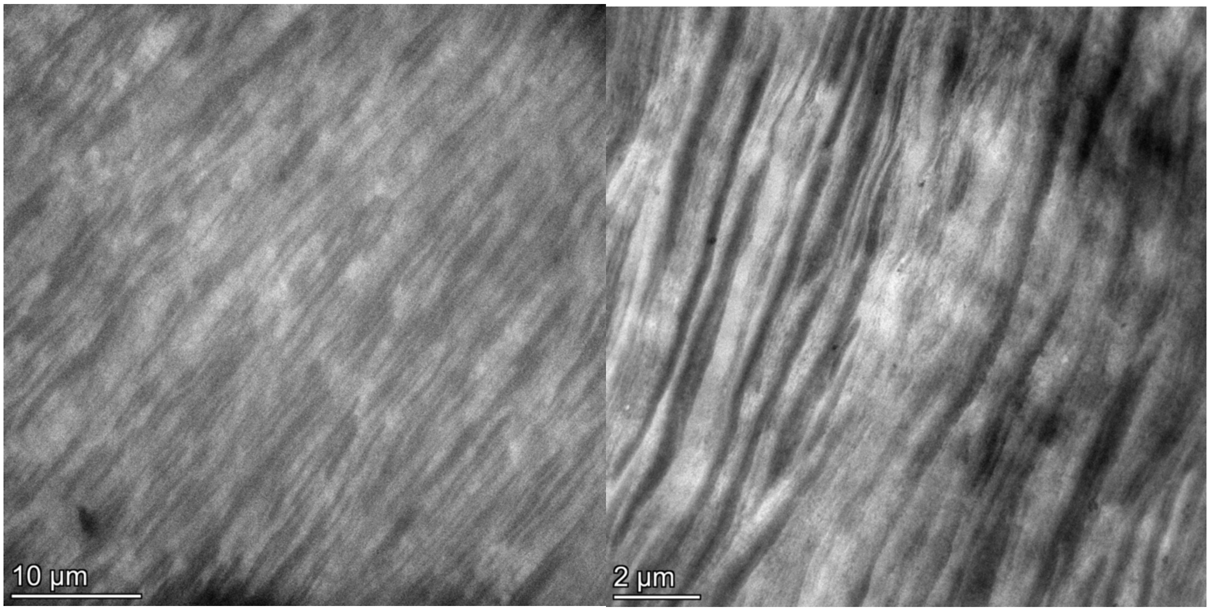

Figure S35: TEM of dynamically crosslinked 50–50 *i*PP–HDPE polymer blend, RuO_4 stain for enhanced contrast.

d. AFM-IR

Samples were prepared by the cryosectioning of melt pressed films using a cryomicrotome. Samples were sliced into a 200 nm thickness by cutting at 20 mm/s. A diamond blade was used. Samples were sliced onto a pool of 60:40 wt% DMSO:H_2O, a eutectic solution. Samples floated on the pool and were collected using a hoop and were placed onto a silicon chip.

AFM-IR measurements were performed using a Bruker Anasys nanoIR3 and measured in tapping mode. The system uses a pulsed, tunable IR laser provided from a quantum cascade laser (QCL) and focuses it onto the AFM tip. Each AFM scan provides a topography and phase map of the specified area, and by varying the wavenumber of the incident IR laser, an IR intensity map will also be collected concurrently. By repeating scans at different wavenumbers, a set of IR maps was obtained of the same area. Alternatively, by keeping the tip stationary and sweeping through the available spectral range of the QCL, individual IR spectra are obtained at different locations.

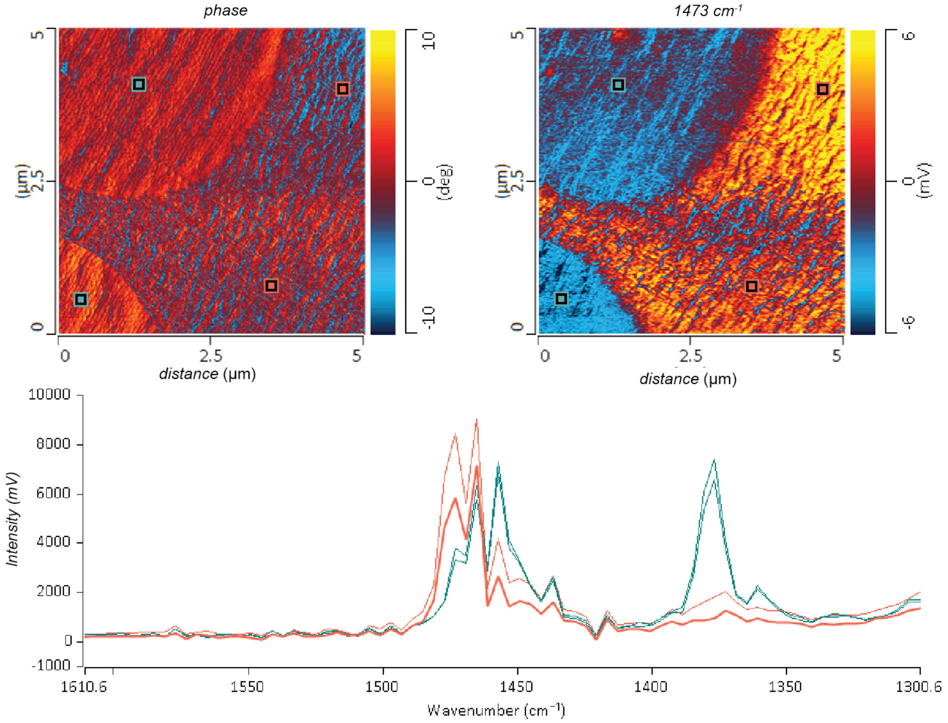

Figure S36: AFM-IR point spectra taken in both the HDPE and *i*PP phases for **UB**. Spectra are cropped to measure from 1300–1610 cm^{-1}. The green markers and spectra depict the spectra for the *i*PP and the orange depicts the HDPE. The peak at 1473 cm^{-1} originates from the methylene vibrational mode while the peak at 1377 cm^{-1} and 1457 cm^{-1} originates form the methyl group.

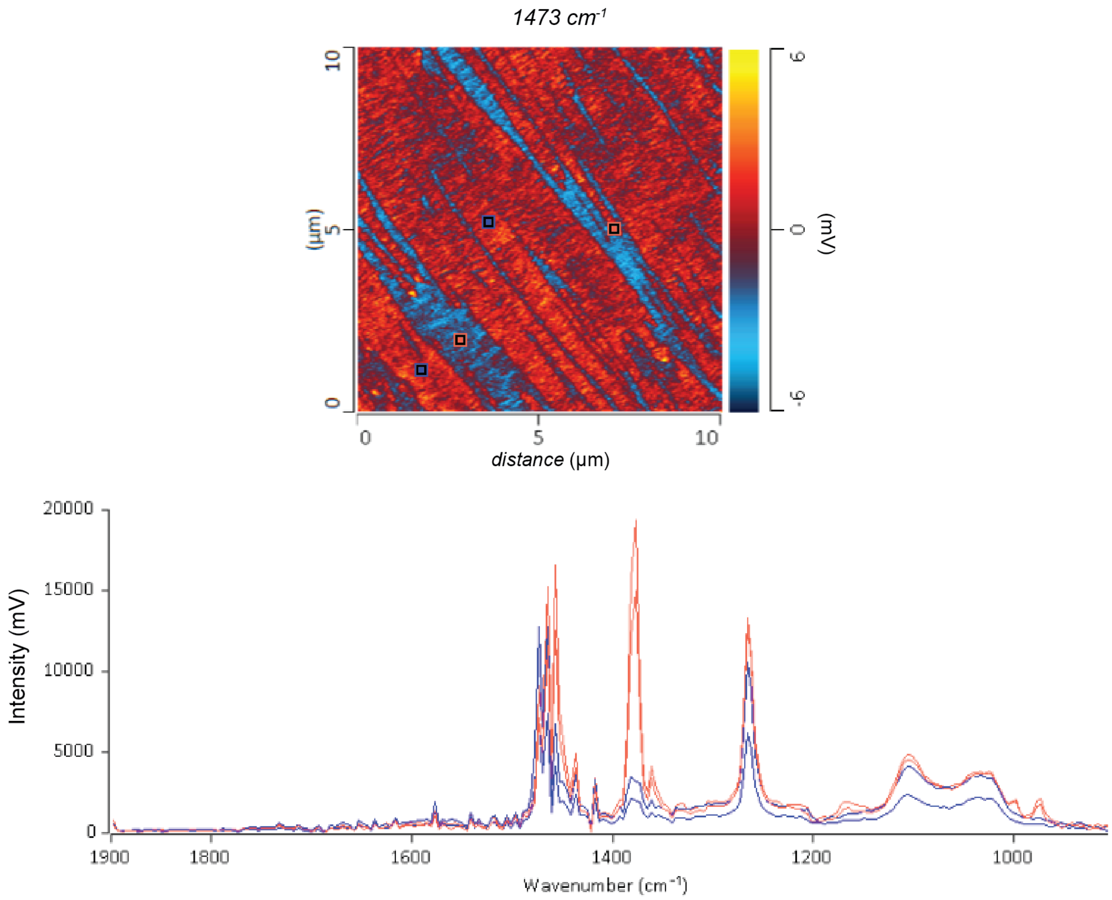

Figure S37: AFM-IR point spectra taken in both phases for **B1**. Spectra were taken of the full available range from 900–1900 cm^{-1}.

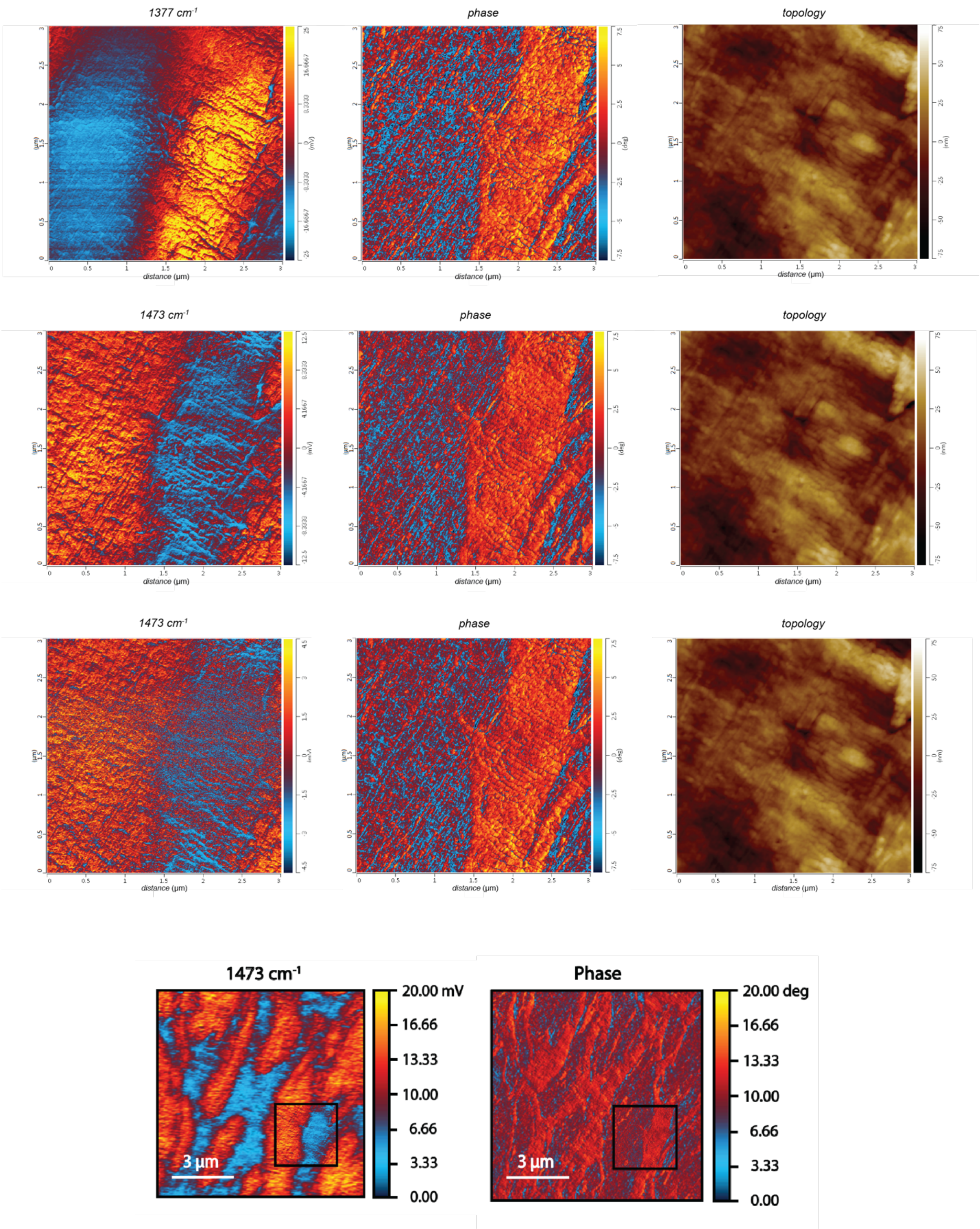

Figure S38: The topography, phase, and IR intensity maps of **B1** at wavenumbers corresponding to the vibrational modes. AFM-IR scans were taken at a 3 x 3 µm scan size and a 1024 x 1024 resolution. A larger scan of the surrounding area was also taken to better depict the location, at a scan size of 10 x 10 µm scan size at a 256 x 256 resolution.

e. **X-ray scattering**

WAXS:

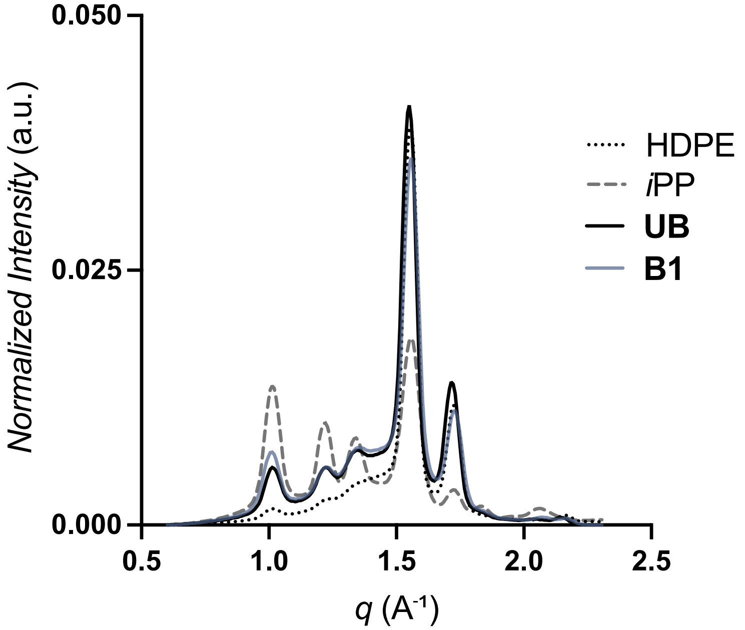

Figure S39: Overlay of wide angle X-ray scattering data for *i*PP, HDPE, the unmodified blend (**UB**) and the dynamically crosslinked blends (**B1**).

SAXS:

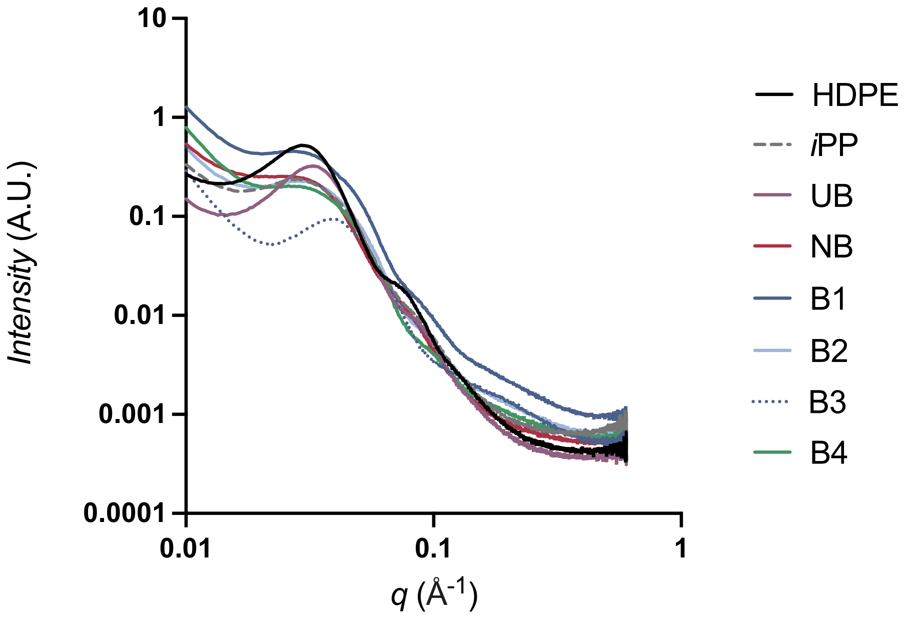

Figure S40: Small-angle X-ray scattering (SAXS) data for HDPE, *i*PP, the unmodified blend (**UB)**, the non-crosslinked blend (**NB**), and dynamically crosslinked blends **B1–B4**. Dynamic crosslinking shifts the Guineir regime to higher q, consistent with a reduction in crystallite size. Analysis of the primary SAXS peak indicates that with crosslinking the inter-crystalline domain spacing becomes more disperse upon dynamic crosslinking. The second-order reflection at ca. 0.1 becomes less pronounced in the dynamically crosslinked materials, reflecting less order in comparison to the homopolymers.

**Polymer**	**Lc HDPE (nm)**	**Lc *i*PP (nm)**
HDPE	14.9	
*i*PP		13.2
Unmodified blend (**UB**)	14.3	11.9
Noncrosslinked blend (**NB**)	12.8	12.1
**B1**	12.4	10.7
**B2**	11.8	10.1
**B3**	11.4	10.6
**B4**	12.4	10.6

Table S2: Wide-angle X-ray scattering (WAXS) derived crystallite thickness (Lc) for HDPE, *i*PP, the unmodified blend (**UB)**, the non-crosslinked blend (**NB**), and dynamically crosslinked blends **B1–B4.** Overall, both blending and dynamic crosslinking result in a decrease in crystallite thickness. Lc was determined using Scherrer equation.

f. Crystallization kinetics

The domains for crystallization nucleation were determined by isothermal steps at a T_c (seeding or self-nucleation temperature) where

Domain 1= complete melting domain (T_c remains low)
Domain 2=nucleation domain (T_c increases)
Domain 3=nucleation and annealing domain (T_c has increased and T_m increases)

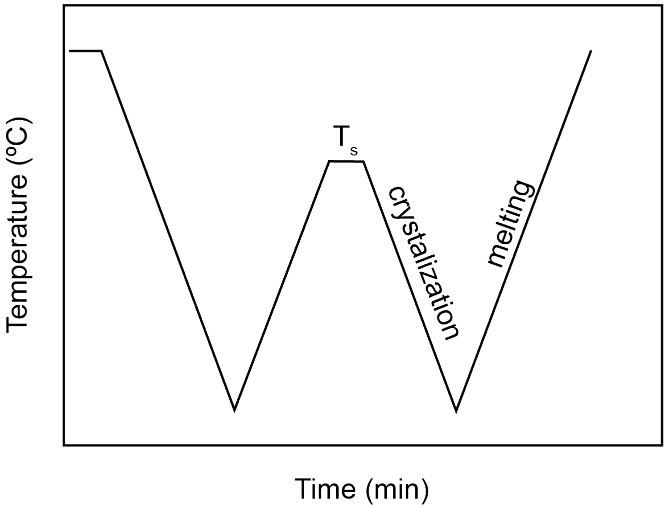

Figure S41: Temperature profile for self-nucleation experiments by annealing at self-nucleation temperatures (T_s) to determine the domains of crystallite nucleation and growth.

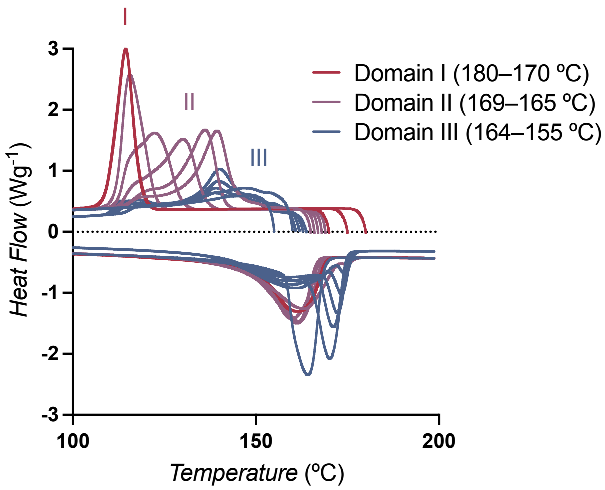

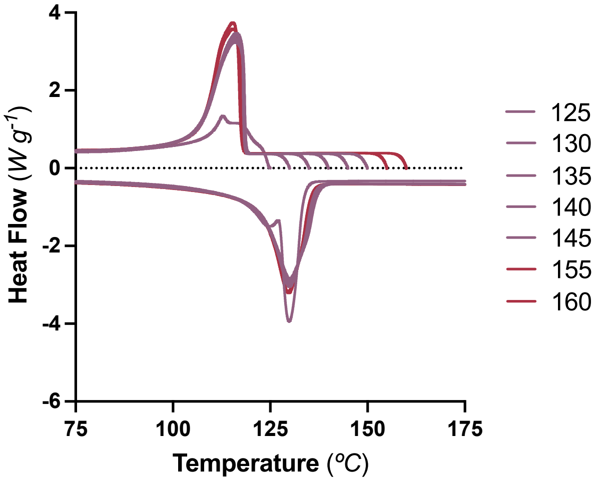

Figure S42: Crystallization kinetics experiments for the homopolymers. (left) *i*PP reaches domain II at **169 ºC**; (right) HDPE (potentially) hits domain 2 at **150 ºC**.

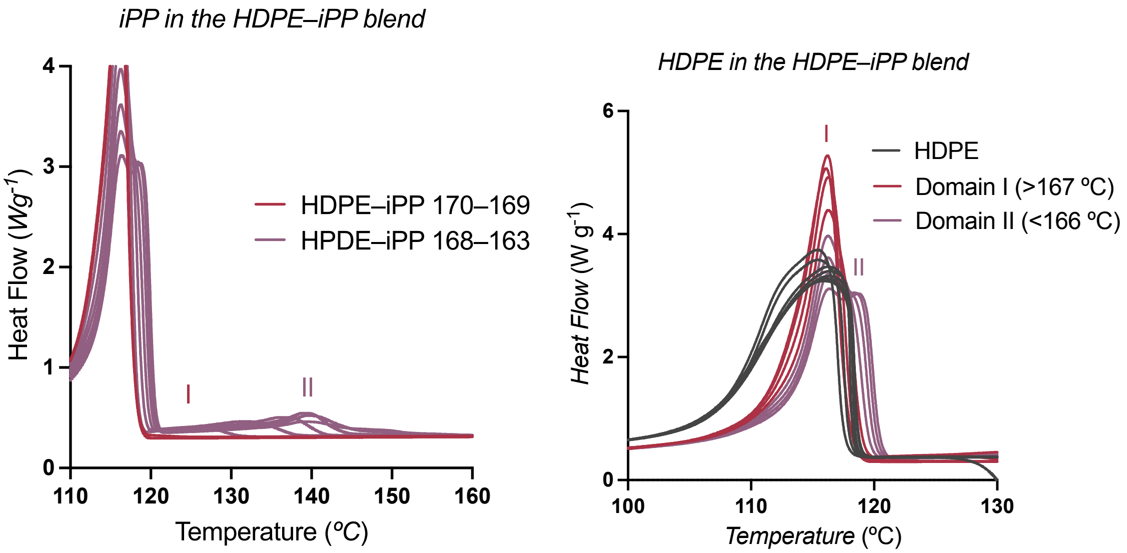

Figure S43: Crystallization kinetics experiments for the unmodified blend (**UB**). (left) *i*PP domain II begins at **168 °C**; HDPE domain II begins at **166 °C**, far above the nucleation temperatures for the HDPE homopolymer.

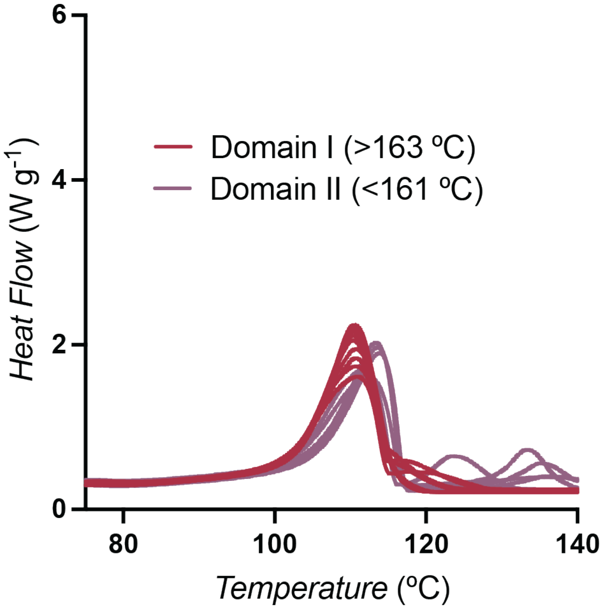

Figure S44: Crystallization kinetics experiments for the unmodified blend (**B1**). (left) *i*PP domain II begins at **161 °C**; HDPE domain II begins at **161 °C**.

g. 4D-STEM

Electron diffraction sample patterns

Example diffraction patterns are shown below in **Table S1** from 3 regions. The patterns are prepared by taking a 5x5 region of diffraction data and finding the maximum for each pixel to more clearly highlight reflections. The first (**Figure S16b**) is HDPE only, and we see overlapping reflections from (200) and the (110) lattice planes. In **Figure S16c & 16d**, we observe the (040) reflections. Based on the ratio between peaks, these weak reflections on higher scattering angles must be the (200) from HDPE.

In **Table S1**, we see the expected relative intensities of peaks for HDPE. The (040) and (130) family of reflections are at similar scattering angles, and we do not have the reciprocal resolution to distinguish these. However, the (130) family is at 65% and the (040) family is at 100% relative intensity. If we could observe the (130) family of peaks, we would also expect to see the (110) at 84% at a 6.2 Å spacing and the (131) at 60% at a 4.1Å spacing. Unfortunately, the (131) spacing overlaps with the (110) of the HDPE, and we expect to measure a few (110) and (200) reflections in the *i*PP region. However, we do not observe the (110), and the *i*PP reflections are overall much weaker than the HDPE. We cannot entirely rule out that some of the *i*PP (040) reflections should be classified as (131), but we think the majority of patterns are correctly indexed.

Principal component analysis

Principal component analysis is a dimensionality reduction technique that can be used to separate features in 4D-STEM data. While contrast in bright-field image can be quite complex, arising from a mix of factors including, crystallinity, composition, orientation, and thickness, principal component analysis can provide more images that more directly reflect crystalline phase. In our case, we produced phase maps from the principal component analysis (**Figure S17**), which were more reliably than the bright-field images due to uneven thicknesses across the field of view. The phase maps (component 2) were used to estimate the phase boundaries shown in **Figure 3**.

Domain size

The shapes of the crystalline domains in our 4D-STEM maps were very complex. While smaller domains tend to be round, larger clusters have more unusual shapes. This can be due to a variety of factors including titling of clusters out of diffraction conditions, amorphous components, and thickness effects. We estimated the domain size by measuring the tip-to-tip maximum of a given domain and then rotating the line through all projection angles and taking the max. The clusters are show in **Figure S18** as well as the histograms of domain sizes. These are area-weighted histograms such that the height of each bar is given by the number of probe positions in that domain. A histogram of the number density (just the number of each cluster) would skew towards smaller sizes. Our analysis showed a smaller domain size in the **B1** over the **UB** sample. Future work includes mapping larger fields of view for more complete statistical analysis.

Relationship between HDPE reflections

For every probe in the **B1** sample, we can calculate the relative rotation between the measured (200) and (110) reflections. We produce the histogram in **Figure S21**, which peaks just below 60 °. Based on the crystal structure of HDPE, angle of rotation between (200) and (110) in PE is 56 °. The crystallographic model in **Figure S22** shows these planes, the angle between them, and how

we expect crystalline domains to align in the PE regions. This data suggests that in many areas we are measuring two planes from the same crystal, where the crystal is aligned such that its b-axis is perpendicular to the interface, and the c-axis is along the direction of travel of the electron beam. When the angle is not close to the 56 °, then it is likely that the reflections are from separate domains that are on top of one another or next to each other.

h. Lap shear testing

The 10 mm x 25 mm strips for lap shear testing were prepared of either polyolefin or triketone functionalized polyolefin by melt pressing films of ca. 0.2 mm thickness and cutting them into 10 mm x 25 mm strips. For the preparation of the crosslinked sample, the 0.1 mol% triketone functionalized films were soaked in 1 wt% TREN in PhCl solution at 75 ºC for 10 min. PhCl was allowed to evaporate for 30 min. For both HDPE-*i*PP and crosslinked HDPE-*i*PP, melt pressing the films together was conducted at 120 ºC at 2 tons for force for 5 min using a stencil brass shim of 0.4 mm thickness to prevent squishing the sample.

Test specimens were affixed to an Instron 5566 Universal Testing Machine. Samples were pulled at a constant rate 0.16 mm s^{-1} for lap shear experiments. Force was normalized by interfacial area.

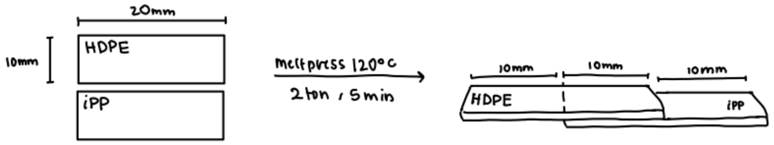

Figure S45: Schematic for the preparation of the HDPE–*i*PP interface (unmodified interface).

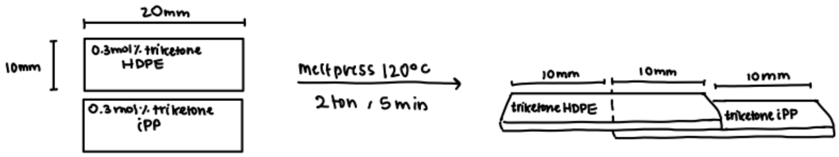

Figure S46: Schematic for the preparation of the triketone HDPE–*i*PP interface (uncrosslinked interface).

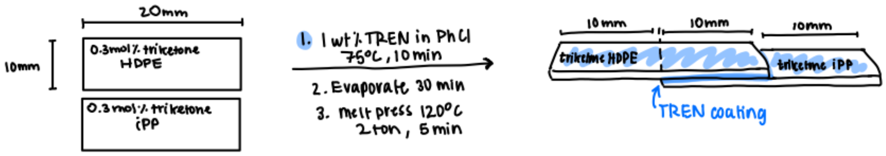

Figure S47: Schematic for the preparation of the crosslinked interface (triketone modified and TREN crosslinked HDPE–*i*PP).

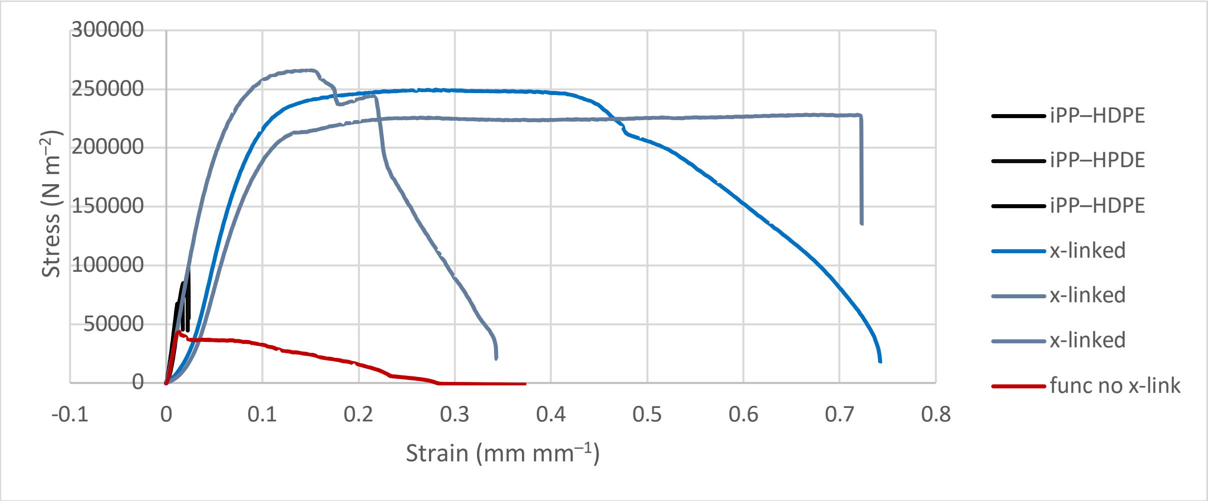

Figure S48: Test specimens were affixed to an Instron 5566 Universal Testing Machine. Samples were pulled at a constant rate 0.16 mm s^{-1} for lap shear experiments. Force was normalized by interfacial area.

i. Tensile testing

Preparation of films - on a steel plate was placed a Kapton film (Kapton KN 0.01'') (pre-treated with Frekote 770-NC) A brass shim stencil was placed to control film thickness before adding polymer. Another Kapton film was placed on top followed by a second steel plate. The setup was then heated at 200 °C for 2 min before pressing at 1000 psi for 2 h for the dynamically crosslinked blends (**B1**–**B5**). The non-crosslinked blends (**UB**, **UB2**, and **NB**) were prepared by heating at 160 °C for 2 min before pressing at 1000 psi for 5 min. Films were removed from the melt press and cooled to room temperature by rapid heat transfer to an aluminum surface.

Samples were cut into dog-bones using an ISO 527 Type 5B cutting die to standard dimensions (12mm bridge length and 2-mm bridge width). Samples were taken in random directions in the x-y plane of the film to average out potential anisotropic properties. Sample thickness at the bridge was measured using calipers. Test specimens were affixed to hand-tightened rubber grips on an Instron 5566 Universal Testing Machine with a starting gap of 18 mm. Samples were pulled at a constant strain rate of 0.09 mm s^{-1} which falls within the linear viscoelastic regime. This regime was determined by rheological frequency sweeps. Tensile experiments were conducted at room temperature in triplicate. Average values and standard deviations are reported.

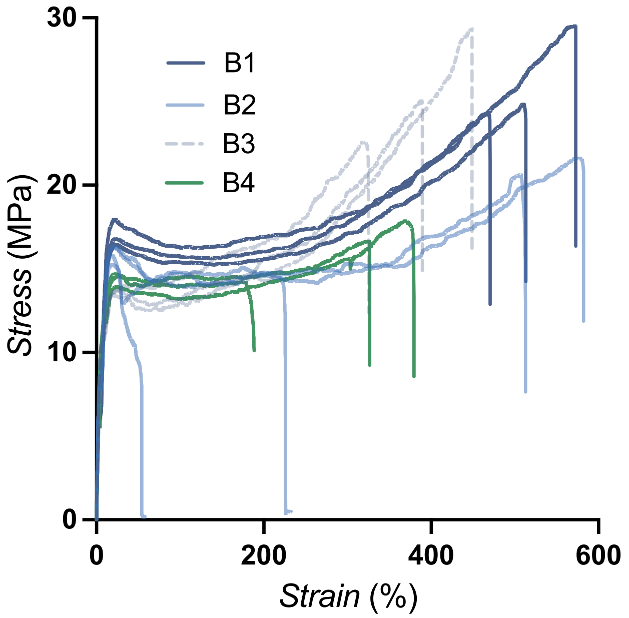

Figure S49: Tensile testing comparing the dynamically crosslinked blends **B1**, **B2**, **B3** and **B4**, conducted at 0.09 mm/s.

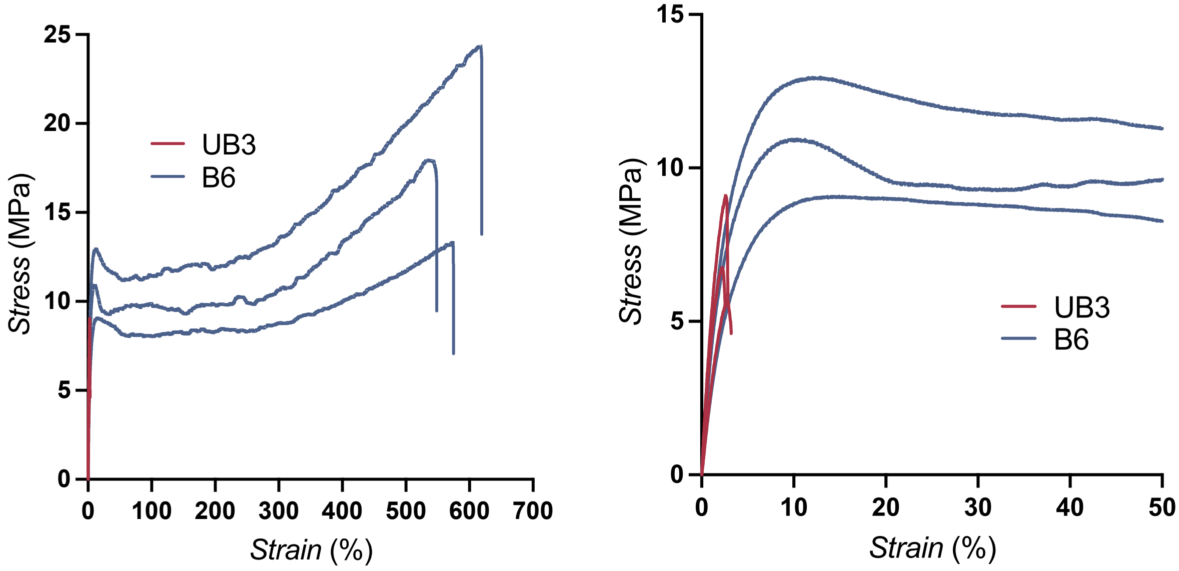

Figure S50: (left) Uniaxial tensile testing comparing the unmodified ternary blend (**UB3**) to the dynamically crosslinked ternary blend (**B6**), conducted at 0.09 mm/s; (right) zoom of the tensile curve of **UB3** and **B6**.

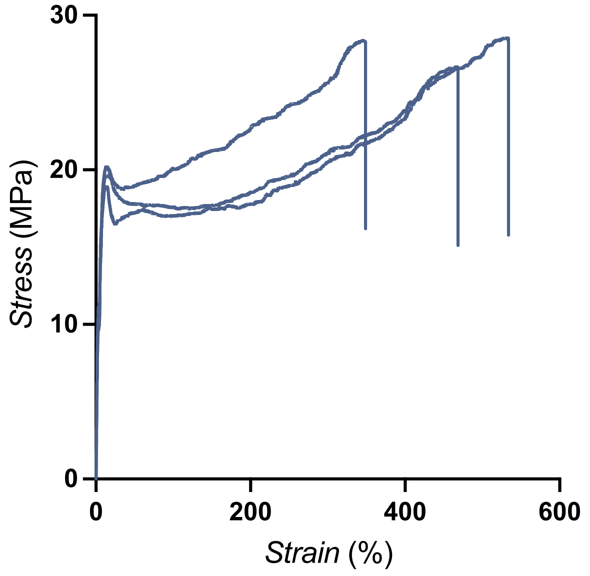

Figure S51: Uniaxial tensile testing of the dynamically crosslinked 50–50 blend of HDPE and *i*PP (**B5**), conducted at 0.09 mm s^{-1}.

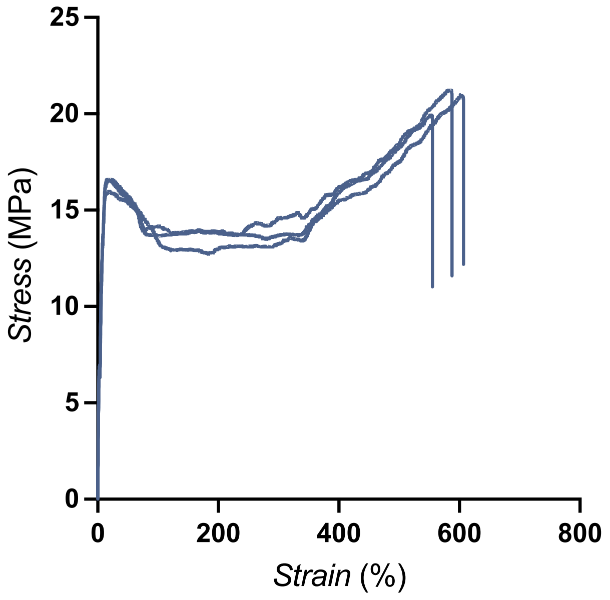

Figure S52: Uniaxial tensile testing of dynamically crosslinked HDPE functionalized at 0.3 mol%, conducted at 0.09 mm s^{-1}.

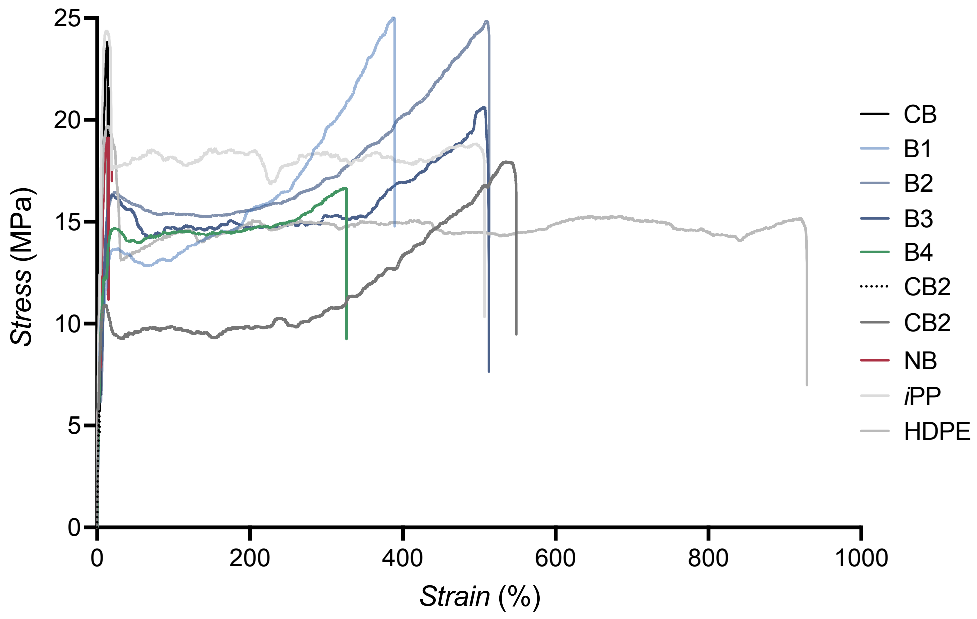

Figure S53: Tensile testing of the blends compared to HDPE and *i*PP, conducted at 0.09 mm s^{-1}.

	Strain at Break (%)	Stress at Break (MPa)	Toughness (MPa)	Young's Modulus
HDPE	277±293	13±2	93±71	726±71
i PP	463±389	20±5	73±82	451±30
Unmodified blend (**UB**)	15±2	24±0.9	1.8±1	293±16
Non-crosslinked blend (**NB**)	17±2	19±0.3	2±0.4	396±13
Dynamically Crosslinked Blend **B1**	422±204	19±4	68±33	304±7
**B2**	517±53	26±3	96±16	283±16
**B3**	387±62	25±4	65±12	258±25
**B4**	289±103	16±2	43±15	240±21
50–50 Unmodified Blend (**UB2**)	9±0.5	20±2	0.9±0.2	268±71
50–50 Dynamicaly Crosslinked Blend (**B5**)	446±92	28±1	94±17	431±31
Ternary Unmodified Blend (**UB3**)	2.5±0.3	7±2	0.1±0.03	234±188
Ternary Blend (**B6**)	581±36	18±5	58±40	291±64
Dynamically Crosslinked HDPE	581±24	20±0.7	89±5	304±12
reprocessed B1	304±196	16±3	48±30	424±31

Table S3: Table of tensile data as an average of 3 trials, conducted at 0.09 mm s^{-1}.

j. Reprocessing

Polymer films were reprocessed by cutting films into small pieces. On a steel plate was placed a Kapton film (Kapton KN 0.01'') (pre-treated with Frekote 770-NC) A brass shim stencil was placed to control film thickness before adding polymer. Another Kapton film was placed on top followed by a second steel plate. The setup was then heated at 200 °C for 2 min before pressing at 1000 psi for 2 h. Films were removed from the melt press and cooled to room temperature by rapid heat transfer to an aluminum surface. Reprocessed samples were subsequently characterized.

Reprocessed samples were cut into dog-bones using an ISO 527 Type 5B cutting die to standard dimensions (12mm bridge length and 2mm bridge width). Sample thickness at the bridge was

measured using calipers. Test specimens were affixed to the TA RSA G2k DMA with a starting gap of 18 mm for consistent pulls. Samples were pulled at 0.09 mm/s, which falls within the linear viscoelastic region. This regime was determined by rheological frequency sweeps. Tensile experiments were conducted at room temperature in triplicate. Average values and standard deviations are reported.

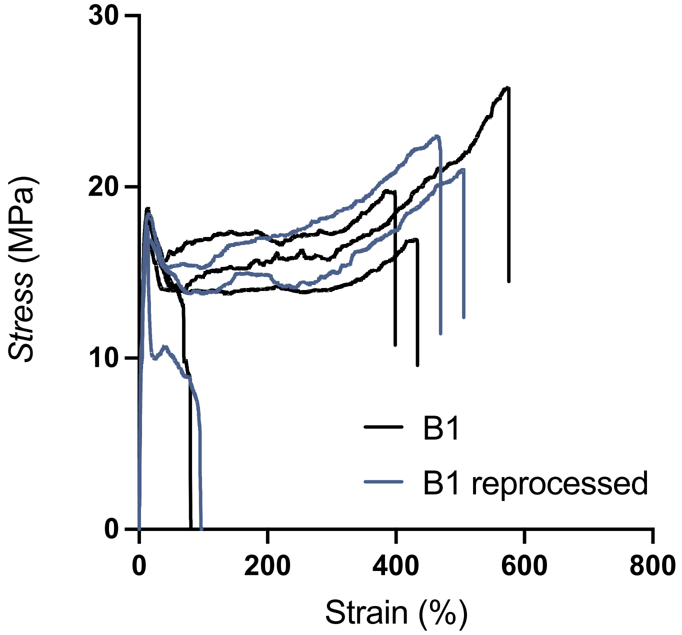

Figure S54: The tensile curves for **B1** compared to reprocessed B1(**rB1**).

k. Ball milling

Ball-milling was performed using a Retsch Planetary Ball Mill PM100. The reaction was conducted in a 50 mL zirconium oxide-coated stainless steel jar. The jar was loaded with 1.0 g of 0.3 mol% triketone functionalized HDPE and iPP in a 70–30 weight ratio, along with 20 g of 5 mm diameter zirconium oxide ball bearings. The contents were ball-milled for 5 min at 500 rpm to mix the HDPE and iPP. TREN was then added to the mixture at 2 eq. amine per triketone (9.1 mg, 0.062 mmol), and ball-milling was conducted in two 30-min interval. After each interval, the jar was opened and the sides were scraped down. The solids were then separated from the ball bearings to recover 0.83 g of product.

The crosslinked product was melt-pressed at 200 °C for 2 h using an ASTM D638 type V dogbone mold. Gel fraction was determined using portions of the melt-pressed material. Samples were soaked in 4 mL of 1,2,4-trichlorobenzene at 135 °C for 48 h. The solvent was removed and replaced with fresh solvent 3 times in each 24 h period. After 48 h, the samples were cooled to ambient temperature and the solvent was replaced with dichloromethane. Samples were soaked for 24 h with 3 solvent exchanges to remove residual trichlorobenzene. The samples were vacuum dried

and the gel fraction was determined to be 74 ± 2% using the ratio of residual solids to the original sample mass.

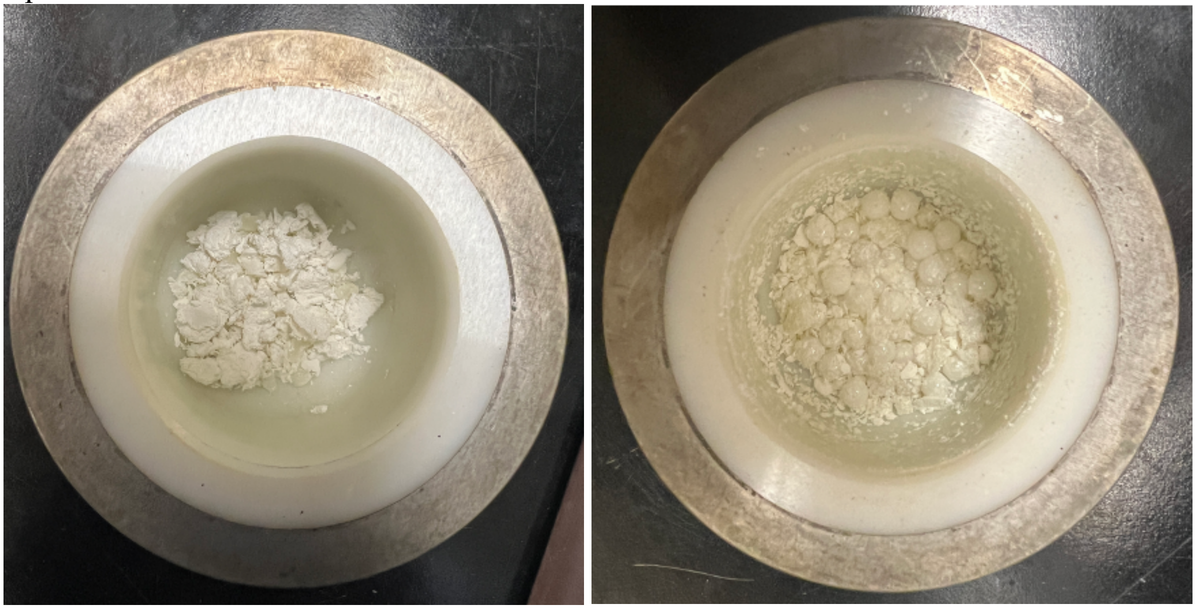

Figure S55: (left) 1 g of 0.3 mol% functionalized HDPE and *i*PP in a 70–30 ratio before ball milling; (right) 1 g of 0.3 mol% functionalized HDPE and *i*PP in a 70–30 ratio after ball milling for 5 min to mix the polymers.

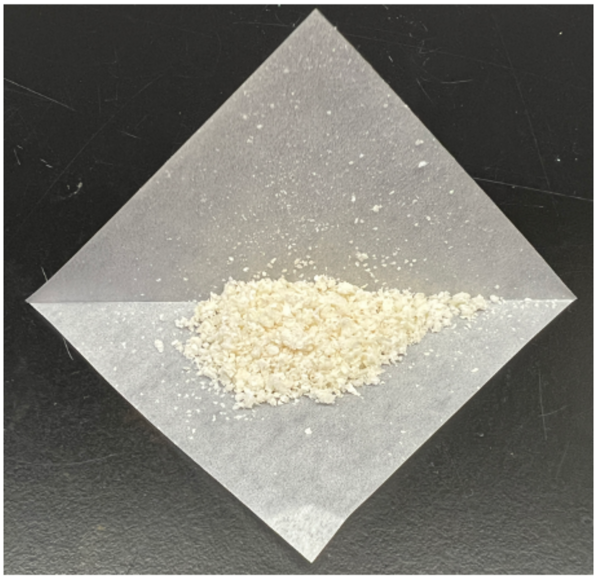

Figure S56: Post-reaction with 2 eq of reactive R–NH_2 of TREN for 2 x 30 min.

l. Solid state NMR

^1H echo 1D NMR spectra were acquired on a 16.4 T magnet (corresponding to a ^1H Larmor frequency of 700 MHz), using a 3.2 mm Bruker triple channel $^1H/^{13}C/^{15}N$ probe at a magic angle spinning (MAS) rate of 23 kHz. The T1ρ relaxation time was measured with a standard spin-lock experiment (90° pulse immediately followed by a phase shifted variable-length spin-locking pulse)

under two conditions. For the overall spectrum, T1ρ was measured on a 16.4 T magnet with RF spin-lock field of 48 kHz. For a site-specific T1ρ measurements at a selected resonance, experiments were conducted on a 9.4 T magnet (Bruker NEO400), using a Bruker BioSpin spectrometer equipped with an Avance IV Neo console with a 3.2 mm double resonance HX MAS probe, which allows for longer spin-lock duration at higher spin-lock field strength. The measurements employed RF spin-lock fields of 60 kHz with samples spun at a MAS rate of 20 kHz. All experiments were conducted at 25 °C at the Pines Magnetic Resonance Center.

All the time constants have slightly decreased, though this may be within the uncertainty of the ILT analysis. We also note the general broadening of all three peaks for the **B1** sample as compared to **NB**, suggesting greater heterogeneity or blending across motional environments.

We now turn to the high-resolution proton NMR spectra that were obtained at 16.4 T. Here the **UB** sample exhibits broad resonances characteristic of both crystalline and amorphous regions, the **B1** and **NB** samples show additional resonances attributed to polymer-bound functionalized triketones. These peaks appear at 18.1, 13.7, 0.19, and 0.1 ppm. Specifically, the resonances at 18.1 and 13.7 ppm are associated with the enol protons and enamine protons, respectively.[2] The smaller chemical shifts around 0.1-0.2 ppm remain unassigned.

Given the peaks at ~18 ppm, we surmise that some enol protons remain unreacted, i.e., not all triketones participate in reaction with the amine groups. Although the signals corresponding to functionalization are relatively weak in comparison to the polymer backbone peaks, they are distinguishable from the intense and broad resonances of the polymer backbone. The **NB** (non-crosslinked blend) sample displays a clearer and more intense enamine resonance along with a broad and weak enol signal. In contrast, **B1** (dynamic crosslinked blend) shows a greater defined enol resonance but a weaker and broader enamine resonance. This supports that **B1** reacts less with the amine crosslinker, perhaps due to the constraints of a more confined network in comparision to **NB**. Given that the enol proton is well-resolved and assignable in **B1**, we use this resonance as a marker to investigate the mobility in the crosslinked via a T1ρ measurement of the enol protons environment.

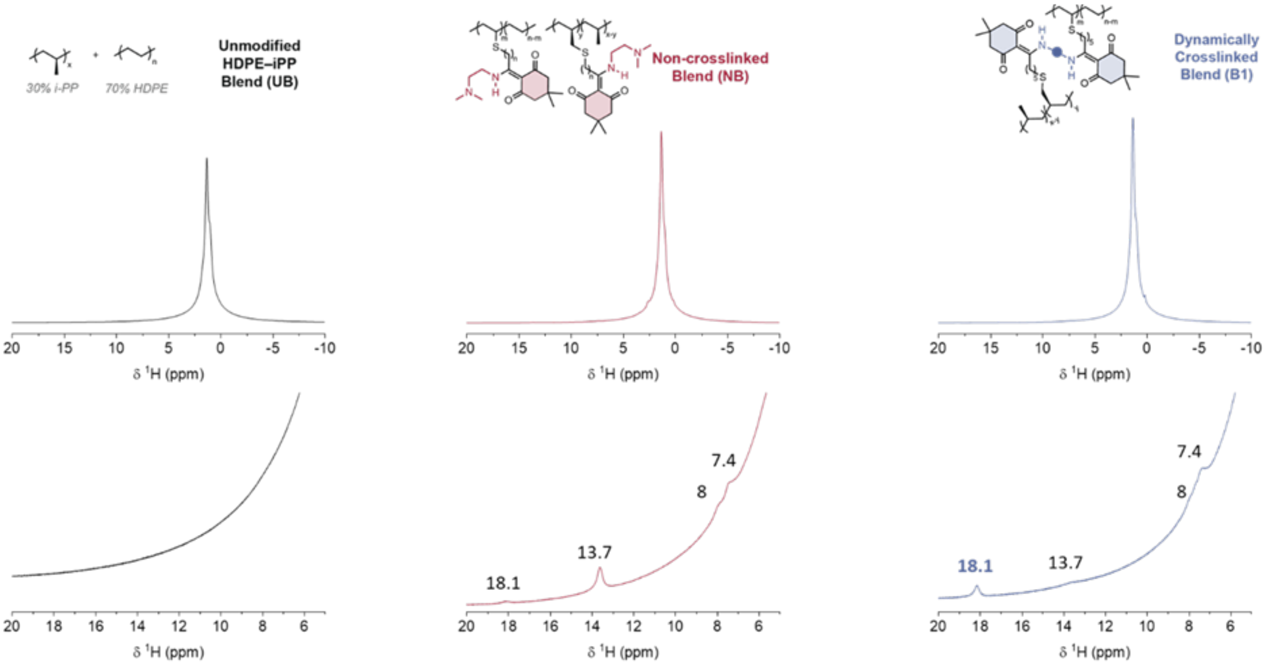

Figure S57: Comparative ^{1}H spectra for the UB (left), NB (center) and B1 (right) samples, both measured at 23 kHz under full saturation conditions. The upper panel shows the full ^{1}H spectrum for each sample and the lower panel shows a range from 20 – 5 ppm.

For the **B1** sample with dynamic crosslinking, the T1ρ decay c.a. 18.1 ppm exhibits biexponential behavior (signal averaged around 7.5 hours). Comparative analyses from multi-exponential NNLS fitting and Laplace inversion yield two T1ρ values of 4 ms (85%) and 69-77 ms (15%). Spatially locating the enol proton from these relaxation values is complicated by the fact that the enol protons at 18 ppm are unreacted triketones.

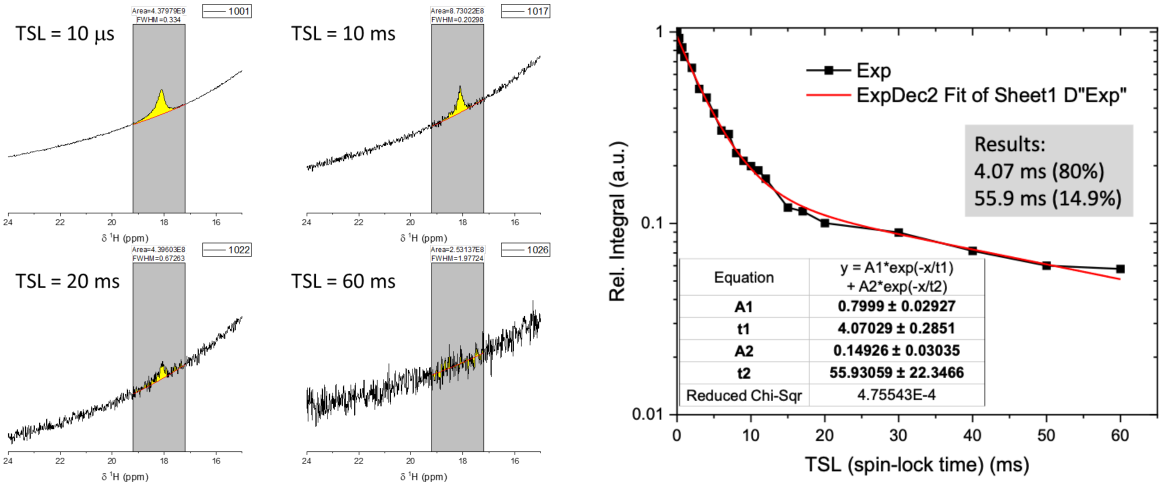

Figure S58: T1ρ measurement of the **B1** sample with a spin lock of 60 kHz over varied spin lock times (TSL). The decay of the 18.1 ppm resonance as a function of spin lock time was fit to extract T1ρ (shown at right, two separate experiments).

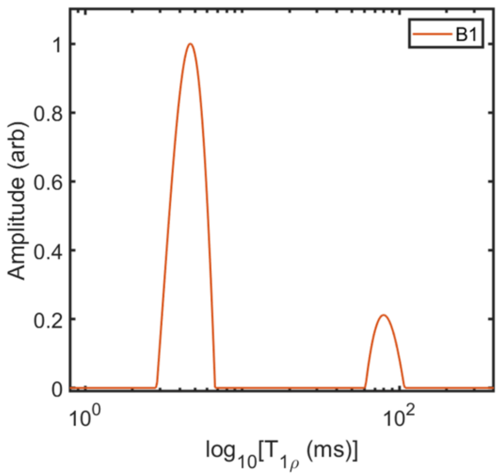

Figure S59: T1ρ distribution of the 18.1 ppm (PDK O-H) peak for the **B1** sample calculated via Laplace inversion.

V. NMR's of Novel Compounds functionalized polymers small molecules

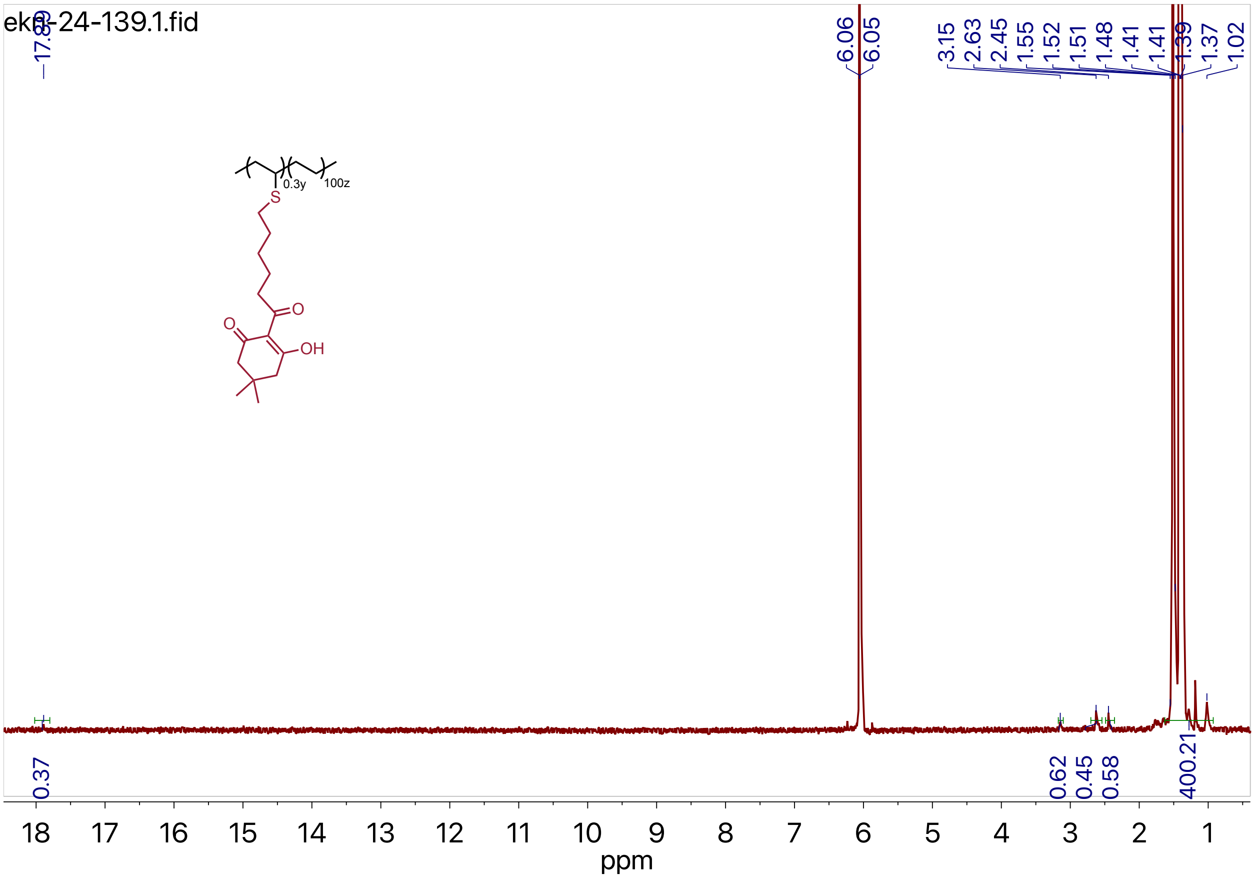

HDPE-0.3: ^{1}H NMR (500 MHz, $C_2D_2Cl_4$)

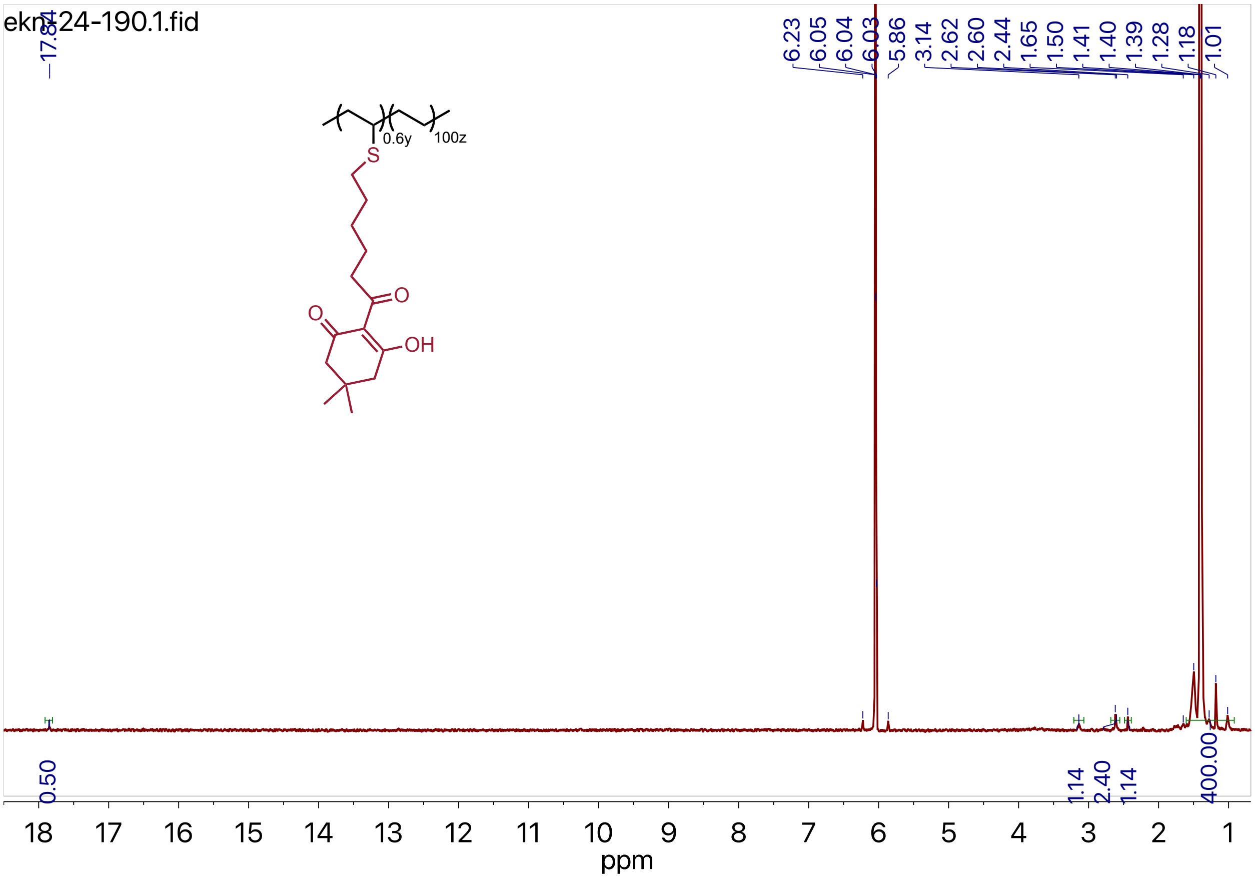

HDPE-0.6: ^{1}H NMR (500 MHz, $C_2D_2Cl_4$)

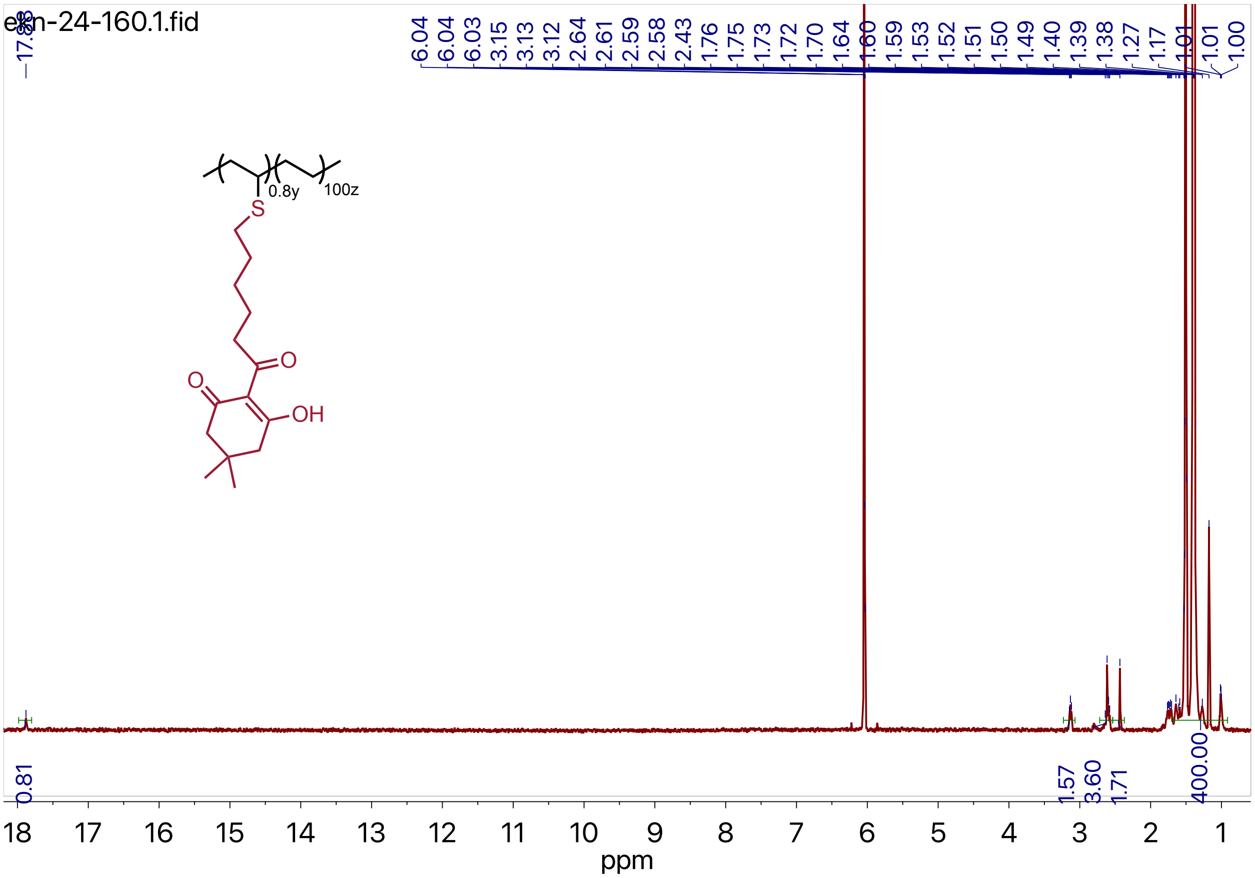

HDPE-0.8: ^1H NMR (500 MHz, $C_2D_2Cl_4$)

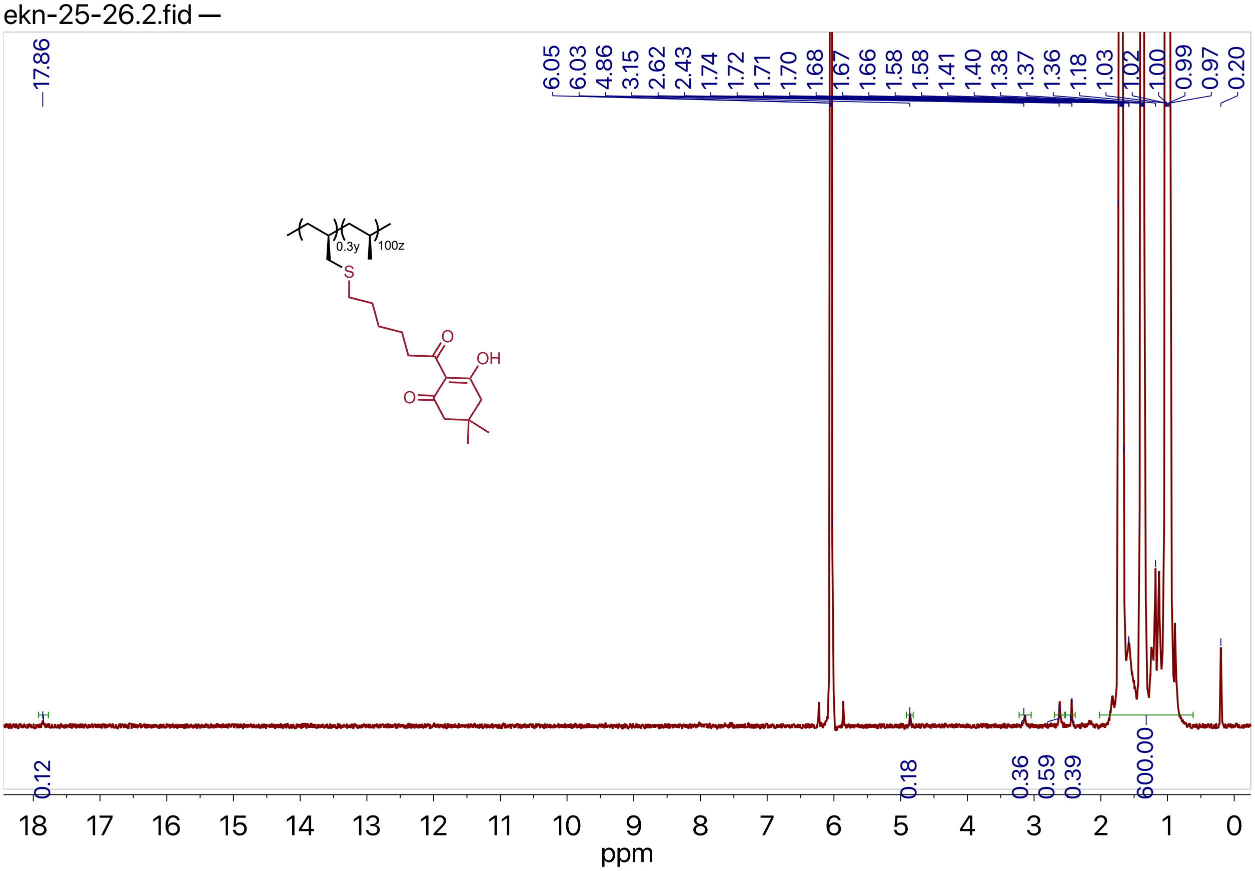

***i*PP-0.3**: ^{1}H NMR (500 MHz, $C_2D_2Cl_4$)
